# Effects of an Alternating Magnetic Field towards Dispersion of α-Fe_2_O_3_/TiO_2_ Magnetic Filler in PPO_dm_ Polymer for CO_2_/CH_4_ Gas Separation

**DOI:** 10.3390/membranes11080641

**Published:** 2021-08-20

**Authors:** Yun Kee Yap, Pei Ching Oh

**Affiliations:** 1 Department of Chemical Engineering, Universiti Teknologi PETRONAS, Seri Iskandar 32610, Perak, Malaysia; 21878_19001046@utp.edu.my; 2 CO2 Research Centre (CO2RES), R&D Building, Universiti Teknologi PETRONAS, Seri Iskandar 32610, Perak, Malaysia

**Keywords:** mixed matrix membrane, magnetic particle, filler dispersion, agglomeration, dispersion quantification, alternating magnetic field, gas separation

## Abstract

Magnetic-field-induced dispersion of magnetic fillers has been proven to improve the gas separation performance of mixed matrix membranes (MMMs). However, the magnetic field induced is usually in a horizontal or vertical direction. Limited study has been conducted on the effects of alternating magnetic field (AMF) direction towards the dispersion of particles. Thus, this work focuses on the incorporation and dispersion of ferromagnetic iron oxide–titanium (IV) dioxide (αFe_2_O_3_/TiO_2_) particles in a poly (2,6-dimethyl-1,4-phenylene) oxide (PPO_dm_) membrane via an AMF to investigate its effect on the magnetic filler dispersion and correlation towards gas separation performance. The fillers were incorporated into PPO_dm_ polymer via a spin-coating method at a 1, 3, and 5 wt% filler loading. The MMM with the 3 wt% loading showed the best performance in terms of particle dispersion and gas separation performance. The three MMMs were refabricated in an alternating magnetic field, and the MMM with the 3 wt% loading presented the best performance. The results display an increment in selectivity by 100% and a decrement in CO_2_ permeability by 97% to an unmagnetized MMM for the 3 wt% loading. The degree of filler dispersion was quantified and measured using Area Disorder of Delaunay Triangulation mapped onto the filler on binarized MMM images. The results indicate that the magnetized MMM presents a greater degree of dispersion than the unmagnetized MMM.

## 1. Introduction

Filler agglomeration has often been reported to worsen the gas separation performance of mixed matrix membranes (MMMs) [1]. This phenomenon is typically attributed to the nature of nano or submicron-sized fillers, wherein the attraction between particles is governed by their strong van der Waal forces, hydrogen bonds, or high surface energy [2]. Researchers often introduce various methods to reduce agglomeration or improve the dispersion of fillers via the physical or chemical modification of the filler (e.g., priming, mechanical dispersion, covalent or non-covalent functionalization, dual fillers). However, each of these methods has its respective drawbacks. Priming only reduces agglomeration up to the maximum weight loading of the filler, mechanical dispersion places the filler at risk of being damaged, and functionalization and dual fillers require the right synthesis and pairing of materials to work effectively [3,4,5,6]. Besides the conventional method of filler dispersion, several works have been published on the implementation of a magnetic field to manipulate the alignment or dispersion of magnetic fillers to improve the gas separation performance. Rybak worked on iron-encapsulated carbon nanotubes that were dispersed in poly (2,6-dimethyl-1,4-phenylene oxide) (PPO_dm_) in the absence and presence of a magnetic field. The magnetic fields were supplied using two ferrite magnets or a magnetic coil. It was claimed that filler alignment occurred, resulting in improved gas separation performance [7]. Several other works also implement similar concepts by exposing their cast MMM to the external field of the coil. The typical magnetic field directions were horizontal and vertical to align the fillers for improved selectivity [7,8,9,10,11,12]. In some cases, it also prevented filler sedimentation in the polymer phase [8].

However, there is a limited number of studies on the effects of alternating magnetic field (AMF) ‘direction’ towards the deagglomeration or dispersion of fillers in the area of the MMM for gas separation. An AMF was said to induce heat and a mechanical action onto the ferromagnetic particles. Expediting the rotation and translation of magnetic particles via an AMF, the application of an AMF has also commonly been studied in biomedical technology, especially for cancer treatment [13]. The researchers often employ iron oxide nanoparticles to target and destroy cancer cells via frictional heat generated from the nanoparticles’ Brownian and Neel relaxation mechanisms. A study has also been performed on controlled dispersion and agglomeration of nanoparticles in a biofluid for potential biomedical applications via manipulation of the AMF strength, exposure time, frequency of the field, and various other parameters [14]. We hypothesize that an AMF could improve the dispersion and reduce the agglomeration of fillers in a MMM, resulting in enhanced gas separation performance.

In this paper, we aim to elucidate the effects of an AMF on the distribution of filler in the polymer phase and identify the correlation of the filler distribution in the MMM with their respective gas separation performances. We incorporated magnetic iron oxide–titanium dioxide (α-Fe_2_O_3_/TiO_2_) composite particles into the polymer phase. TiO_2_ filler represents the intended substrate for the growth of the α-Fe_2_O_3_ structure to induce the movement and dispersion of TiO_2_ particles in the polymer matrix. Additionally, researchers commonly characterize the distribution of fillers qualitatively (e.g., SEM, XRD, TEM, AFM), and that is inadequate to provide in-depth details about the distribution of fillers. A quantitative dispersion analysis of the MMM should be conducted to aid in the study of the filler distribution via Delaunay’s Triangulation, a robust dispersion analysis method developed by Bray and his colleagues [15,16,17]. To the best of the authors’ knowledge, these MMM combinations and magnetic field direction applications have not been attempted to date. The results show the improved distribution of fillers within the polymer matrix, accompanied by an increment in the gas separation performance in terms of selectivity.

## 2. Materials and Methods

### 2.1. Materials

Poly (2,6-dimethyl-1,4-phenylene oxide) (PPO_dm_) polymer powder, titanium (IV) oxide (TiO_2_, >99.5% purity) nanopowder, and ethanol (C_2_H_6_OH, >99.5% purity) were purchased from Sigma Aldrich. Iron (III) chloride hexahydrate (FeCl_3_.6H_2_O) and chloroform (CHCl_3_, >99.5% purity) were purchased from Merck. Carbon dioxide (CO_2_) and methane (CH_4_) gases were supplied by Air Products, Malaysia, at 99.995% purity. All chemicals were used without further purification.

### 2.2. Filler and Membrane Fabrication

#### 2.2.1. Synthesis of the α-Fe_2_O_3_/TiO_2_ Magnetic Filler

The molar ratio of titanium dioxide (TiO_2_) to iron (III) chloride hexahydrate (FeCl_3_·6H_2_O) was 1:15. TiO_2_ was added into a 0.5 M FeCl_3_·6H_2_O solution and ultrasonicated for 30 min. The solution was stirred for 30 min before being placed into a hydrothermal autoclave for 4 h at 95 °C. The slurry was then cooled to room temperature and transferred to the centrifugal tube. The slurry in the tube was centrifuged thrice with deionized water and ethanol. Then, the slurry was dried in a vacuum oven at 80 °C overnight. The dried particles were annealed in the furnace for 2 h at 500 °C at a heating rate of 2 °C/min. Lastly, the particles were dried in the vacuum oven at 80 °C for 24 h for moisture removal.

#### 2.2.2. Membrane Fabrication

**Figure 1:**
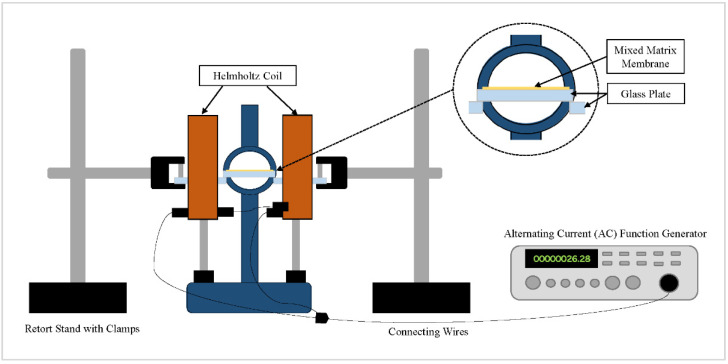
Helmholtz coil setup for the magnetization of the MMM via an AMF.

The fabricated membranes are displayed in Table 1. PPO_dm_ and α-Fe_2_O_3_/TiO_2_ particles were dried in a vacuum oven at 80 °C overnight to remove moisture. Next, α-Fe_2_O_3_-TiO_2_ filler equivalent to PPO_dm_ wt% was added to the chloroform and sonicated for 30 min. PPO_dm_ of 22.0 wt% to chloroform’s weight was gradually added to the suspension while being stirred for the next 24 h at 60 °C using a magnetic hotplate stirrer. The dope solution was degassed for 4 h and left standing overnight at room temperature. The solution was then spin-coated onto a 5 × 5 cm glass plate at 1000 rpm for 42 s at a ramp-up speed of 125 rpm/s. Membranes were then left to dry for 24 h at room temperature in a closed container for controlled solvent evaporation. They were dried for another 72 h in the vacuum oven at 65 °C to remove any residual solvent. As for the pristine membrane’s fabrication, there was no addition and sonication of filler in the dope solution before casting. For the magnetized MMM, the spin-cast membrane was subsequently placed at the center of the Helmholtz coil. The function generator was set to maximum current beforehand, producing an alternating magnetic field (AMF) of 10 Gauss (G). The frequency was then gradually increased to the maximum at 330 kHz. The MMM was magnetized for 5 min and subsequently dried as per the above-described procedure. The AMF was generated using a sine wave in an AC circuit. Figure 1 depicts the setup of the Helmholtz coil.

### 2.3. Characterization of MMMs

The incorporation of α-Fe_2_O_3_/TiO_2_ particles was investigated by X-ray diffraction (XRD) using X’Pert3 Powder and an Empyrean instrument (PANalytical). The X-ray patterns were obtained at room temperature by using Cu Kα X-ray radiation from 20° to 80° with an increment of 0.026° and an exposure time of 0.2 s/step.

The particle size analysis was carried out using a Malvern Master Sizer 2000 particle size analyzer. The analysis was carried out with α-Fe_2_O_3_/TiO_2_ particles dissolved in chloroform as the medium. The particle size distribution analysis was carried out three times to ensure the accuracy of the size distribution.

The magnetic properties of α-Fe_2_O_3_/TiO_2_ particles were measured using a LakeShore 340 Series vibrating sample magnetometer (VSM) along with IDEAS-VSM Version 4 software. The sample was magnetized in the range −8000 Oersted to +8000 Oersted.

The surface images of MMMs were captured via an Optical Microscope (OM, Olympus BX53M) integrated with Olympus Stream, and images were taken with an Olympus LC30 Camera. Five times magnification was set, and the images were captured via transmitted light. The images are shown in the supplementary files (Figures S1–S54). Figures S1–S27 show the magnetized MMM samples, and the remainder show the unmagnetized samples. The letters a and b denote the binarized and non-binarized images of MMMs, respectively. For each MMM of a particular filler loading, there are nine captured images.

### 2.4. Quantitative Analysis of Filler Dispersion in MMMs

The images captured via the OM were processed via a MATLAB algorithm and quantified by measuring the area disorder of the Delaunay Triangulation (*AD_Del_*) mapped onto fillers dispersed throughout the membrane. The *AD_Del_* is a dimensionless quantity between 0 and 1 and is numerically expressed as:

(1) $${AD}_{Del}=1-{\left(1+S_{Ad}/\bar{A_{D}}\right)}^{-1}$$

In a finite system, the sample mean area of a Delaunay triangle is denoted as $\bar{A_{D}}$ and the sample standard deviation as $S_{Ad}$. An *AD_Del_* value of 0 represents a perfectly dispersed system of a lattice of particles, whereas 1 depicts the opposite.

**Figure 2:**
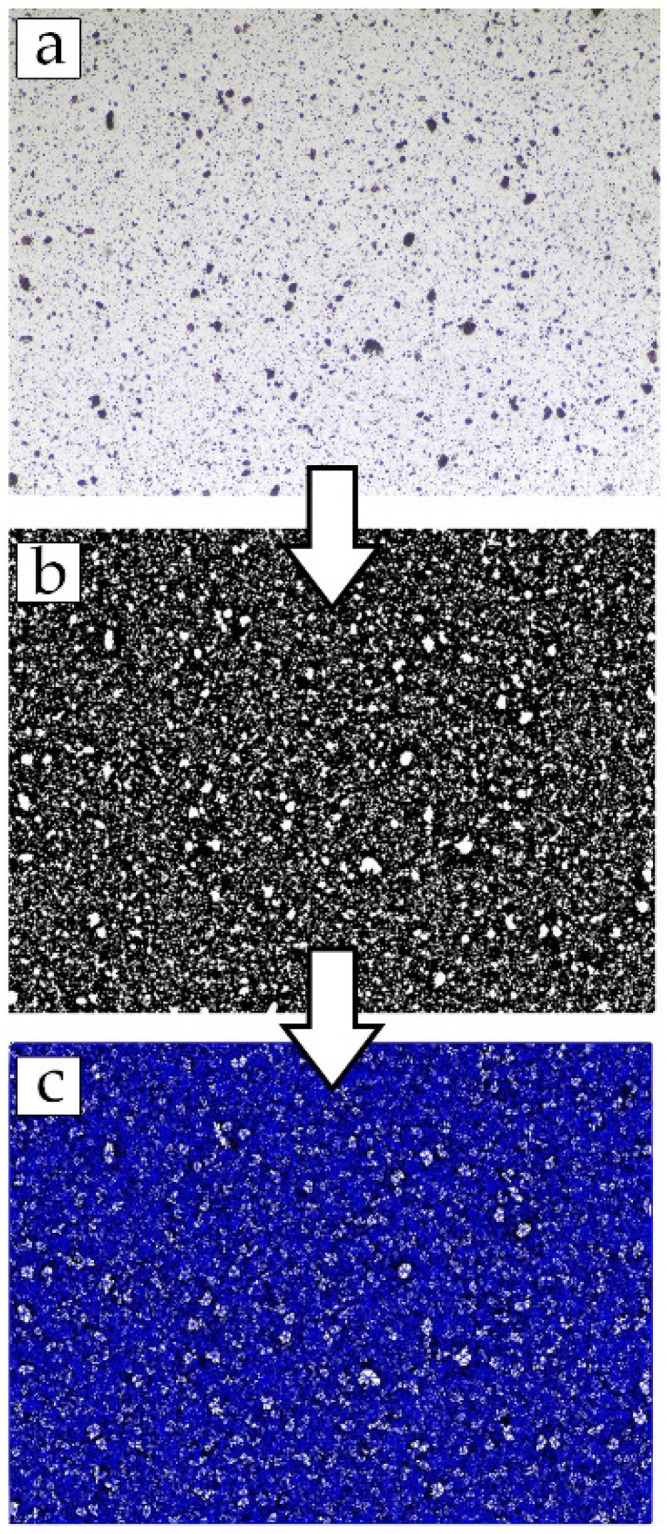
Simplified image processing steps: (**a**) Surface of the MMM captured by the OM; (**b**) The image was binarized; (**c**) The Blue Delaunay network was mapped onto the center of mass of each detected particle.

The steps involved in processing the images for *AD_Del_* calculation begin with converting images into grayscale images and subsequently converting those into binary images. Blacks are the polymer phase, and white depicts the fillers. An adaptive threshold method via the median statistical operator with a kernel size of 90 pixels and a threshold of 0.01 was applied to obtain the black and white images. Then, fillers were identified, and their center of mass was generated based on their position in the image. Virtual particles were also generated and distributed uniformly across the image’s border to account for appropriate boundary conditions and improve the dispersion value’s accuracy. The network of Delaunay triangles was then mapped onto the center of mass spread across the membrane image. The process is presented in Figure 2. The areas of each Delaunay triangle were then calculated, and their mean and standard deviation were obtained to quantify the dispersion of fillers, *AD_Del_,* in the images captured. The area fraction of fillers, the number of particles, and the average particle size based on the area of triangles from the mapped Delaunay network were measured using MATLAB. Nine images from random locations of each MMM sample were captured and processed using MATLAB to obtain the mean and standard deviation, respectively. The MATLAB codes are shown in Appendix B. The version of MATLAB used was MATLAB R2020A.

A statistical two-sided z-test was carried out to compare the *AD_Del_* values of magnetized and unmagnetized MMM samples with a similar filler loading to that observed if the magnetized samples’ dispersion values result in rejection of the null hypothesis at a 5% significance level. If they do, it proves that the *AD_Del_* value of the magnetized MMM samples is determinate rather than random. Further information can be found in Bray’s work [17]. Equation (2) shows the formula used to calculate the Z value of *AD_Del_*.

Z = (*AD_Del_*(test) − E(*AD_Del_*(*HR*)))/(*S_AD_*(HR)) (2)

*AD_Del_*(test) is the measurement from the material, and E (*AD_Del_*(*HR*)) and *S_AD_*(HR) are the mean and standard deviation, respectively, of the null-hypothesis HR model.

### 2.5. Gas Permeation Test

**Figure 3:**
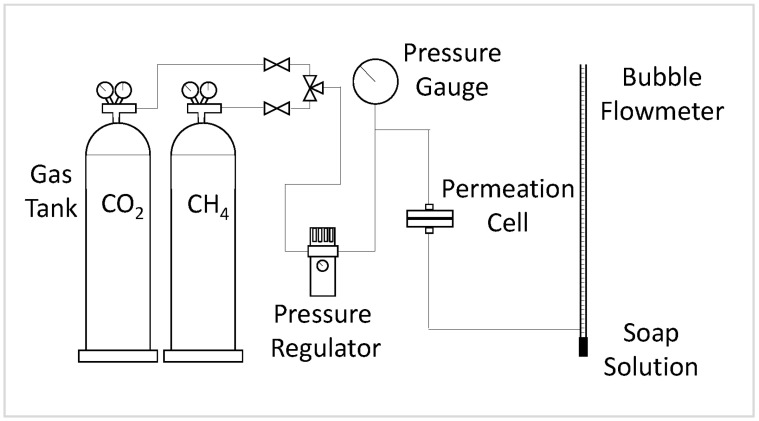
Setup of the gas permeation rig.

The gas permeation test was carried out using a gas permeation rig built in-house. The rig was first vacuumed for a minimum of 30 min to remove moisture in the system. Then, membrane samples were cut to a size of 6.16 cm^2^ and placed inside the gas permeation test cell. The membrane was degassed for a minimum of 30 min to remove any trapped gases before starting the gas permeation test. An external vacuum pump degassed the membrane via a tube connected from the permeate side of the permeation cell to the vacuum pump. Pure CO_2_ and CH_4_ gas tests were conducted separately at a static pressure of 3.5 bar and room temperature (296K) after equilibrium was reached in the system. The flow rate of the permeate was measured using a soap bubble flowmeter. The permeability and selectivity were calculated via Equations (3) and (4).

(3) $$\frac{{\left(P\right)}_{i}}{l}=\frac{Q_{i}}{\Delta p_{i}A}\left(\frac{273.15}{T}\right)$$

(4) $$\alpha_{{\mathrm{CO}}_{2}{/\mathrm{CH}}_{4}}=\frac{P_{{\mathrm{CO}}_{2}}}{P_{{\mathrm{CH}}_{4}}}$$

where (*P*)*_i_* is defined as the permeability of gas *i* in Barrer, *Q_i_* is the volumetric flow rate of gas *i*, Δ*p* is the pressure difference across the membrane, *A* is the membrane’s effective surface area, and l is the membrane skin’s thickness. The permeability of CH_4_ and CO_2_ are reported in Barrer units (1 Barrer = 1 × 10^−10^ cm^3^ (STP) cm/s cm^2^ cmHg). The selectivity, *α*, of the membrane for pure gas and negligible downstream pressure was then obtained by dividing the permeability of CO_2_ by the permeability of CH_4_. Figure 3 shows the setup of the gas permeation rig.

## 3. Results

### 3.1. Characterization of Inorganic Particles

**Figure 4:**
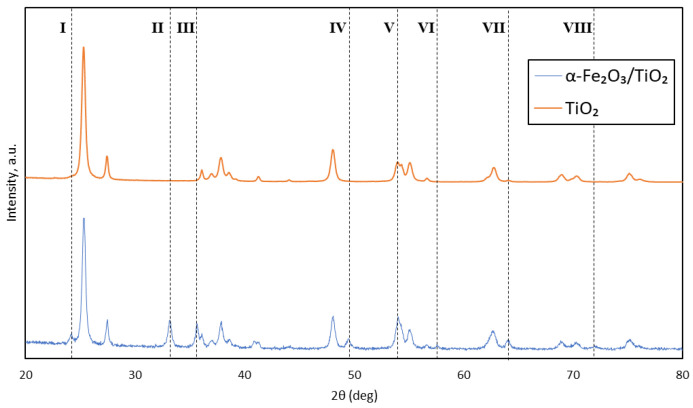
XRD pattern of TiO_2_ and α-Fe_2_O_3_/TiO_2_ particles.

The desired synthesis of magnetic composite α-Fe_2_O_3_/TiO_2_ particles was evidenced by the change in the XRD pattern when compared with pristine TiO_2_ particles. There are eight notable pattern changes based on Figure 4. The presence of hematite (α-Fe_2_O_3_) was supported by the appearance of major peaks from angle I to VIII (24.17°, 33.17°, 35.65°, 49.47°, 57.57°, 63.89°, and 71.95°). Pattern V shows an increase in intensity from pristine TiO_2_ particles, denoting the presence of α-Fe_2_O_3_. An anatase nanocrystalline and rutile structure was also identified based on the remaining peaks. The results are related to the Inorganic Crystal Structure Database (ICSD) Nos. 184766 and 154604 for the α-Fe_2_O_3_ and TiO_2_ structures, respectively. In addition, it was notable that the formation of hematite did not affect the structure and crystallinity of α-Fe_2_O_3_/TiO_2_ particles. These major α-Fe_2_O_3_ peak patterns agree with a few other works of literature that fabricated similar α-Fe_2_O_3_/TiO_2_ composite particles [18,19,20,21]. The formation of a magnetic α-Fe_2_O_3_ counterpart is vital for the movement of the composite particles via the magnetic field.

**Figure 5:**
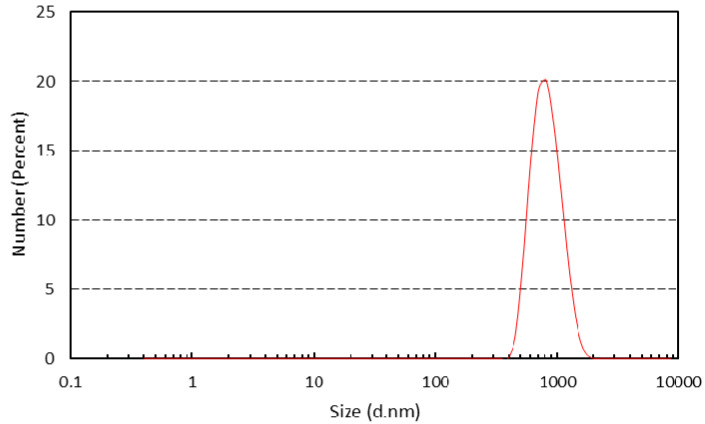
Particle size distribution of α-Fe_2_O_3_/TiO_2_.

Another property of the inorganic particles vital to this study was the particle size of the α-Fe_2_O_3_/TiO_2_ composite. Based on Figure 5, the diameter range of the particles was within 531 nm to 1720 nm, placing the synthesized particles into the submicron (100 to 1000 nm) category. The z-average diameter was observed to be approximately 884 nm. Based on the Debye–Scherrer Equation and assuming that the shape constant was spherical, the crystallite diameter was calculated to be at an average size of 25 nm. However, the substantial growth in particle size from pristine commercial TiO_2_ may be attributed to the agglomeration phenomenon in the chloroform solvent during the particle size characterization or due to large formations of the respective hematite. The agglomeration measured was composed of smaller nano-crystallites. The particle diameter was used to determine the particle volume for the Brownian relaxation time calculation in the next section.

**Figure 6:**
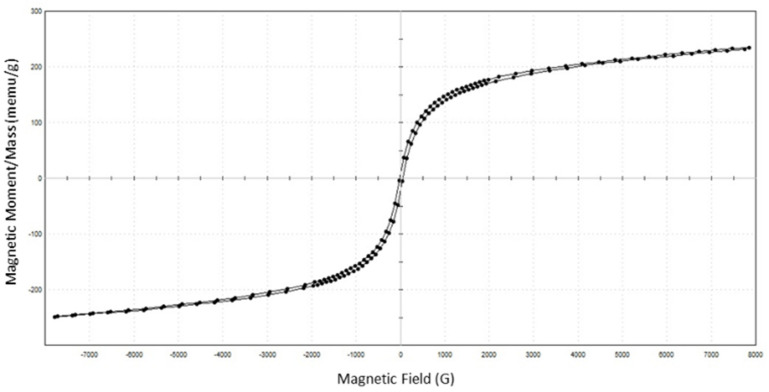
Magnetization versus magnetic field (M–H) curves of α-Fe_2_O_3_/TiO_2_ particles.

The magnetic properties of the synthesized α-Fe_2_O_3_/TiO_2_ magnetic submicron particles (MSPs) were measured using a vibrating sample magnetometer (VSM). Figure 6 presents the hysteresis loop of MSPs measured at room temperature. The saturation magnetization (*M_s_*), retentivity (*M_r_*), and coercivity (*H_c_*) of MSPs were measured to be 0.24146 emu/g, 10.082 × 10^−3^ emu/g, and 34.64 Gauss (*G*), respectively. The presence of a hysteresis loop, albeit narrow, denotes that the MSPs have ferromagnetic properties, indicating the ability of a substance to retain magnetism in the absence of an external magnetic field. This is undesirable, as particles dispersed during the solvation of a polymer via a magnetic stirrer may attract amongst themselves in the absence of the magnetic field (i.e., the dope solution at a stationary phase with the absence of the magnetic field for the removal of air bubbles). However, the circumstances may not lead to severe re-agglomeration or attraction of particles due to their shallow values of retentivity, which minimizes particle agglomeration [22,23]. Superparamagnetic particles that exhibit zero remanence magnetization would be the ideal property. It would require further study to tune the nano-size growth of the magnetic counterpart to the current commercial pristine TiO_2_. The saturation magnetization and coercivity are on the lower end of the range compared with other works, but these values were also attained by Mahajan and Jeevanandam [18].

The area of the hysteresis loop also represents the maximum heat that the magnetic particles can generate. The heat is generated either by hysteresis loss or Brownian and Neel relaxation times. In our case, the heat dissipation mechanism was assumed to be governed by the Brownian relaxation time due to our large particle size and the fact that it was suspended in a viscous solution [24]. The Brownian relaxation time refers to the time required for the rotation of the entire magnetic particle to align its magnetic moment to the direction of the induced magnetic field, which was the AMF in our case. Due to the relatively large size of the particles synthesized in our study, Neel’s relaxation time was ruled out as it only occurs with nanoscale particles, which are superparamagnetic. As for hysteresis loss, it was ruled out because it generally requires a magnetic field amplitude of at least two times the coercivity of the particle [25]. The amplitude of the AMF in our study was 10 G, whereas the coercivity of the particle was measured to be 34.64 G. Since the heat dissipation was mainly generated by the Brownian relaxation phenomenon in our case, it is of interest to maximize the amount of heat dissipation to increase the rate of rotational movement by Brownian relaxation from the MSP fillers. There is an optimal peak frequency, *f_p_,* that maximizes the heat dissipation based on the Brownian relaxation time, *π_B_*, and is represented through Equation (5), which was adapted from Mamiya and Jeyadevan [26]. *µ*, *µ*_0_*, H_ac_, K_B_*, and *T* represent the magnetic permeability, the permeability of free space, the amplitude of the alternating magnetic field, the Boltzmann constant, and the temperature of the solution.

(5) $$2\pi f_{P}=\pi_{B}^{-1}{\left[1+0.07{\left(\frac{\mu .\mu_{0}H_{ac}}{K_{B}T}\right)}^{2}\right]}^{0.5}$$

The Brownian relaxation time formula displayed in Equation (6) can be calculated using the standard formula available in most studies. *η* and *V_H_* represent the viscosity of the solution and hydrodynamic volume of the particle, respectively.

(6) $$\pi_{B}\;=\;\frac{3\eta V_{H}}{K_{B}T}$$

The calculated Brownian relaxation time was discovered to be ~95 s, which was quite a large value and yielded a peak AMF frequency, *f_P_*, of 121 THz for the maximum heat dissipation or movement of the MSP filler. The large values were attributed to the large particle size. Thus, the maximum rotational movement could not be achieved as the AMF frequency used in this study was only 330 kHz, coupled with the limitations of the available equipment. Other ways to reduce the peak frequency include increasing the amplitude of the magnetic field. Nonetheless, Mamiya [27] mentions that magnetic torque could easily rotate nanoparticles in the liquid phase at microsecond time scales even though ferromagnetic nanoparticles are large enough for Brownian relaxation to be negligible. In addition, a similar case was conducted wherein the AMF amplitude used was 10 Gauss to induce misalignment of magnetic nanoparticles with *d* = 150 − 300 nm and *l* = 200 nm. It was also noted that a small amplitude magnetic field was enough to control the orientation of magnetic particles [28].

### 3.2. Quantitative Dispersion Analysis of Mixed Matrix Membranes

**Figure 7:**
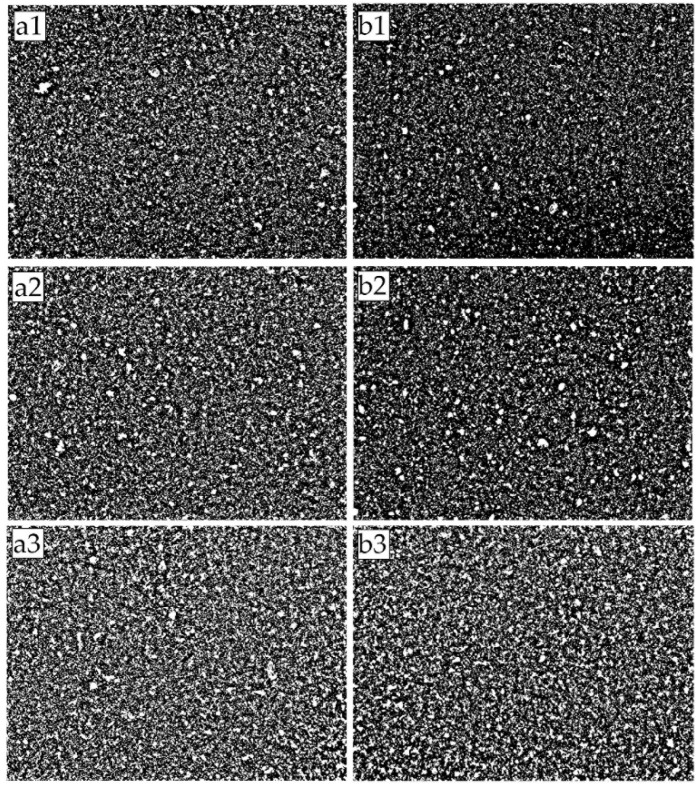
Binarized images of MMM samples: (**a1**) MMM-1; (**b1**) MMM-1 *; (**a2**) MMM-2; (**b2**) MMM-2 *; (**a3**) MMM-3; (**b3**) MMM-3 *; White denotes filler while black denotes polymer phase.

**Figure 8:**
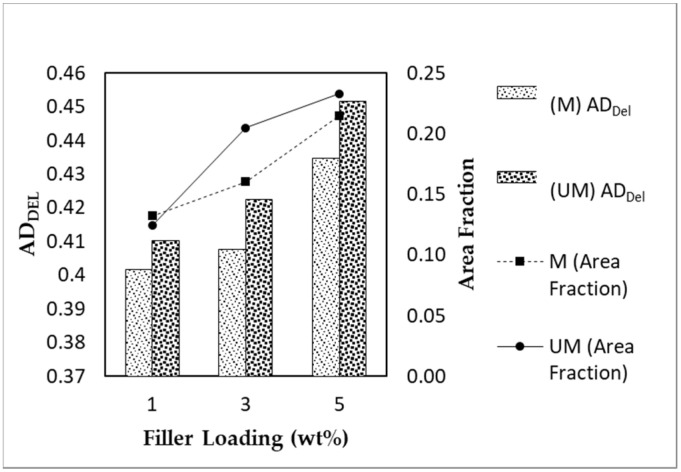
*AD_Del_* and the area fraction of α-Fe_2_O_3_/TiO_2_ MSP fillers in MMMs with varying filler loadings and magnetized conditions (M: Magnetized; UM: Unmagnetized).

**Figure 9:**
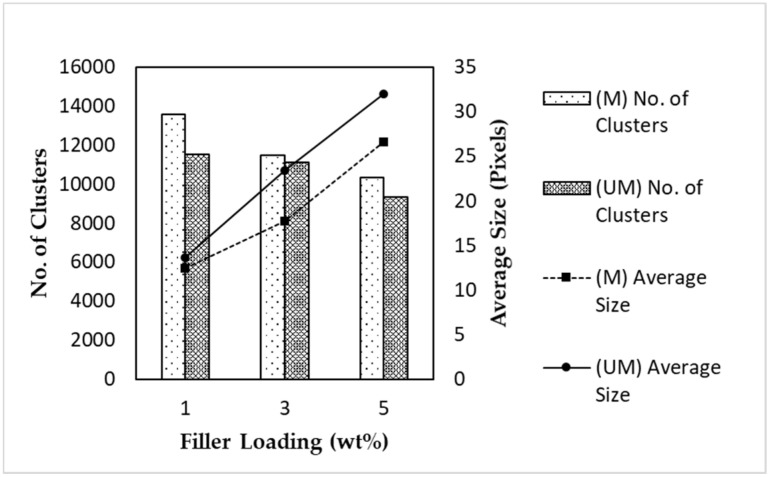
The number of detected clusters and the average size of α-Fe_2_O_3_/TiO_2_ MSPs in MMMs with varying filler loadings and magnetized conditions.

Figure 7 presents the images of the mixed matrix membranes’ surfaces captured using an optical microscope and subsequently binarized using MATLAB to enhance the distinction between the filler and the polymer phase. The white and black represent the filler and the polymer phase, respectively. From the image, it is difficult to discern the quality of dispersion. However, minute differences can be observed as the filler loading increases for both unmagnetized and magnetized MMMs. The images appear to be more saturated with the increment in the filler loading but may not present similar gas separation performances. There were some subtle differences between the images of magnetized and unmagnetized MMM samples for 1 and 3 wt% filler loadings. The magnetized sample, Figure 7b1,b2, has a slightly higher black color intensity (polymer phase) than the unmagnetized sample. The finer particles could be attributed to the improved filler dispersion. However, qualitative observation differs from person to person and may result in an inaccurate interpretation. Hence, the dispersion performance of the filler was quantified using area disorder of Delaunay Triangulation. Upon application of the Delaunay Triangulation network onto the center of mass detected by MATLAB’s in-built function, as described in the methodology section, the quantitative dispersion was then computed and is presented in Table 2. The particles detected are mainly a cluster of particles or agglomerates. The clustered particles are registered as a single entity and only contained a single center of mass. The images were taken under similar lighting conditions, and the dispersion results were computed using the same parameters to reduce biases. Figure 8 and Figure 9 display the values in Table 2 in graphical format for clarity.

The area disorder of Delaunay Triangulation (*AD_Del_*) is a dimensionless quantity used to measure the extent of dispersion. *AD_Del_* measures the global regularity of the particle arrangement within a system (two-dimensional in this study). The value of *AD_Del_* ranges from 0 to 1, with the former representing a perfect lattice-like dispersion of particles and the latter being the most clustered system or worst dispersion. Based on Figure 8, there was a notable trend in which the *AD_Del_* values increased with increments in the filler loading. The dispersion performance of the MSP fillers throughout the sample images becomes worse with regard to the filler loading. Fillers have a higher tendency to agglomerate when their in-between distance decreases due to their high surface area and the strong interaction between fillers [29]. However, there was a clear trend between magnetized and unmagnetized MMMs. The magnetized MMMs had a lower *AD_Del_* value than their unmagnetized counterparts, guiding us towards the notion that the AMF improved the dispersion of MSPs in the polymer phase. In addition, the area fractions of magnetized MSP fillers were also lower compared with the unmagnetized MSP fillers for the filler loadings of 3 and 5 wt%. This trend is in agreement with the *AD_Del_* value measured. We hypothesized that the well-dispersed particles were smaller in size in comparison with clustered fillers. Hence, the smaller particles may be undetectable by the quantification program under the same measurement conditions. There was an anomaly in the area fraction displayed by MMM with 1 wt% filler loading. The relative difference between the area fractions of both MSP fillers with the 1 wt% loading was relatively narrow, suggesting that a minute amount of filler was inadequate to reflect the *AD_Del_* value’s outcome.

Figure 9 depicts the amount and average cluster size in the respective MMMs. These values were detected and computed by the program. An important note is that the program registered agglomerated or clustered particles as a single particle. Hence, this explains the dwindling amount of clusters with increased filler loading, which corroborates the increment in the number of agglomerated particles and the area fraction trends from Figure 8. To further support the *AD_Del_* results, the number of clusters detected in the magnetized MMM was higher than that in the unmagnetized MMM, and the former MMM displayed a lower average cluster number.

**Figure 10:**
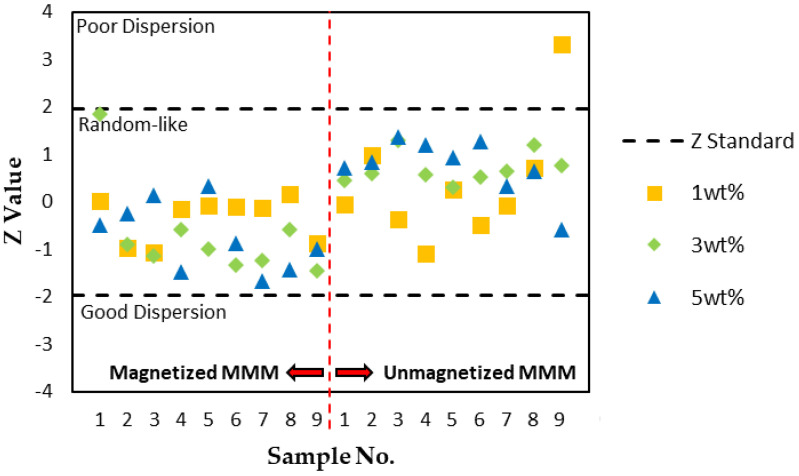
Two-sided Z-test for determination of the filler dispersion performance, *AD_Del_*, and the deviation from the mean behavior of the material.

In conclusion, the magnetization of MMMs via an AMF yielded an improvement in the dispersion performance, supported by a lower occupied area fraction, a higher number of clusters detected, and a smaller average cluster size in the system. To further support the *AD_Del_* results, a statistical two-sided z-test was applied to compare the *AD_Del_* deviation in the measured images from the MMM system’s mean behavior. This step aids in determining whether the measured MMM image is likely to appear from the randomly dispersed MMM after the application of an AMF. The calculated Z values from nine micrographs of each MMM sample are displayed in Figure 10. The measured values from the MMM (magnetized and unmagnetized with the same filler loading) represent the mean behavior of the system.

According to Bray and his team [15,16,17], fluctuations in the particle distribution between samples of a developed hard-core model displayed a Gaussian-distributed pattern in the *AD_Del_* results. If |Z| > 1.96, the null hypothesis is rejected at the 5% significance level. There were three states of dispersion based on the Z-test: poor dispersion, random-like dispersion, and good dispersion. As per Figure 8, the dispersion quality of MSP fillers in MMMs is better than a random-like dispersion when Z < −1.96 and worse when Z > 1.96. A Z value in between signifies that MSP fillers are in a random-like state. It was also assumed that the filler is spherical, and the aspect ratio was not considered in the measurement of Z values. Based on Figure 10, the dispersion quality of all of the fillers was random-like, signifying a lack of deviation from the random images sampled. Only one micrograph from one MMM sample was statistically significant and exhibited poorer dispersion behavior relative to the system; one out of nine samples from the unmagnetized sample with a 1 wt% filler loading. According to the reference model, the rest of the *AD_Del_* results were statistically insignificant as the majority of the samples were indeterminately random. Most of the null hypotheses failed to be rejected. This could be due to inadequate micrograph samples, insignificant statistical results, or considerable variation in the number of particles between samples of the same material. Bray also mentioned that the fluctuation in particle numbers between images does not account for the Z value, which this study suffers from. We also infer that this study’s low magnetic field strength could also have accounted for the insignificant results. The raw data from the dispersion measurements are located in Appendix A, Table A1, Table A2 and Table A3. Though the results were proven to be statistically insignificant, most of the magnetized MMM samples with 3 and 5 wt% filler loadings had lower Z values (Z < 0) in comparison with unmagnetized MMMs. As for MMMs with a 1 wt% filler loading, both magnetized and unmagnetized MMM samples had similar Z values, denoting little effect on the dispersion of the fillers by the AMF.

### 3.3. Gas Separation Performance of Mixed Matrix Membranes

**Figure 11:**
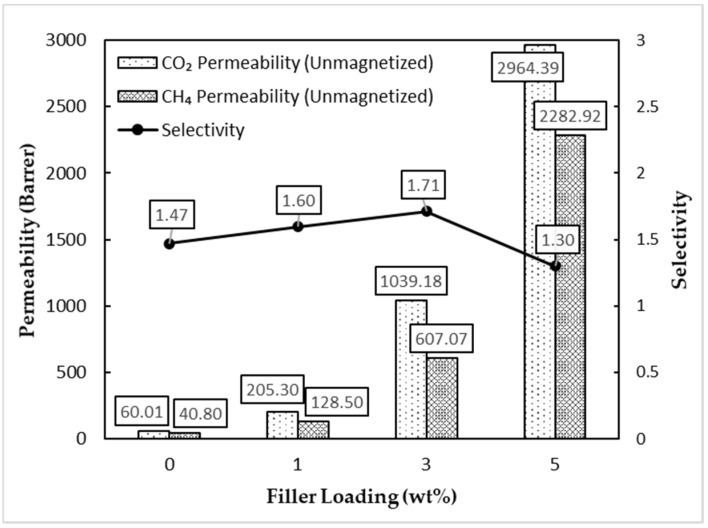
Gas separation performance of unmagnetized MMMs.

Based on Figure 11, the permeability of both CO_2_ and CH_4_ gases increased with an increment in the filler loading for all three unmagnetized MMMs. It can be seen that the selectivity reached a maximum of 1.71 at the 3 wt% filler loading. Further addition of MSP filler leads to a reduction in CO_2_/CH_4_ gas selectivity. The addition of nano-filler was said to break the crystallized polymer chain’s alignment and subsequently increase the total fractional free volume (FFV) of the polymer. The presence of hydroxyl (-OH) functional groups on the surface of TiO_2_ also contributes to more significant interaction with polar CO_2_ molecules in comparison with CH_4_ gas, thereby facilitating the increment in CO_2_ gas permeabilities [30,31]. However, our filler was in the submicron range, and we hypothesize the effect to be similar. However, clusters and agglomerated fillers tend to form, according to measurements from Figure 9, upon an increment in filler loading. Hence, this would lead to a decline in gas separation performance [32]. On a side note, α-Fe_2_O_3_ also has active sites on its surface that can react with CO_2_ gas molecules to form iron carbonate. The increase in the number of active sites may explain the increase in the CO_2_ gas permeation [33]. The pristine polymer membrane acted as a control and was expected to have a lower gas separation performance than the MMM.

**Figure 12:**
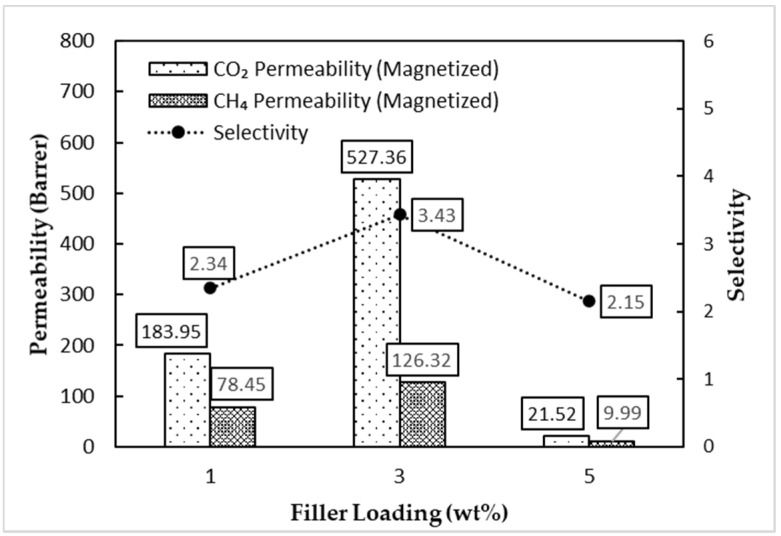
Gas separation performance of the magnetized MMM.

Figure 12 shows that the magnetized MMM performed better in CO_2_/CH_4_ gas selectivity for all three MMMs with a similar filler loading compared with the unmagnetized MMM. The magnetized MMM had a selectivity increase of 46%, 100%, and 65% for the 1, 3, and 5 wt% filler loadings compared with the unmagnetized MMM, respectively. The selectivity trend concurs with the quantitative dispersion value, *AD_Del_*, from the previous section and provides more substantial evidence on the correlation between gas selectivity and the dispersion of the filler. The magnetized MMM had a lower *AD_Del_* value and greater gas selectivity than the unmagnetized MMM for all three filler loadings. However, there was a drastic drop regarding the permeability of the 3 and 5 wt% filler loadings in the magnetized MMM. As for the 1 wt% filler loading, there was only a slight drop, which may be attributed to the insignificant effects of the AMF towards the dispersion of the filler with a lower count. The magnetized MMM had a CO_2_ permeability reduction of 11%, 97%, and 3472% for the 1, 3, and 5 wt% filler loadings compared with the unmagnetized MMM, respectively.

Nevertheless, the permeability–selectivity trade-off exhibited is common and emerges in virtually all synthetic polymer membranes. Enhancement of selectivity is typically accompanied by a reduction in permeability, as explained by the Robeson Trade-off Curve [34]. This phenomenon is attributed to the inability of the membrane to increase its free-volume element size while narrowing the free-volume element size distribution. The increase in the free-volume element size of the membrane allows for a greater permeability of the gas, while the latter increases the gas selectivity [35]. In this case, a greater presence of MSP clusters in the unmagnetized MMM may cause more significant polymer chain packing disruptions and more interfacial voids than the relatively finer MSP fillers in magnetized MMMs. The increase in fractional free volume resulted in a higher relative gas permeability in the unmagnetized MMM. Raveshiyan, et al. [36] have carried out a study wherein iron oxide filler of 50 nm in diameter resulted in greater O_2_ gas permeability relative to a similar filler of 20 nm in diameter in the case of O_2_/N_2_ gas separation. Since the cluster size and dispersion were relatively finer and better in the magnetized MMM, there may be enhanced rigidity between the polymer–filler interface. The rigidity then contributes to increased tortuosity and hinders the transport of gas molecules, resulting in increased selectivity [37]. Hence, it was inferred that the improved dispersion of fillers reduced the free-volume element and narrowed the free-volume size distribution.

When the filler loading in the magnetized MMM was increased from 3 to 5 wt%, the permeability of the CO_2_ and CH_4_ gases decreased drastically. This trend was contradictory to the results shown by the unmagnetized MMM. In the previous section, the *AD_Del_* value from the two-sided z-test showed a shift in the dispersion state after magnetization from poor dispersion to random dispersion. The improved dispersion signifies a relatively better distribution of filler throughout the MMM. The reduction in permeability could then stem from a reduction in the relative free volume or increased tortuosity in the gas diffusion pathway. Uniformly dispersed fillers disrupt the polymer chain packing, which induces excess free volume and improved gas permeabilities [38]. However, the dispersion may have also increased the rigidity of the polymer–filler interaction and reduced interfacial voids, which leads to a reduction in permeability.

To conclude, we infer that there was a correlation between filler dispersion and gas separation performance. The results show that the overall selectivity of the magnetized MMM improved but suffered from a permeability trade-off. It was not determined whether the AMF magnetic field was partly responsible for this incident or could be largely dependent on the properties of the filler itself as this trade-off is a common occurrence in synthetic polymer membranes.

### 3.4. Limitations and Future Directions

This study has shown promising results on the application of AMFs towards filler dispersion. However, the proof-of-concept can be further strengthened by other quantitative assessments to attest to the dispersion results presented in this study. Filler particles can be further tailored towards reducing their size while ensuring that the Brownian relaxation time dominates the relaxation mechanism and enhancement of their magnetic properties. This may lead to improved filler dispersion via AMFs due to magnetic fillers having a lower Brownian relaxation time and an increased response towards the magnetic field. Due to the limitations of the equipment, the experiment was only conducted at a low AMF amplitude. A higher AMF amplitude could be employed in the future. Apart from AMFs, the rotational magnetic field direction (RMF) is also currently utilized to induce the rotation and movement of magnetic particles. The RMF could be compared with AMFs for the dispersion of magnetic fillers in MMMs. However, we did not do it in this study due to the limitations of the equipment. The RMF requires twice the amount of equipment in comparison with AMFs.

## Figures and Tables

**Table 1:** MMM filler compositions and state of magnetization.

Sample	Filler wt%	Magnetic Field
MMM-0	0	Absent
MMM-1	1	Absent
MMM-3	3	Absent
MMM-5	5	Absent
MMM-1 *	1	Present
MMM-3 *	3	Present
MMM-5 *	5	Present

**Table 2:** Quantitative dispersion performance of α-Fe_2_O_3_/TiO_2_ MSPs in MMMs.

Sample	*AD_Del_*	Area Fraction	No. of Clusters	Average Size (Pixels)
MMM-1	0.41	0.12	11,513.00	13.67
MMM-3	0.42	0.20	11,122.00	23.43
MMM-5	0.45	0.23	9321.00	31.94
MMM-1 *	0.40	0.13	13,584.00	12.40
MMM-3 *	0.41	0.16	11,494.00	17.76
MMM-5 *	0.43	0.21	10,340.00	26.54

## Supplementary Materials

The following are available online at https://www.mdpi.com/article/10.3390/membranes11080641/s1, Figures S1a–S54a: Optical Microscope images of MMMs; Figures S1b–S54b: Binarized OM Images.

## Appendix A

**Table A1:** Dispersion measurements of MMMs with a 1 wt% filler loading.

MMM Sample	Micrograph No.	*AD_Del_*	Cluster No.	Area Fraction	Average Size (Pixels)	Z Value
MMM-1	1	0.406	13,329	0.130	12.435	0.029
MMM-1	2	0.394	13,802	0.132	12.213	−0.967
MMM-1	3	0.393	14,280	0.133	11.809	−1.061
MMM-1	4	0.404	13,321	0.132	12.621	−0.153
MMM-1	5	0.405	13,273	0.134	12.889	−0.072
MMM-1	6	0.405	13,361	0.132	12.573	−0.106
MMM-1	7	0.404	13,427	0.132	12.464	−0.134
MMM-1	8	0.408	13,134	0.131	12.716	0.166
MMM-1	9	0.396	14,333	0.134	11.921	−0.869
Mean of MMM-1		0.402	13,584.44	0.132	12.405	N/A
Std Deviation of MMM-1		0.006	447.3159	0.001	0.360	N/A
MMM-1 *	1	0.405	11,508	0.115	12.758	−0.049
MMM-1 *	2	0.418	11,080	0.122	13.993	0.975
MMM-1 *	3	0.401	12,269	0.183	18.989	−0.380
MMM-1 *	4	0.393	12,412	0.139	14.215	−1.080
MMM-1 *	5	0.409	11,625	0.114	12.517	0.247
MMM-1 *	6	0.400	11,657	0.124	13.555	−0.489
MMM-1 *	7	0.405	11,644	0.118	12.935	−0.082
MMM-1 *	8	0.414	11,084	0.113	12.951	0.706
MMM-1 *	9	0.446	10,338	0.090	11.090	3.321
Mean of MMM-1 *		0.410	11,513	0.124	13.667	N/A
Std Deviation of MMM-1 *		0.015	630.7097	0.025	2.196	N/A
Mean for 1 wt% filler loading		0.406	N/A	N/A	N/A	N/A
Std Deviation for 1 wt% filler loading		0.012	N/A	N/A	N/A	N/A

**Table A2:** Dispersion measurements of MMMs with a 3 wt% filler loading.

MMM Sample	Micrograph No.	*AD_Del_*	Cluster No.	Area Fraction	Average Size (Pixels)	Z Value
MMM-3	1	0.434	10,799	0.160	18.880	2.542
MMM-3	2	0.406	11,125	0.160	18.323	−0.188
MMM-3	3	0.403	11,639	0.157	17.176	−0.437
MMM-3	4	0.409	11,522	0.164	18.083	0.115
MMM-3	5	0.405	11,571	0.160	17.616	−0.283
MMM-3	6	0.401	11,816	0.158	17.013	−0.632
MMM-3	7	0.402	11,671	0.161	17.502	−0.515
MMM-3	8	0.409	11,535	0.160	17.637	0.131
MMM-3	9	0.400	11,771	0.163	17.603	−0.733
Mean of MMM-3		0.408	11,494.33	0.160	17.759	N/A
Std Deviation of MMM-3		0.011	327.9379	0.002	0.581	N/A
MMM-3 *	1	0.420	11,311	0.204	22.973	−0.737
MMM-3 *	2	0.421	11,495	0.206	22.813	−0.323
MMM-3 *	3	0.429	10,972	0.204	23.667	1.774
MMM-3 *	4	0.421	10,957	0.208	24.145	−0.419
MMM-3 *	5	0.418	11,265	0.205	23.158	−1.215
MMM-3 *	6	0.421	11,267	0.204	22.995	−0.533
MMM-3 *	7	0.422	11,076	0.204	23.398	−0.197
MMM-3 *	8	0.428	10,915	0.202	23.603	1.484
MMM-3 *	9	0.423	10,840	0.206	24.137	0.166
Mean of MMM-3 *		0.422	11,122	0.205	23.432	N/A
Std Deviation of MMM-3 *		0.004	220.9463	0.002	0.494	N/A
Mean for 3 wt% filler loading		0.415	N/A	N/A	N/A	N/A
Std Deviation for 3 wt% filler loading		0.011	N/A	N/A	N/A	N/A

**Table A3:** Dispersion measurements of MMMs with a 5 wt% filler loading.

MMM Sample	Micrograph No.	*AD_Del_*	Cluster No.	Area Fraction	Average Size (Pixels)	Z Value
MMM-5	1	0.438	10,043	0.216	27.385	−0.483
MMM-5	2	0.440	9482	0.217	29.184	−0.258
MMM-5	3	0.445	10,021	0.217	27.545	0.144
MMM-5	4	0.426	11,107	0.215	24.579	−1.475
MMM-5	5	0.447	9345	0.220	29.932	0.337
MMM-5	6	0.433	10,744	0.216	25.595	−0.866
MMM-5	7	0.424	10,942	0.210	24.371	−1.662
MMM-5	8	0.427	10,645	0.210	25.110	−1.431
MMM-5	9	0.432	10,738	0.212	25.169	−0.993
Mean of MMM-5		0.435	10,340.77778	0.215	26.541	N/A
Std Deviation of MMM-5		0.008	640.4579958	0.003	2.049	N/A
MMM-5 *	1	0.451	8914	0.236	33.680	0.721
MMM-5 *	2	0.453	9011	0.238	33.584	0.838
MMM-5 *	3	0.459	8809	0.240	34.609	1.362
MMM-5 *	4	0.457	9095	0.233	32.583	1.187
MMM-5 *	5	0.454	9102	0.236	33.023	0.937
MMM-5 *	6	0.458	9003	0.233	32.888	1.268
MMM-5 *	7	0.447	10,217	0.225	27.967	0.323
MMM-5 *	8	0.451	9539	0.233	31.146	0.645
MMM-5 *	9	0.436	10,206	0.224	27.990	−0.595
Mean of MMM-5 *		0.452	9321.777778	0.233	31.941	N/A
Std Deviation of MMM-5 *		0.007	542.8643426	0.005	2.433	N/A
Mean for 3 wt% filler loading		0.443	N/A	N/A	N/A	N/A
Std Deviation for 3 wt% filler loading		0.426	N/A	N/A	N/A	N/A

## Appendix B

MATLAB Code:

%reading image files

newfolder = dir (“C:\Users\Yap_Y\OneDrive\MSc_Yun Kee\Characterization Results\OM\New folder\”)

imgfile = dir (‘*bw*.png’) %scan file that contains *bw* word

C = cell(1:2)

%Binarinzing image via adaptive thresholding

for kreater presence of MSP clusters in the unmagnetized MMM = 1:1%numel(imgfile) %remove ‘%’ to scan whole file

F = imgfile(k).name

I = imread(F)

G = rgb2gray(I)

ws = 90%window size

convG = imfilter(G,fspecial(‘average’,ws),’replicate’)

C2 = 0.01;

CG = convG-G;

bW = imbinarize (CG, C2)

bW = imcomplement(bW)

%------------------------------------------------------------------------

%Replacing coordinates to simulate boundary condition

%Finding out image row and column element size

[Brow,Bcolumn] = size(bW);

%Simulating column boundary

%prompt = “Please enter column boundary particles=”

%columninterval = input(prompt);

columninterval = 30;

Bcolumninterval = Bcolumn/columninterval;

Bcolumninterval = round(Bcolumninterval);

Bcolumnvalue = 1;

for topcolumn = 1:columninterval

binaryImageB(1,Bcolumnvalue) = 1;

binaryImageB(Brow,Bcolumnvalue) = 1;

Bcolumnvalue = Bcolumnvalue + Bcolumninterval;

if Bcolumnvalue > Bcolumninterval

binaryImageB(1,Bcolumn) = 1;

end

end

%Simulating row boundary

%Finding ratio for row boundary particles

ratio = Bcolumn/Brow;

rowinterval = columninterval/ratio;

rowinterval = round(rowinterval);

Browinterval = Brow/rowinterval;

Browinterval = round(Browinterval);

Browvalue = 1;

for leftrow = 1:rowinterval

binaryImageB(Browvalue,1) = 1;

binaryImageB(Browvalue,Bcolumn) = 1;

Browvalue = Browvalue + Browinterval;

if Browvalue > Browinterval

binaryImageB(Brow,1) = 1;

binaryImageB(Brow,Bcolumn) = 1;

end

end

%-----------------------------------------------------------------------

%Mapping of delaunay network by detecting centre of mass of detected

%particle

props = regionprops(bW,’Centroid’)

areaprops = regionprops(‘table’,bW,’Area’)

xyCentroids = [props.Centroid]

xCentroids = xyCentroids (1:2:end)

yCentroids = xyCentroids (2:2:end)

dots = plot(xCentroids,yCentroids,’r.’,’LineWidth’,1)

tri = delaunay(xCentroids,yCentroids);

triplot(tri,xCentroids,yCentroids,’yellow’)

grid on

figure

imshow(bW,[[])

hold on

triplot(tri,xCentroids,yCentroids)

hold off

areas = polyarea(xCentroids(tri),yCentroids(tri),2);

Omega = mean(areas);

SOmega = std(areas);

ADDel = 1 − (1 + SOmega/Omega)^(−1);

%Area fraction calculation, csaop[cross sectional area of particle]

lambda = numel(areaprops)/numel(bW)

rawcsaop = (areaprops.Area(2:numel(areaprops),:)) %starting from 2 as 1st data has huge outlier

meancsaop = mean(rawcsaop)

Areafrac = lambda*meancsaop

maxarea = max(areaprops.Area(2:numel(areaprops),:))

C{k,1} = F %(numel(imgfile),2)

C{k,2} = ADDel

C{k,3} = numel(areaprops) %number of particles detected/centre of mass

C{k,4} = Areafrac

C{k,5} = maxarea %largest particle size

C{k,6} = meancsaop %average particle size

end
