# Supplementary material for: Effects of an Alternating Magnetic Field towards Dispersion of α-Fe2O3/TiO2 Magnetic Filler in PPOdm Polymer for CO2/CH4 Gas Separation
Source: Membranes (Basel). 2021 Aug 20;11(8):641. doi: 10.3390/membranes11080641 (PMC8401501; doi:10.3390/membranes11080641)
Supplement: Supplementary file 1 [file membranes-11-00641-s001.zip › Supplementary File.pdf]

Supplementary Images

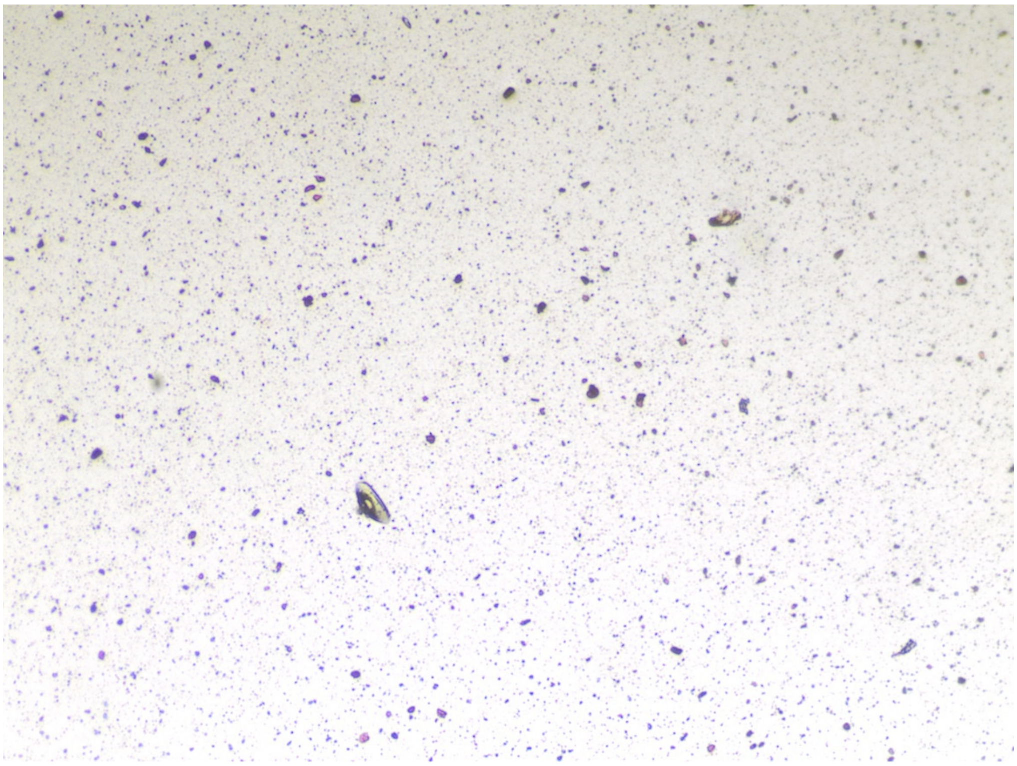

Figure S1a: OM image of MMM-1*, micrograph no.1

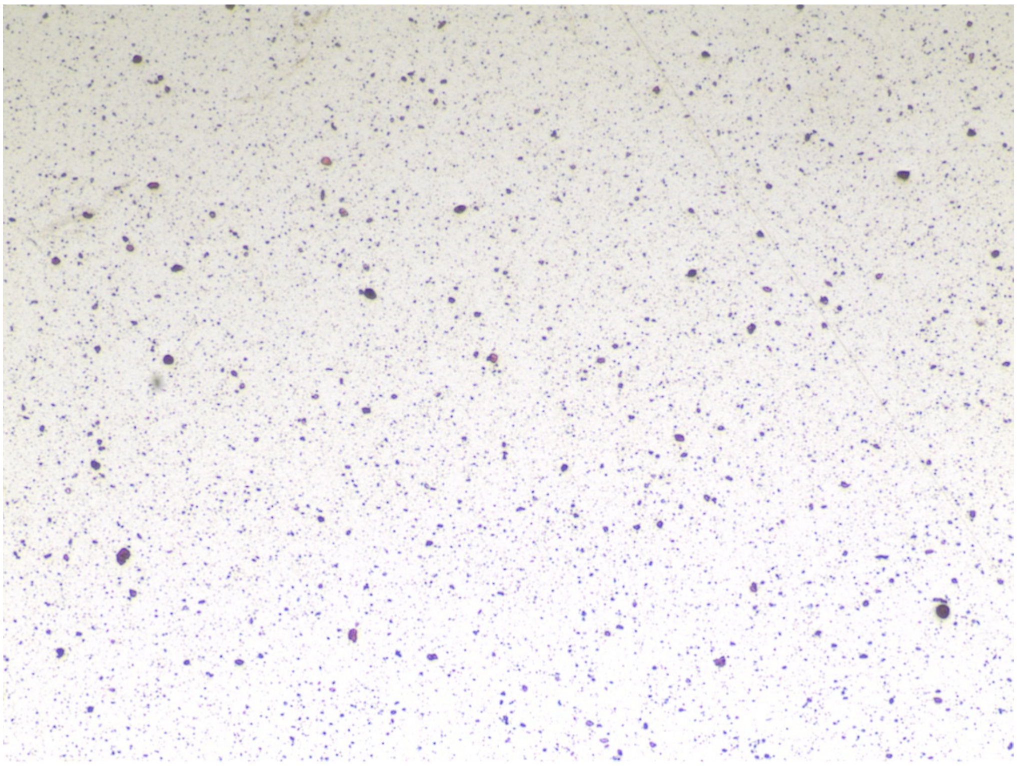

Figure S2a: OM image of MMM-1*, micrograph no.2

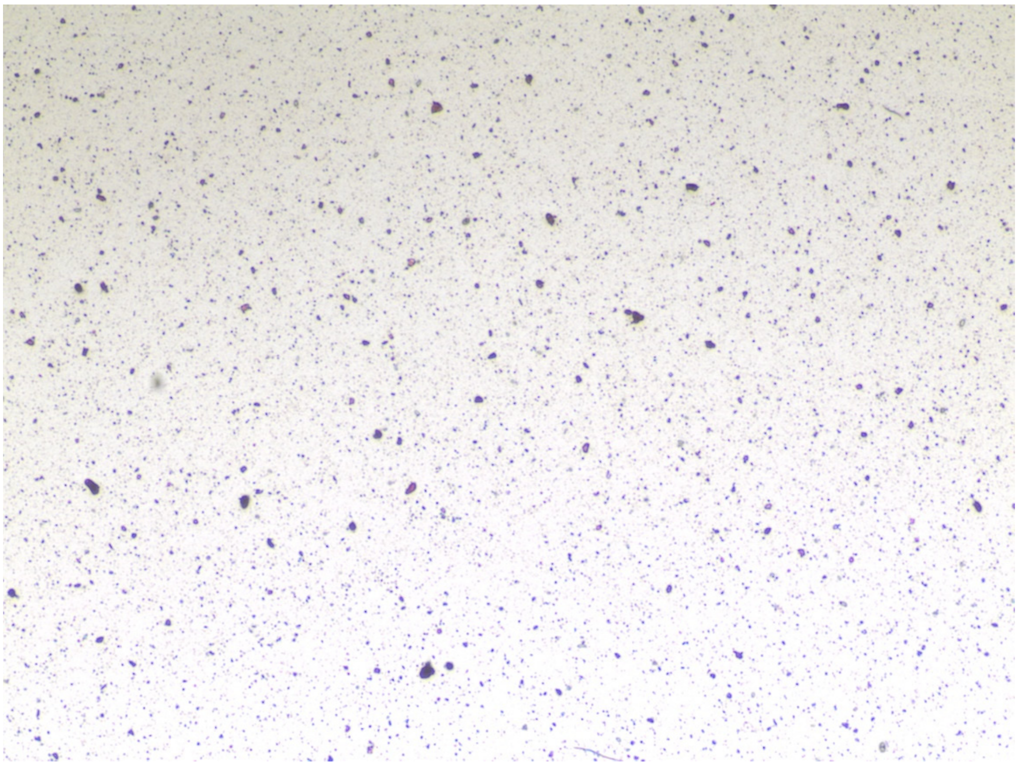

Figure S3a: OM image of MMM-1*, micrograph no.3

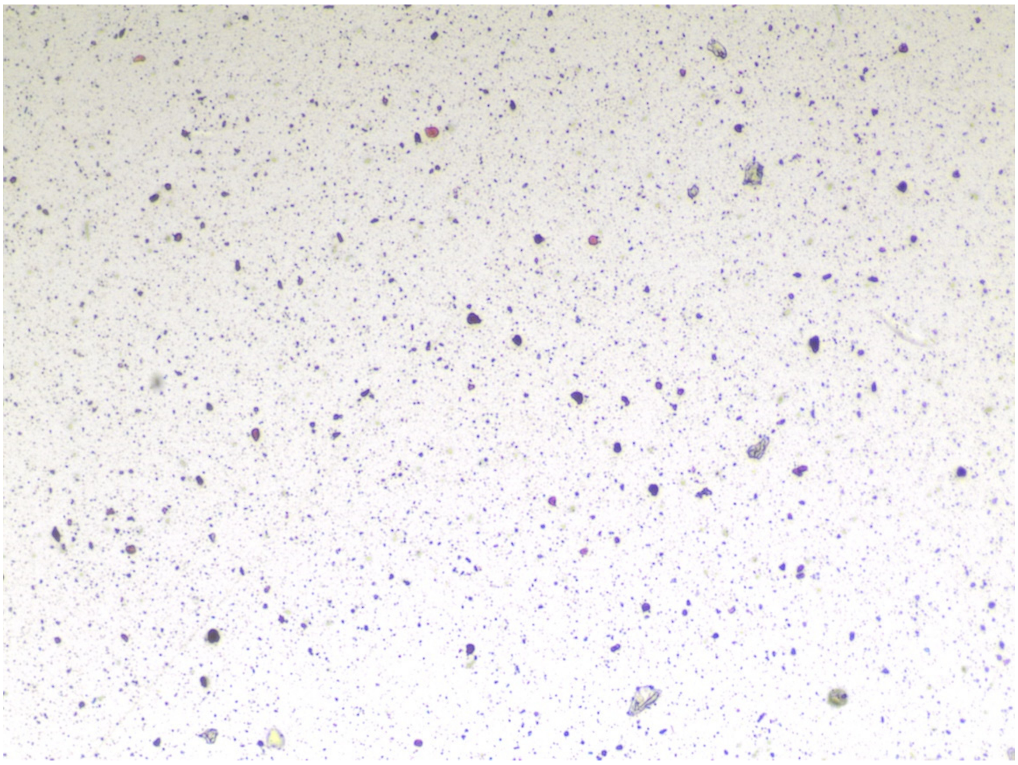

Figure S4a: OM image of MMM-1*, micrograph no.4

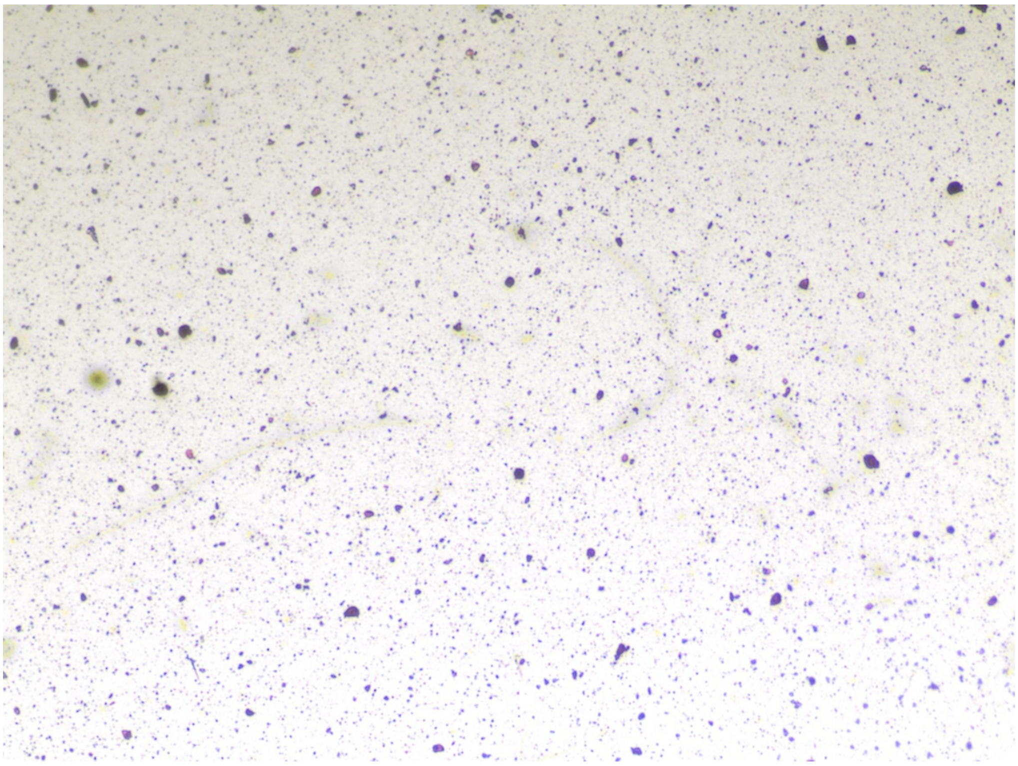

Figure S5a: OM image of MMM-1*, micrograph no.5

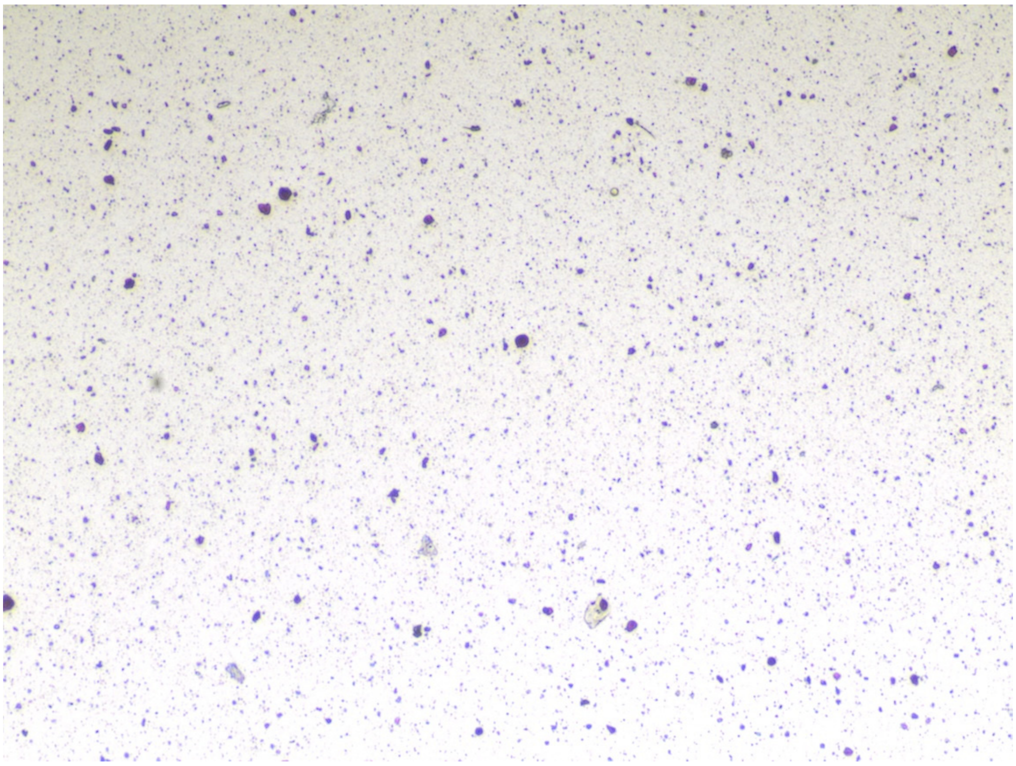

Figure S6a: OM image of MMM-1*, micrograph no.6

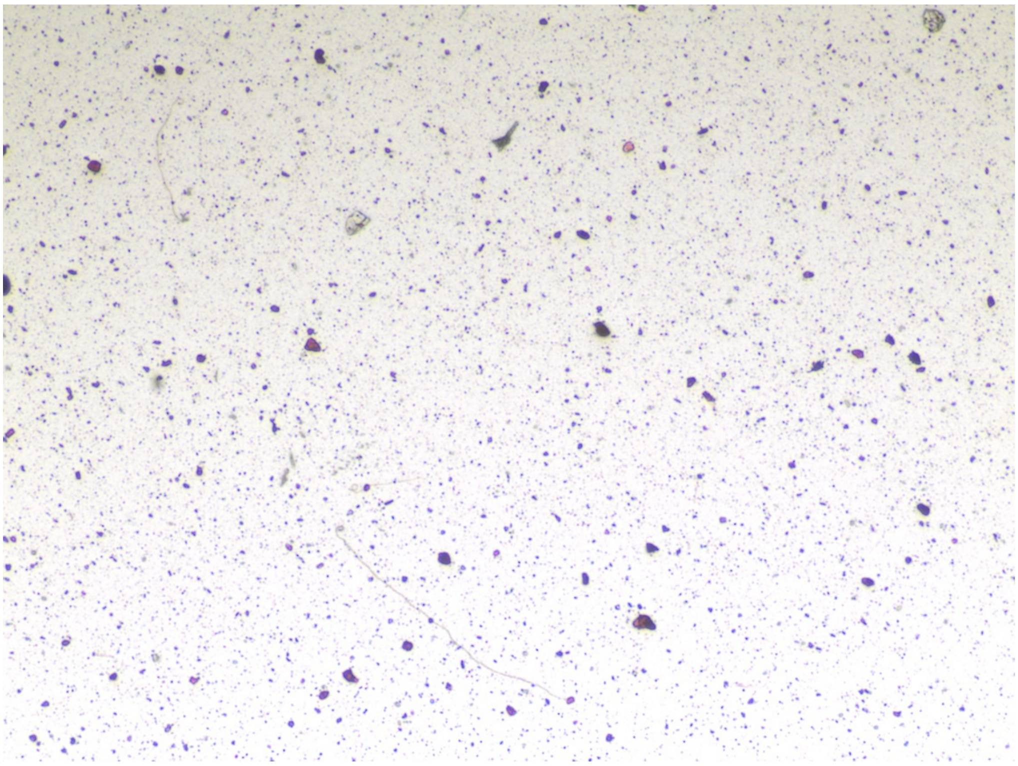

Figure S7a: OM image of MMM-1*, micrograph no.7

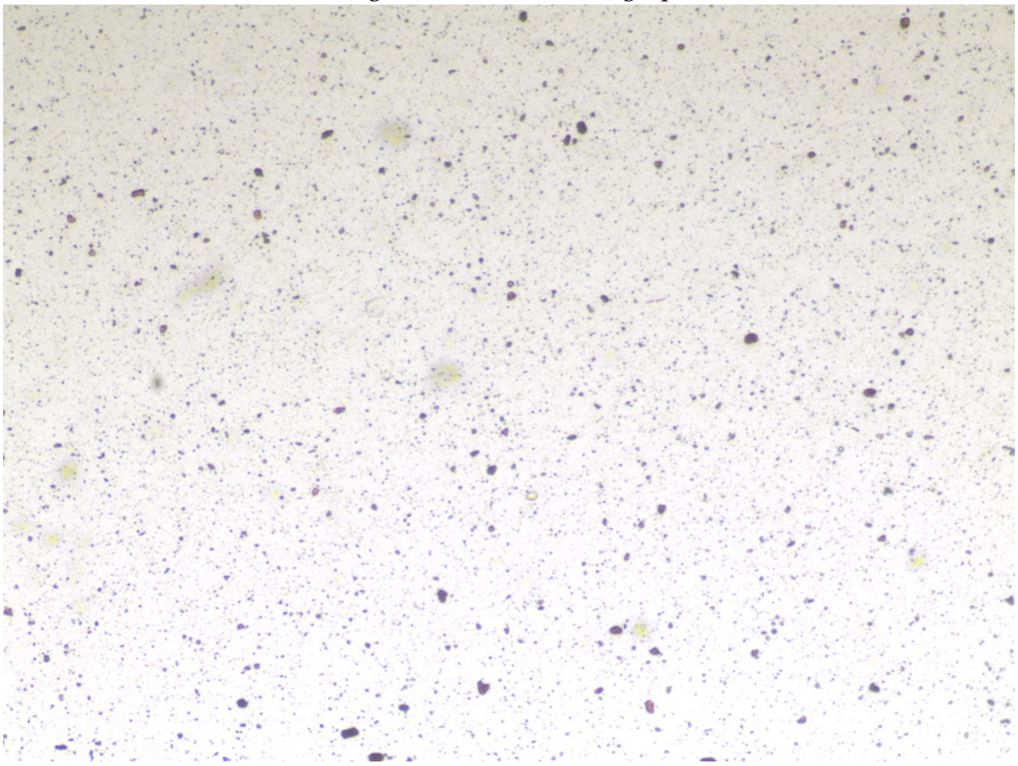

Figure S8a: OM image of MMM-1*, micrograph no.8

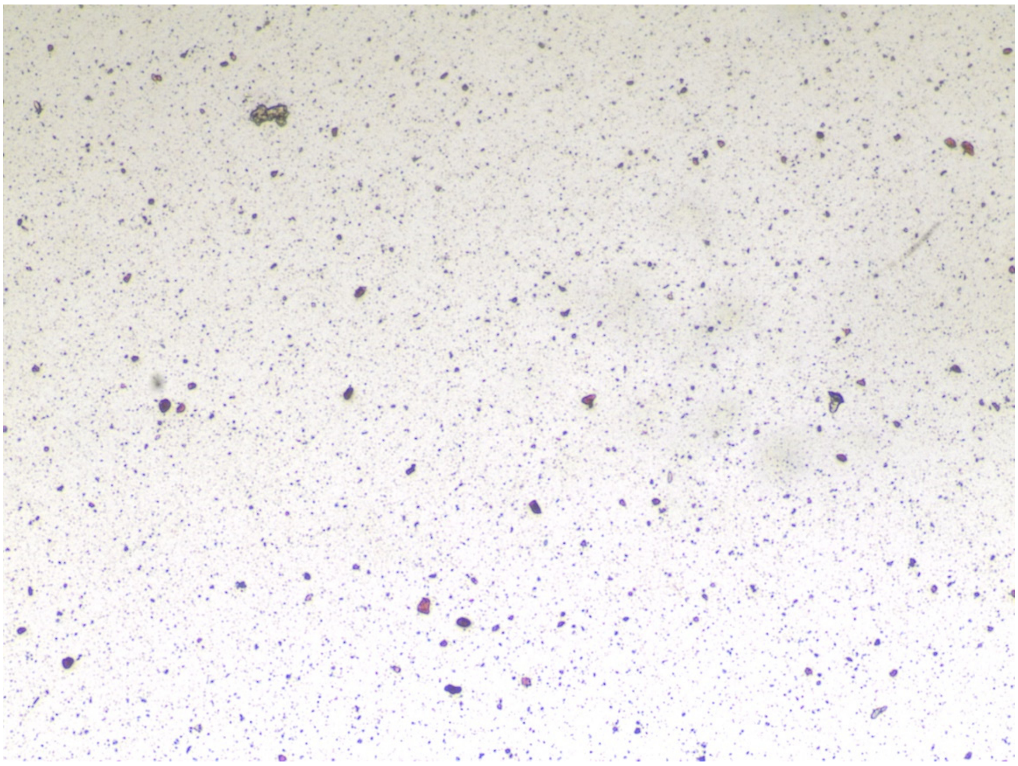

Figure S9a: OM image of MMM-1*, micrograph no.9

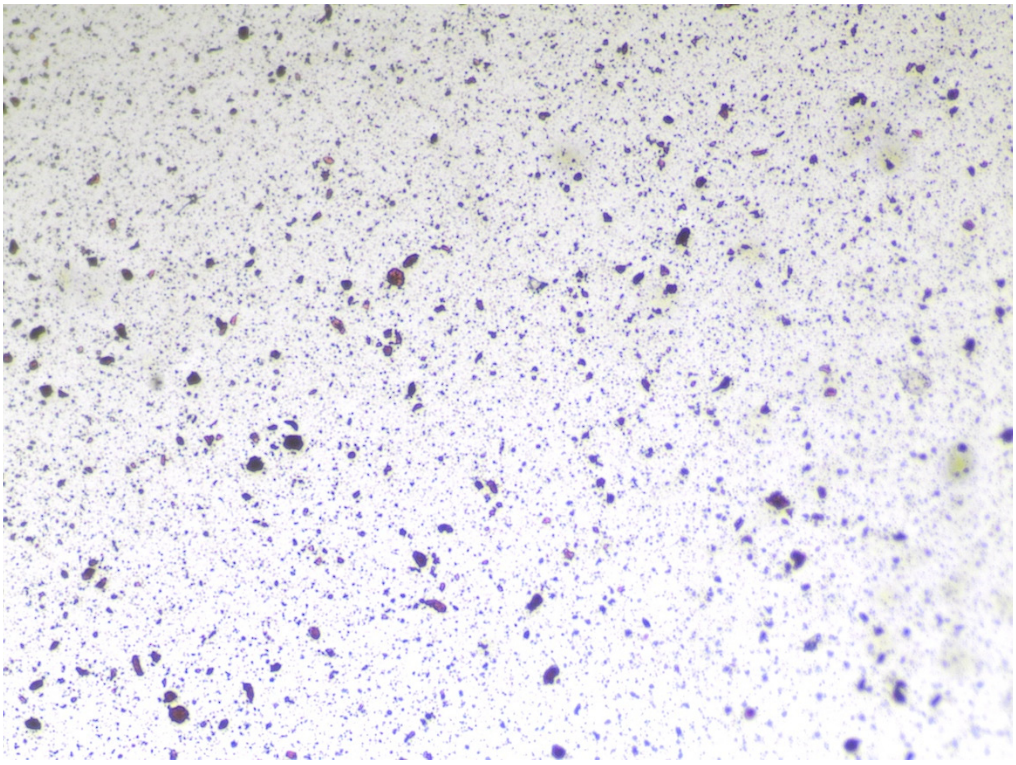

Figure S10a: OM image of MMM-3*, micrograph no.1

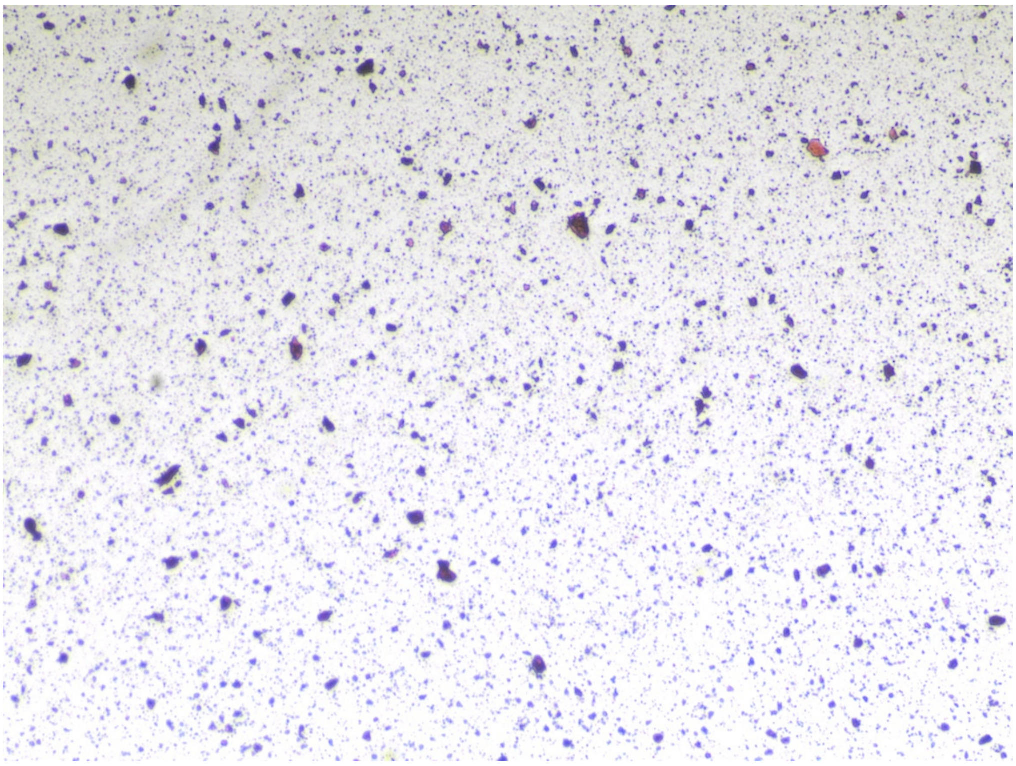

Figure S11a: OM image of MMM-3*, micrograph no.2

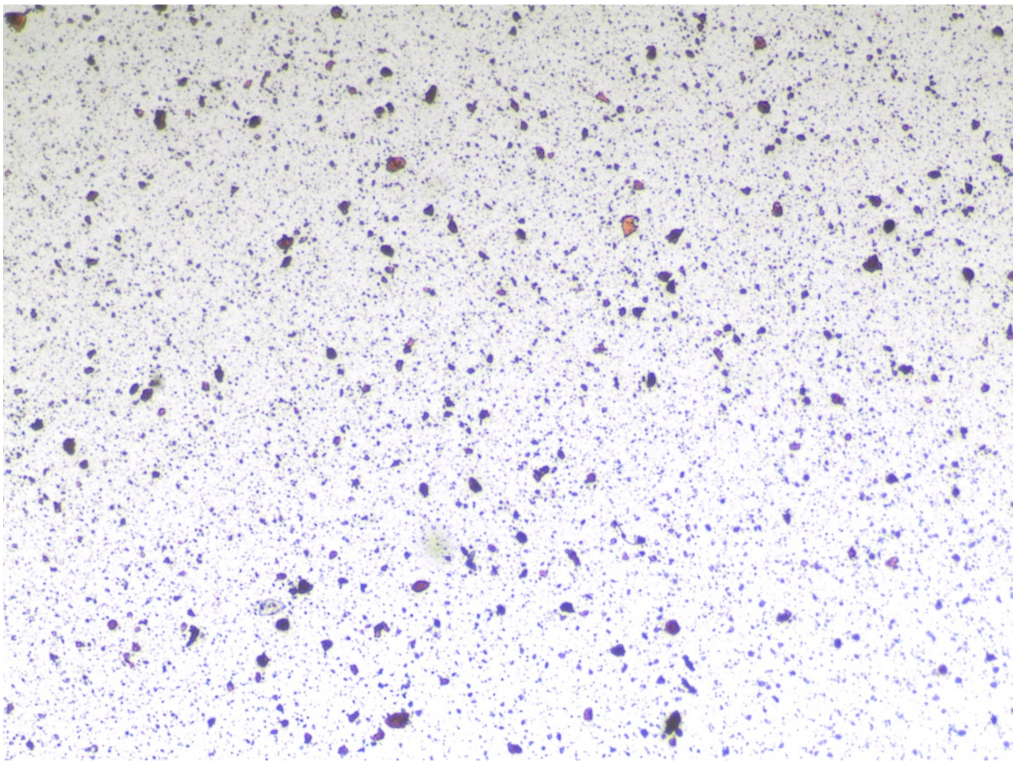

Figure S12a: OM image of MMM-3*, micrograph no.3

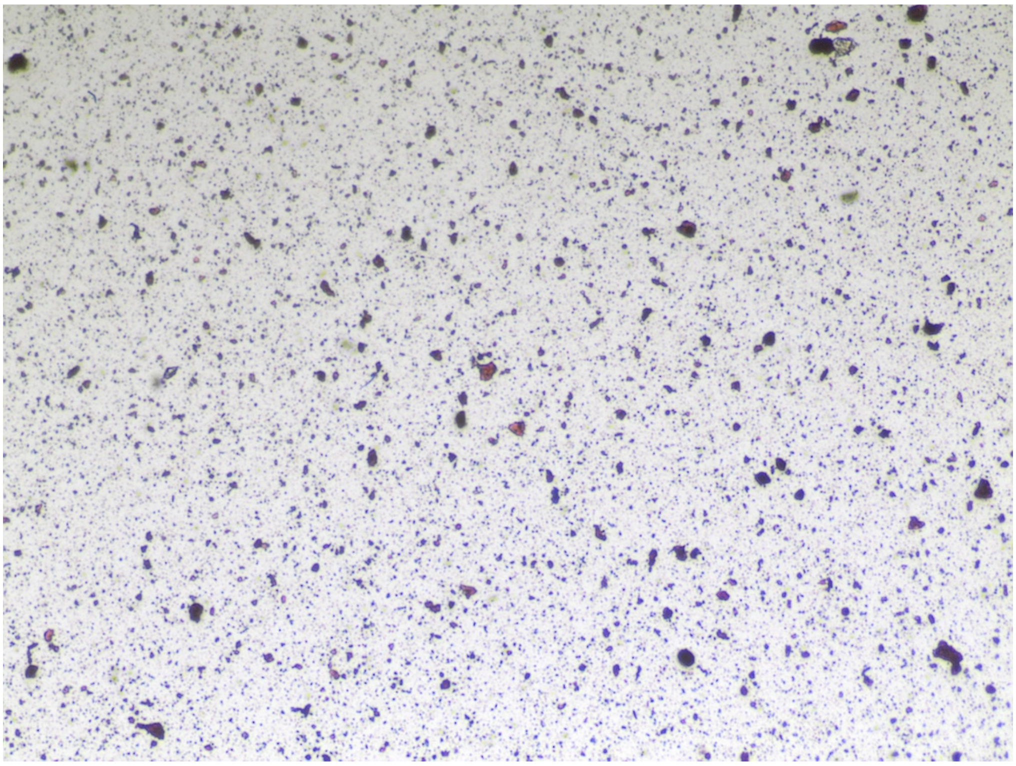

Figure S13a: OM image of MMM-3*, micrograph no.4

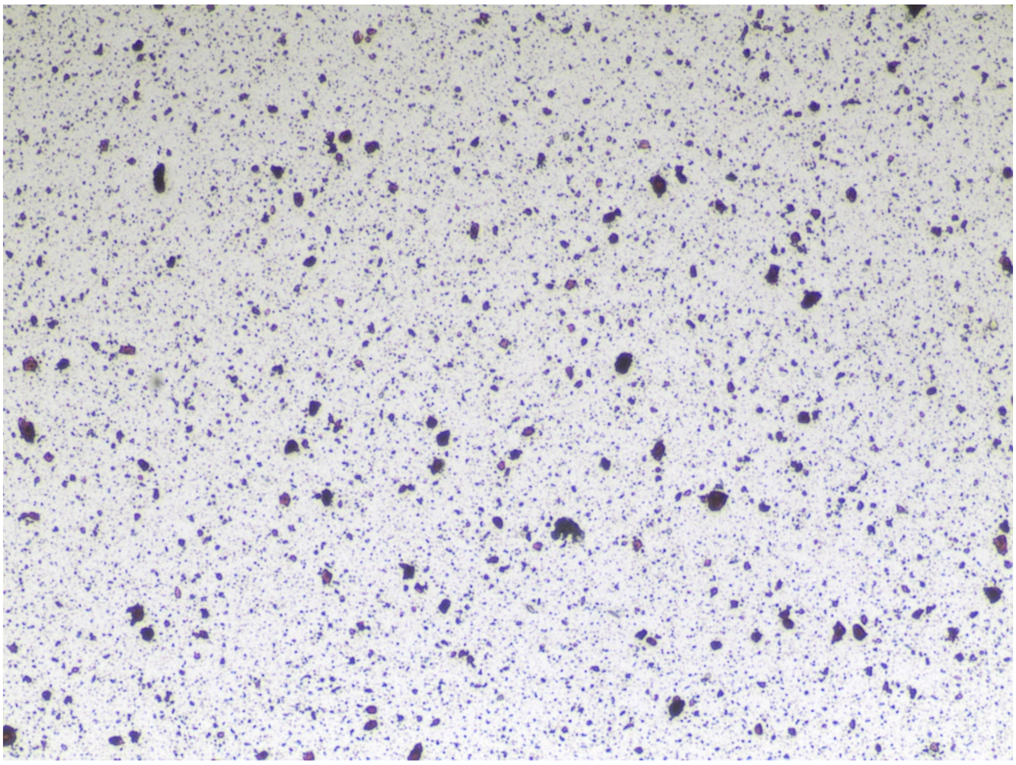

Figure S14a: OM image of MMM-3*, micrograph no.5

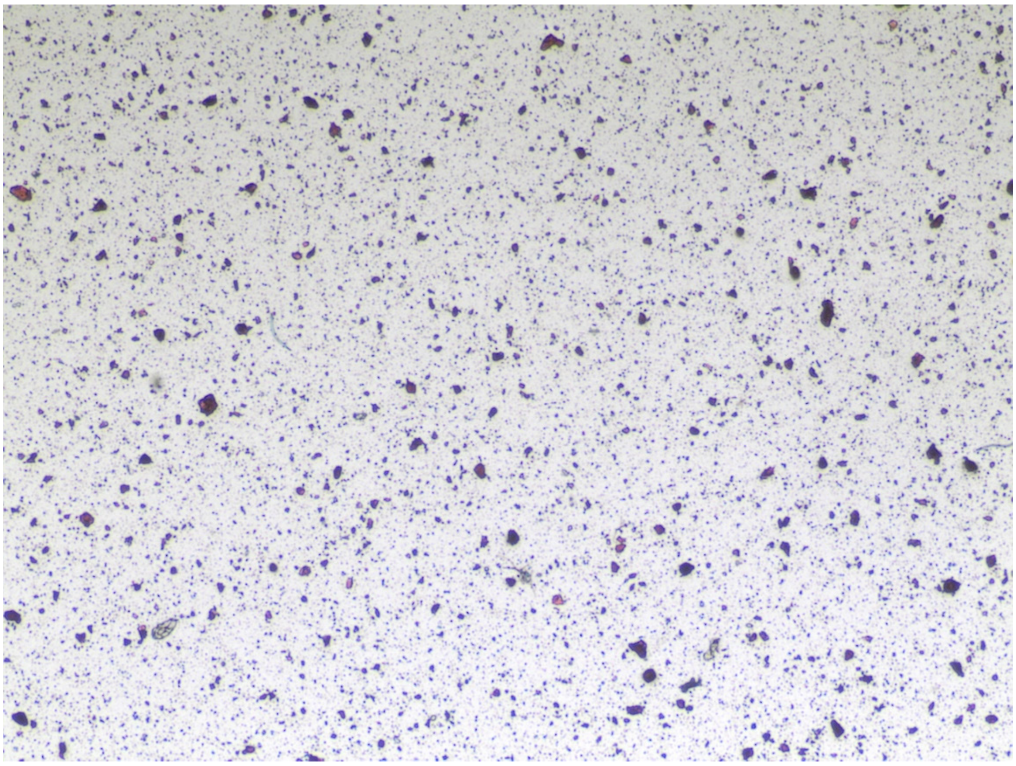

Figure S15a: OM image of MMM-3*, micrograph no.6

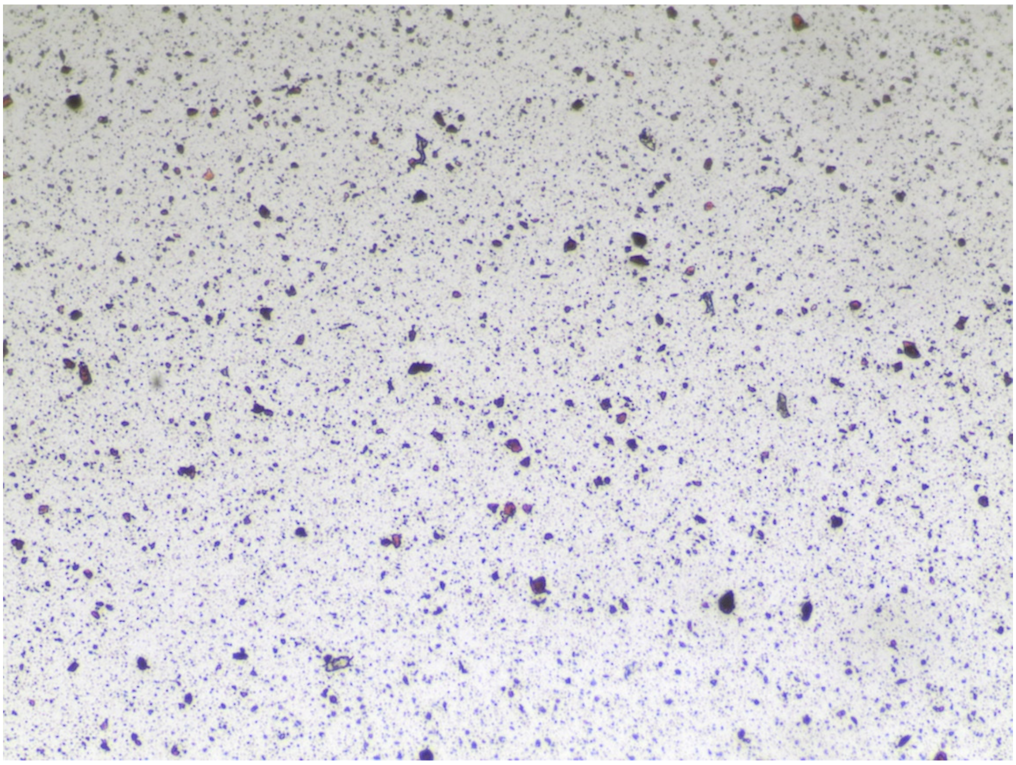

Figure S16a: OM image of MMM-3*, micrograph no.7

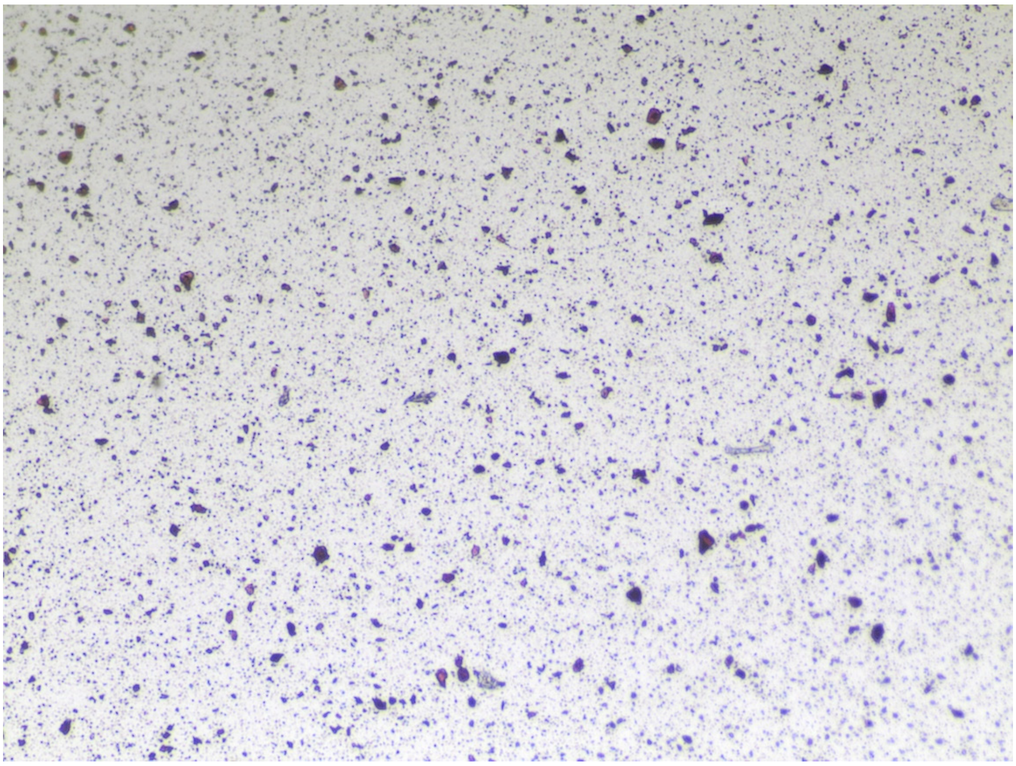

Figure S17a: OM image of MMM-3*, micrograph no.8

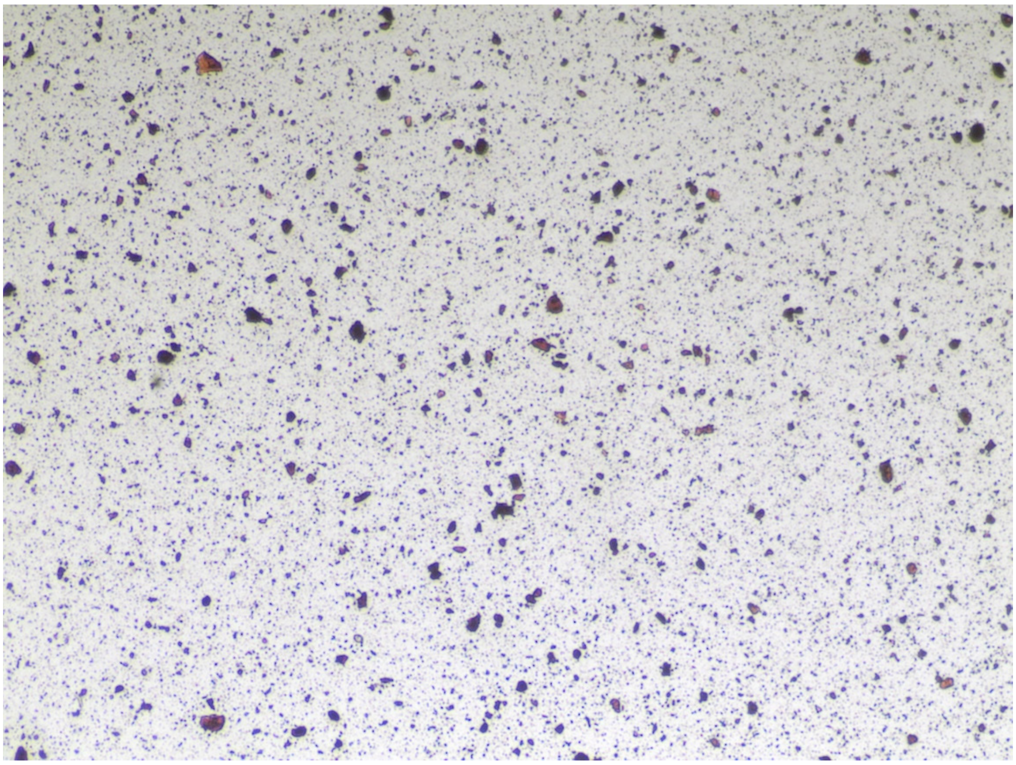

Figure S18a: OM image of MMM-3*, micrograph no.9

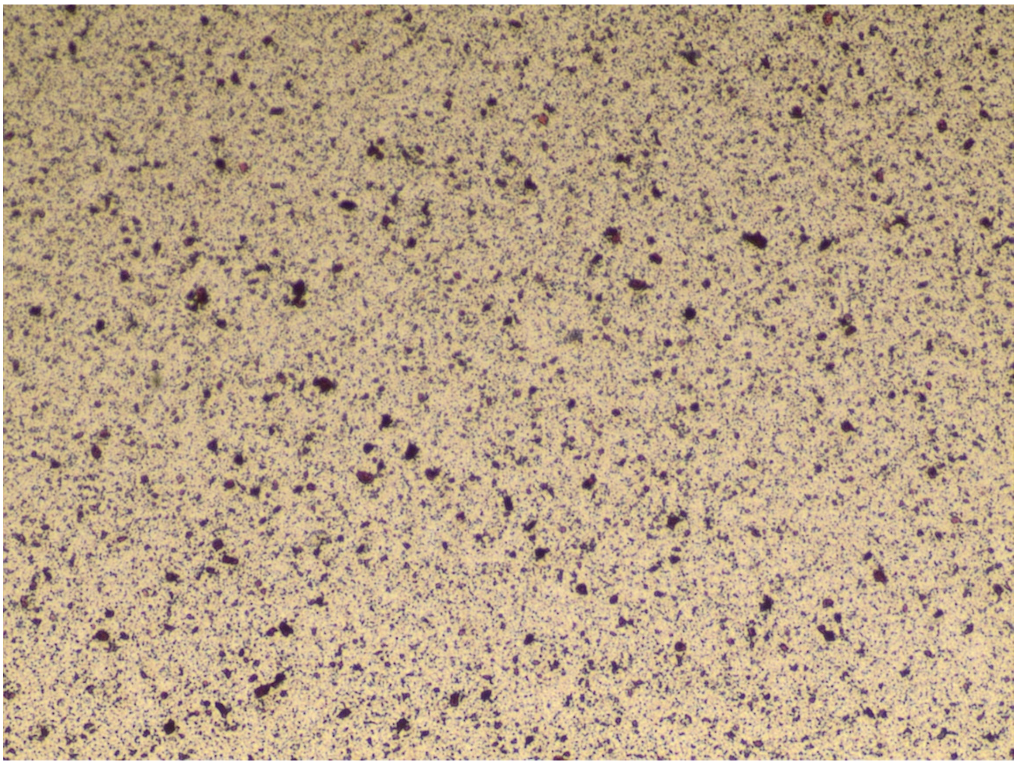

Figure S19a: OM image of MMM-5*, micrograph no.1

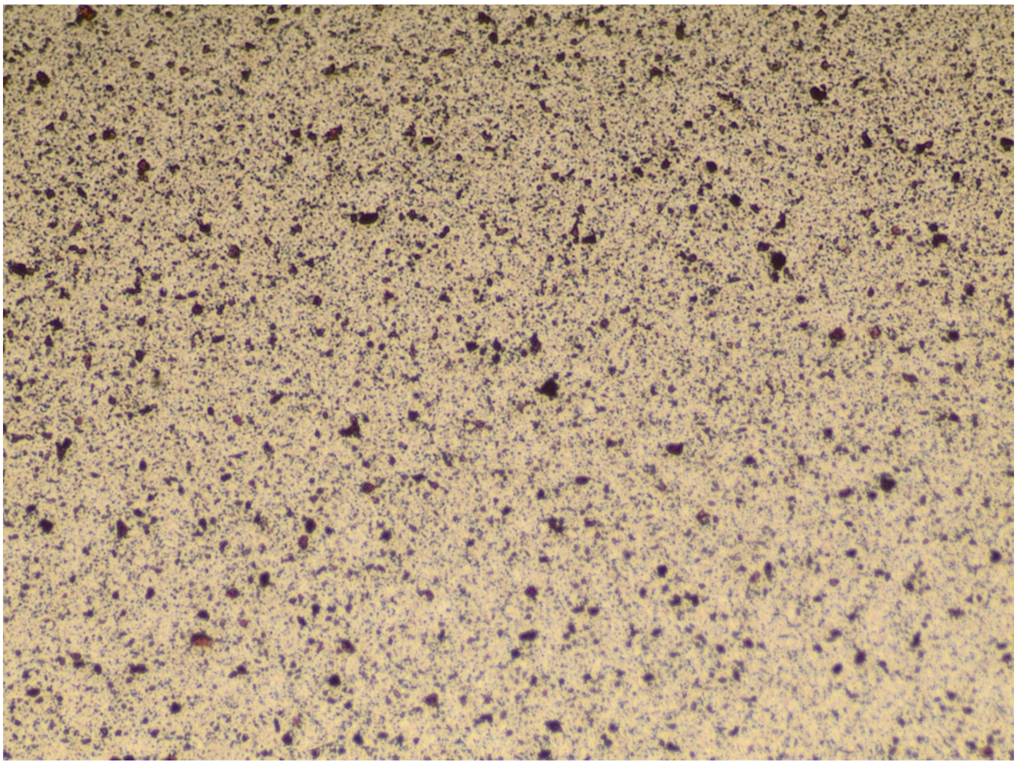

Figure S20a: OM image of MMM-5*, micrograph no.2

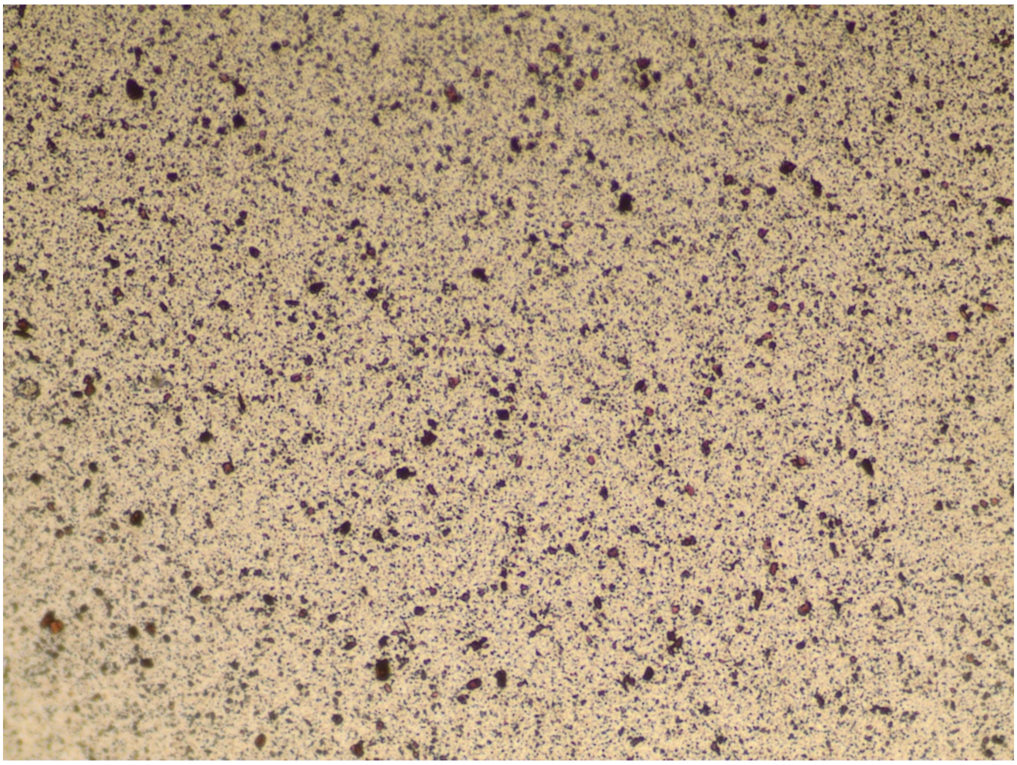

Figure S21a: OM image of MMM-5*, micrograph no.3

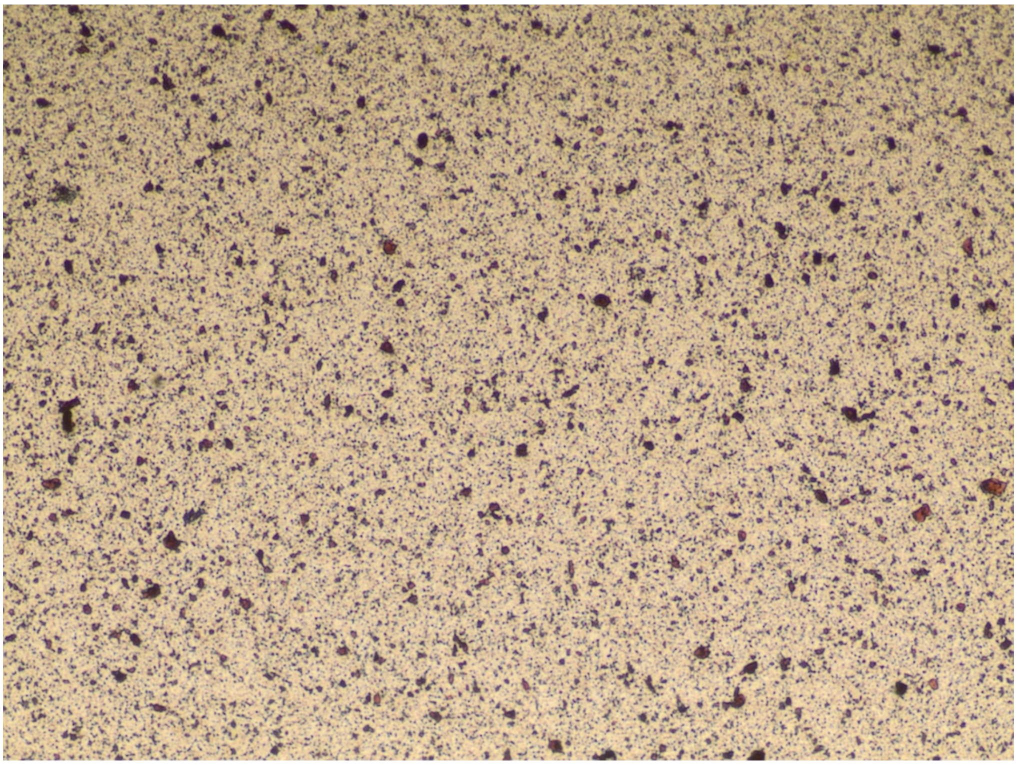

Figure S22a: OM image of MMM-5*, micrograph no.4

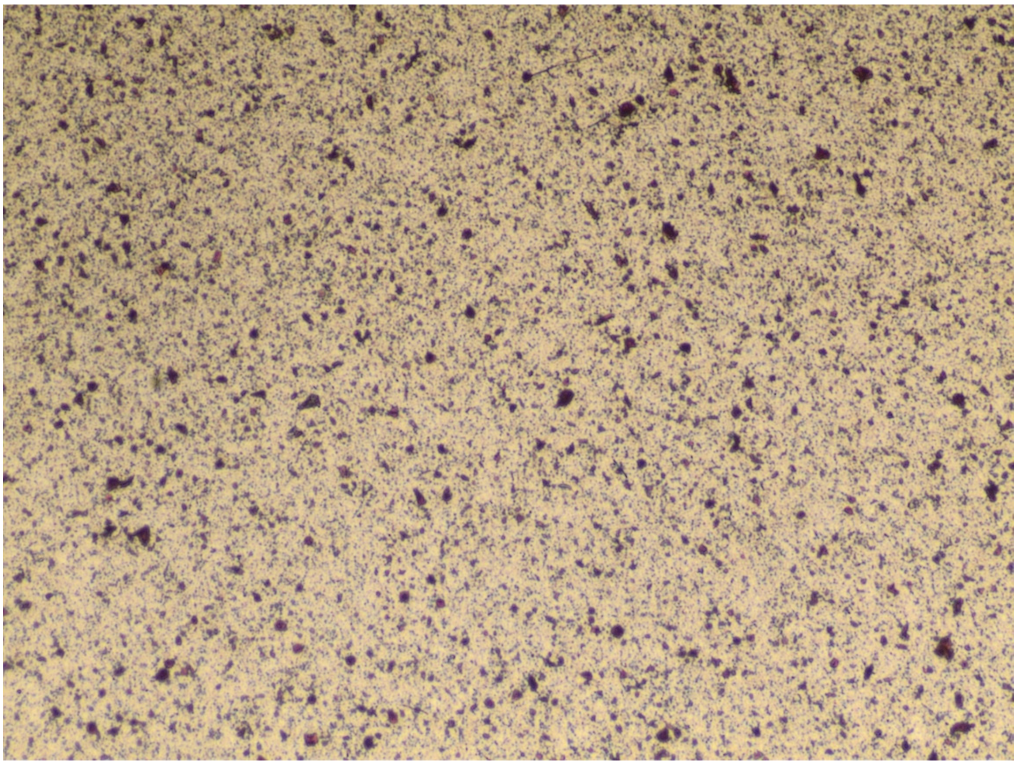

Figure S23a: OM image of MMM-5*, micrograph no.5

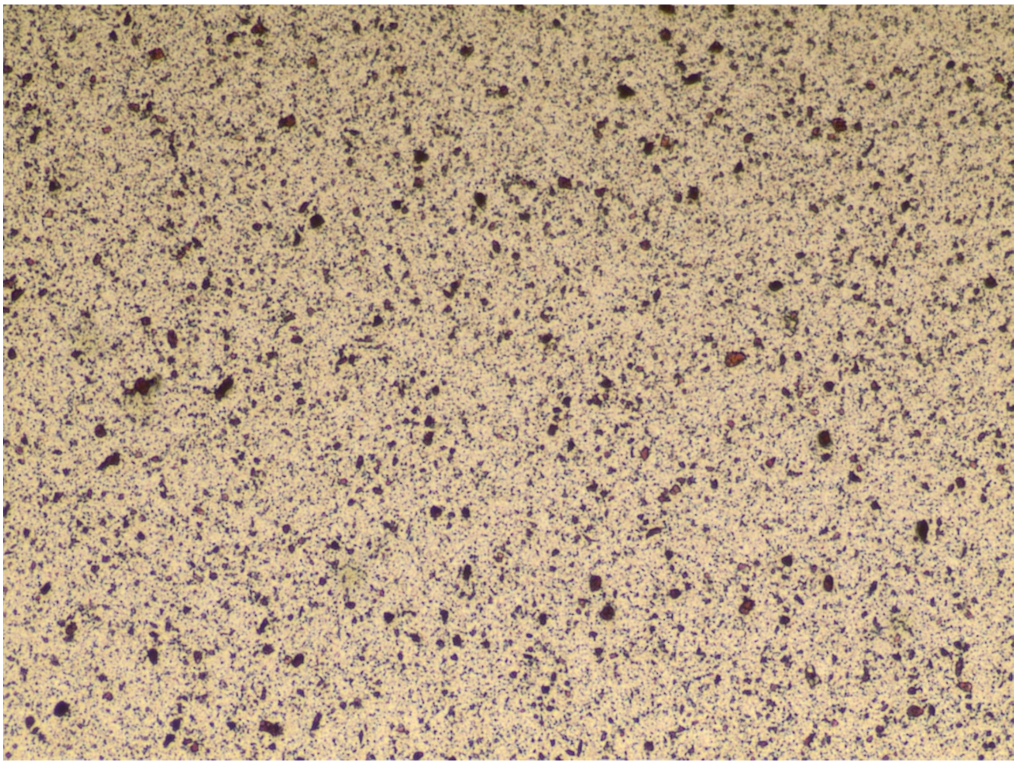

Figure S24a: OM image of MMM-5*, micrograph no.6

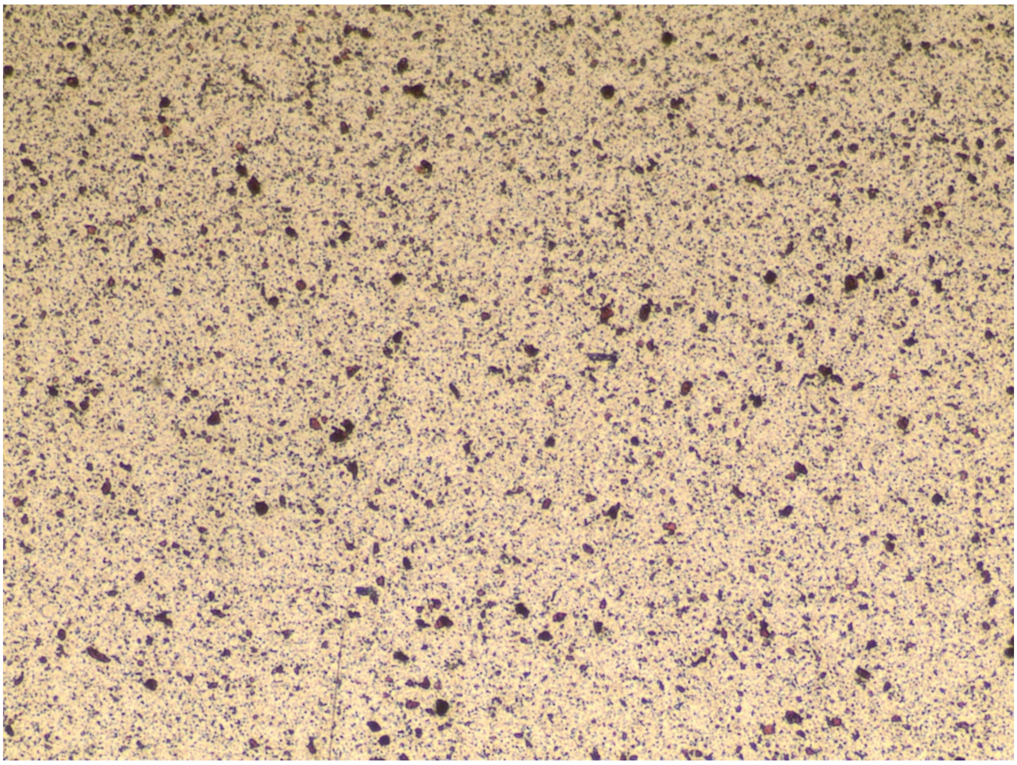

Figure S25a: OM image of MMM-5*, micrograph no.7

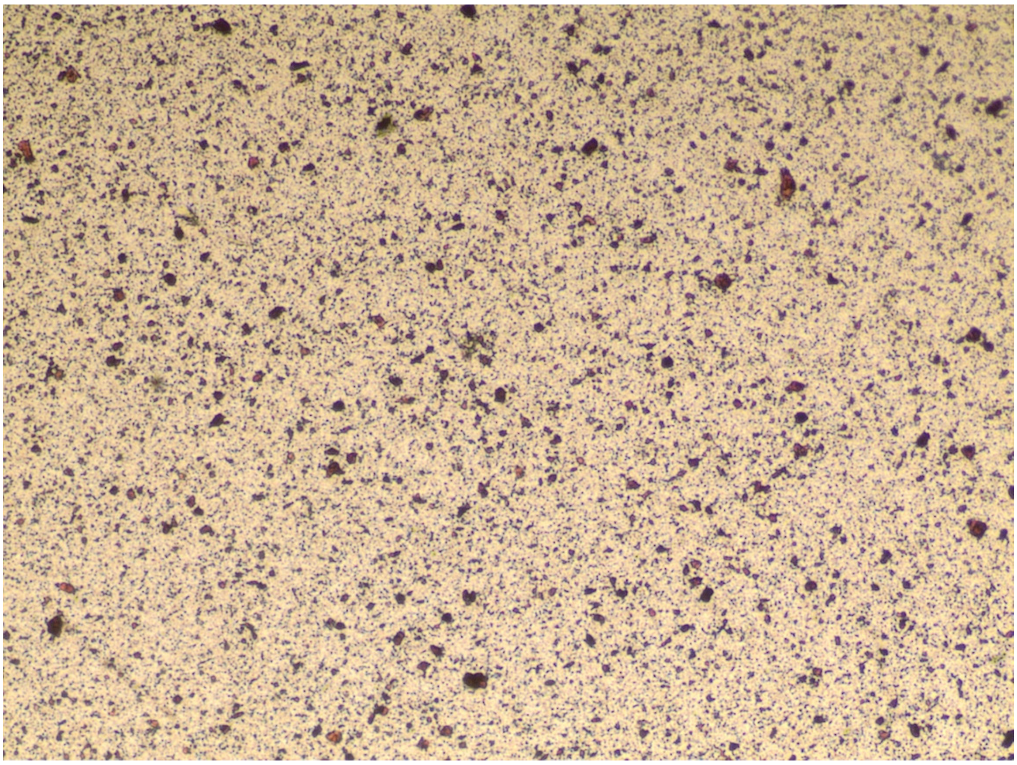

Figure S26a: OM image of MMM-5*, micrograph no.8

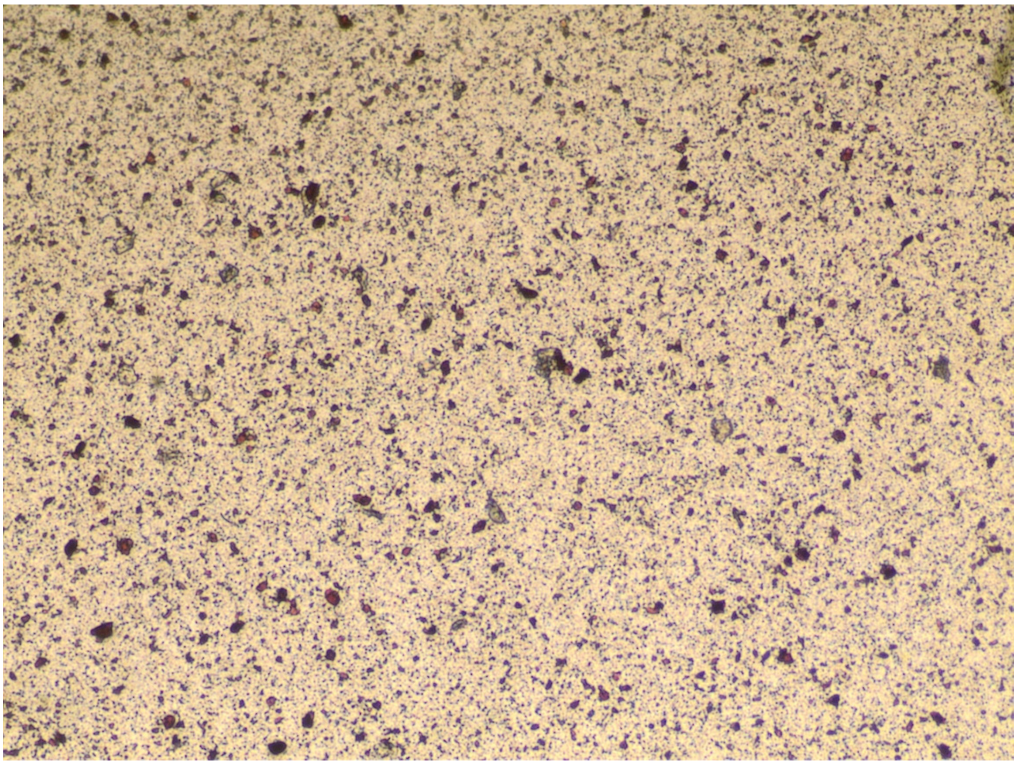

Figure S27a: OM image of MMM-5*, micrograph no.9

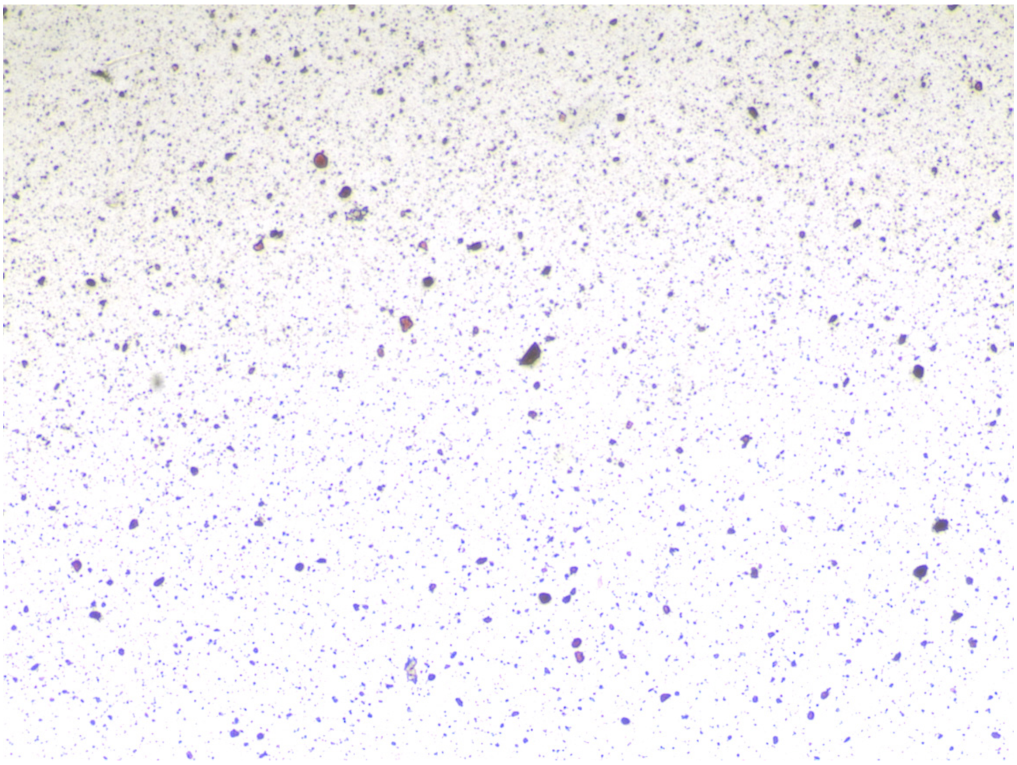

Figure S28a: OM image of MMM-1, micrograph no.1

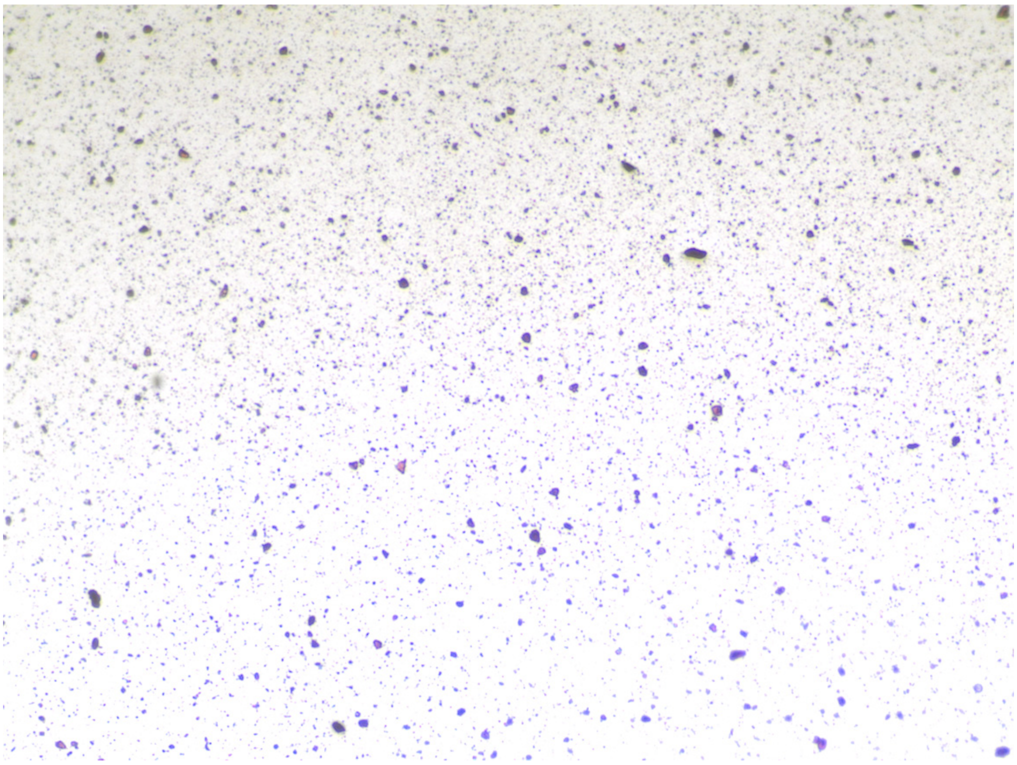

Figure S29a: OM image of MMM-1, micrograph no.2

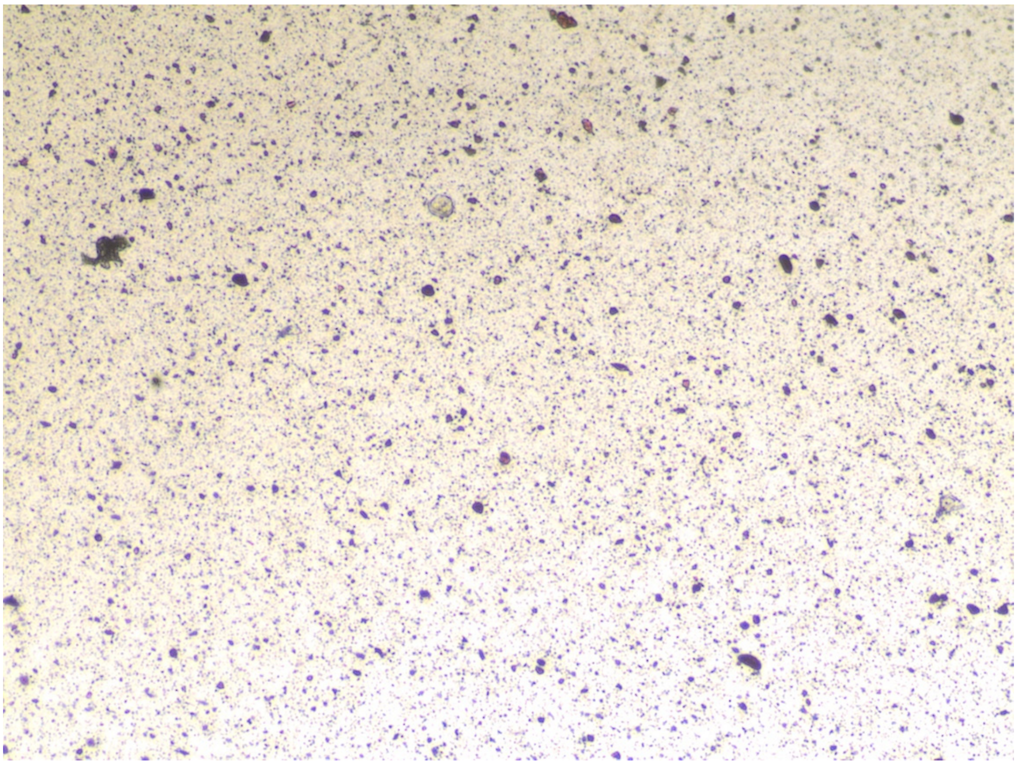

Figure S30a: OM image of MMM-1, micrograph no.3

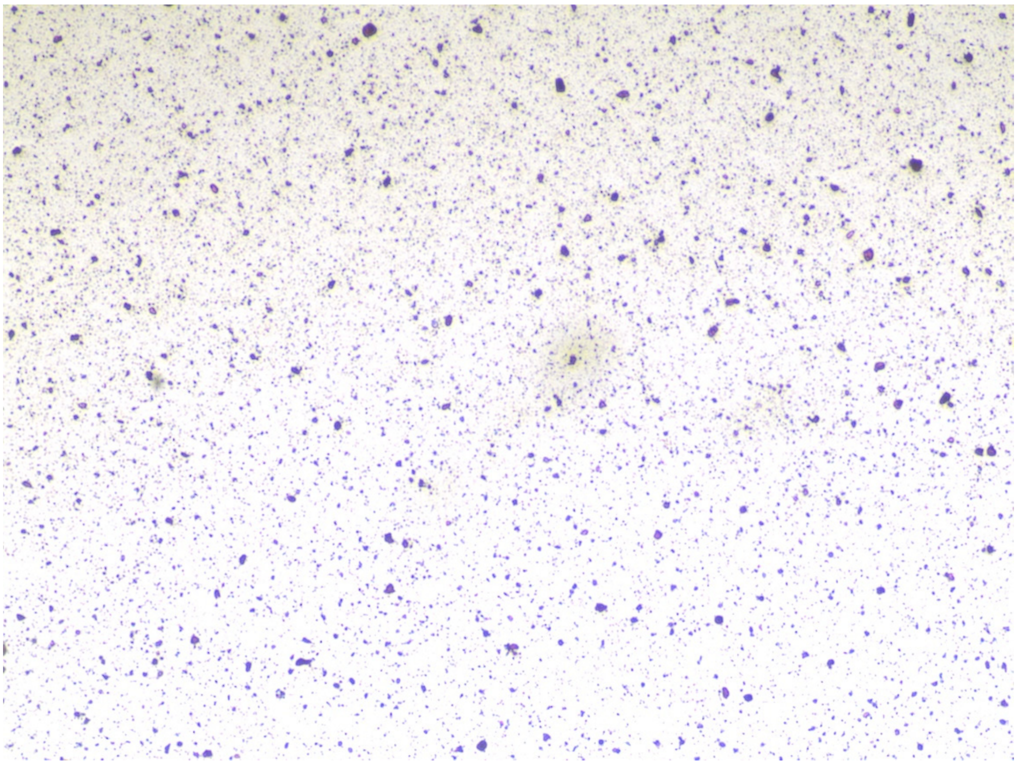

Figure S31a: OM image of MMM-1, micrograph no.4

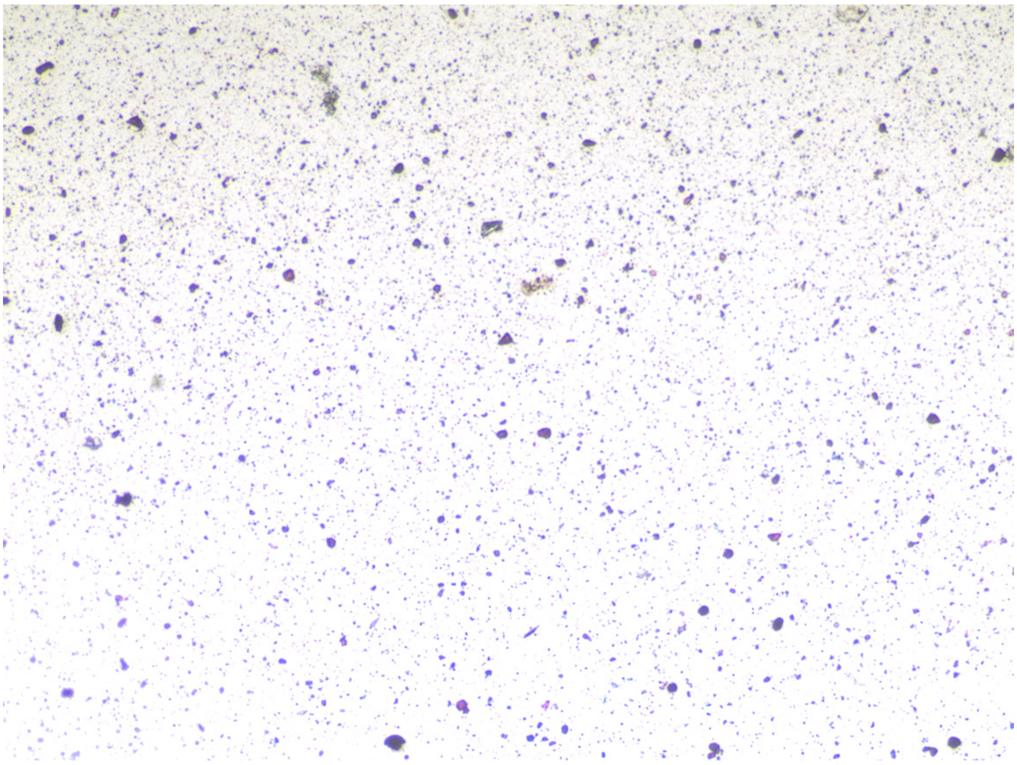

Figure S32a: OM image of MMM-1, micrograph no.5

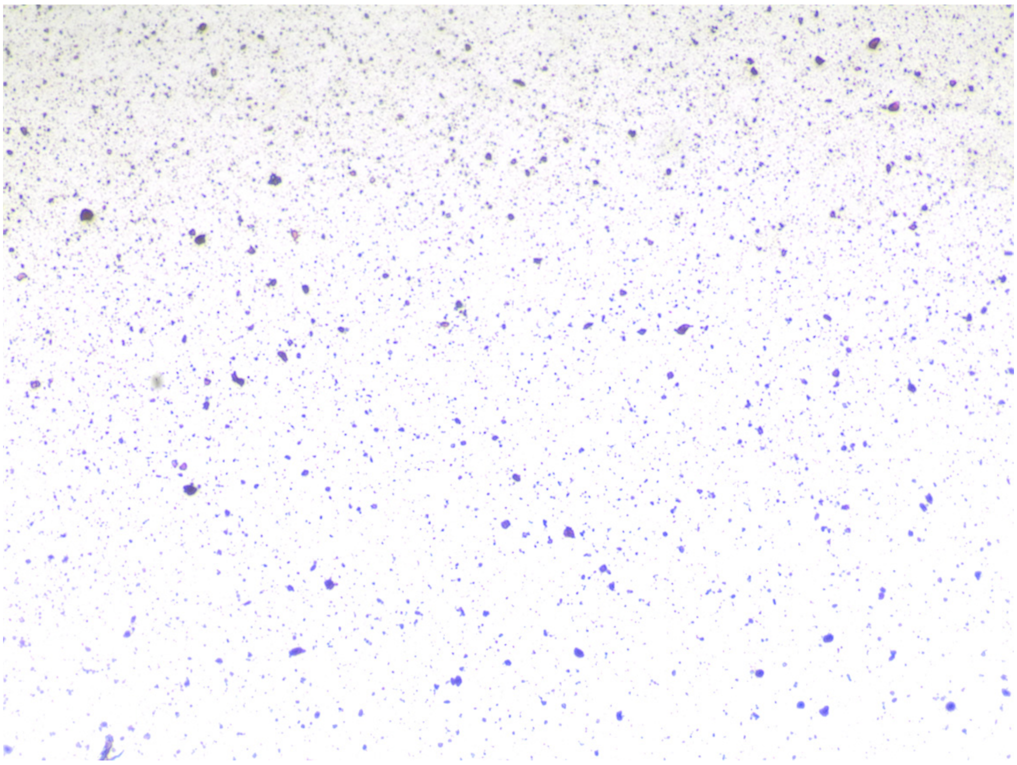

Figure S33a: OM image of MMM-1, micrograph no.6

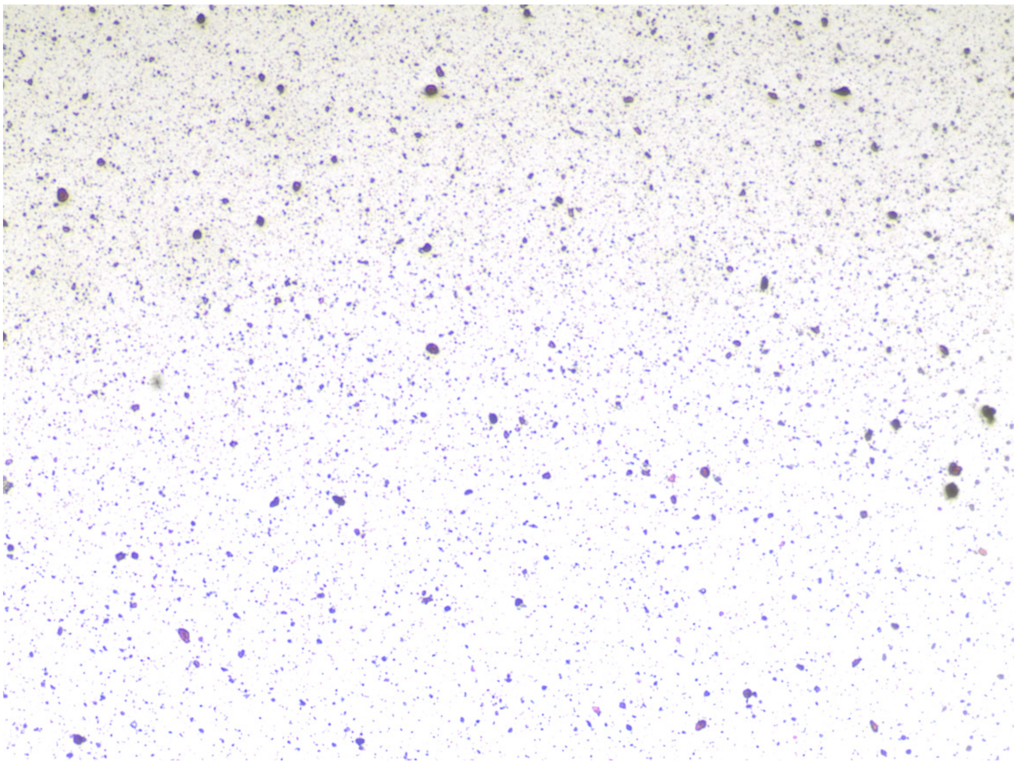

Figure S34a: OM image of MMM-1, micrograph no.7

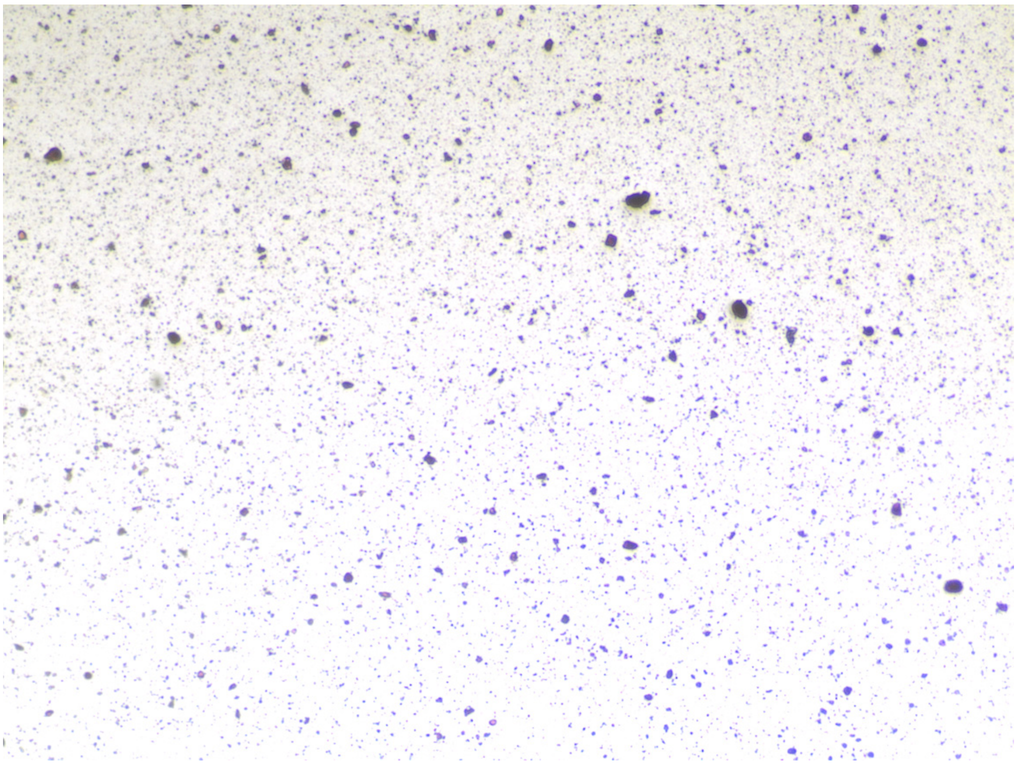

Figure S35a: OM image of MMM-1, micrograph no.8

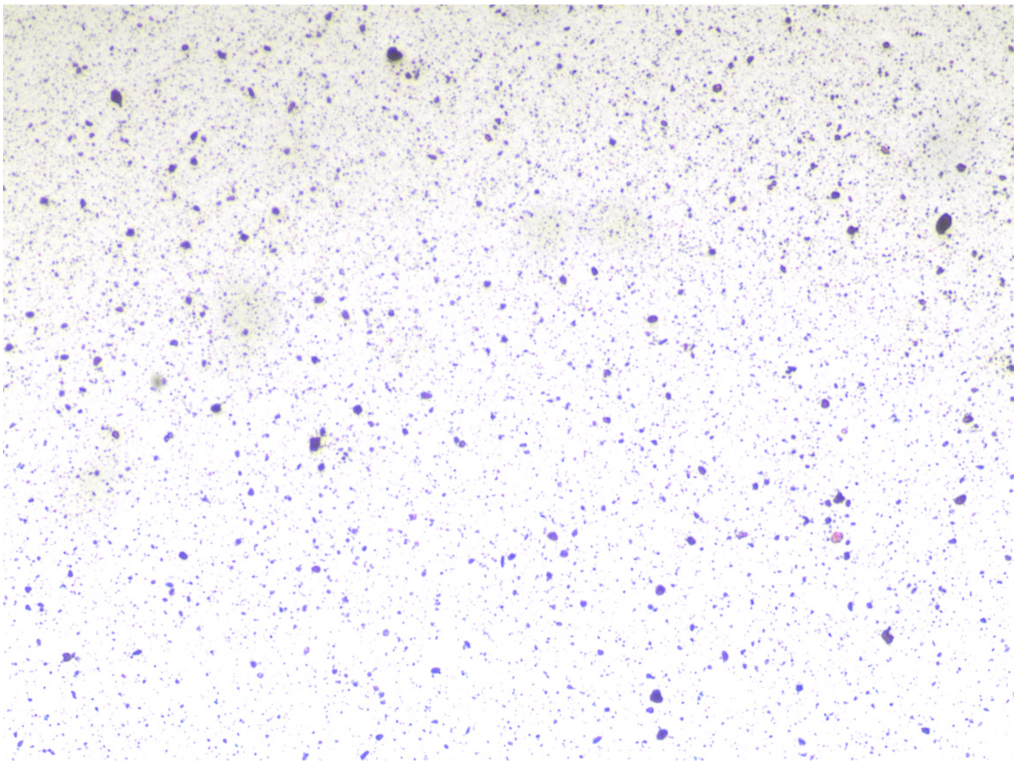

Figure S36a: OM image of MMM-1, micrograph no.9

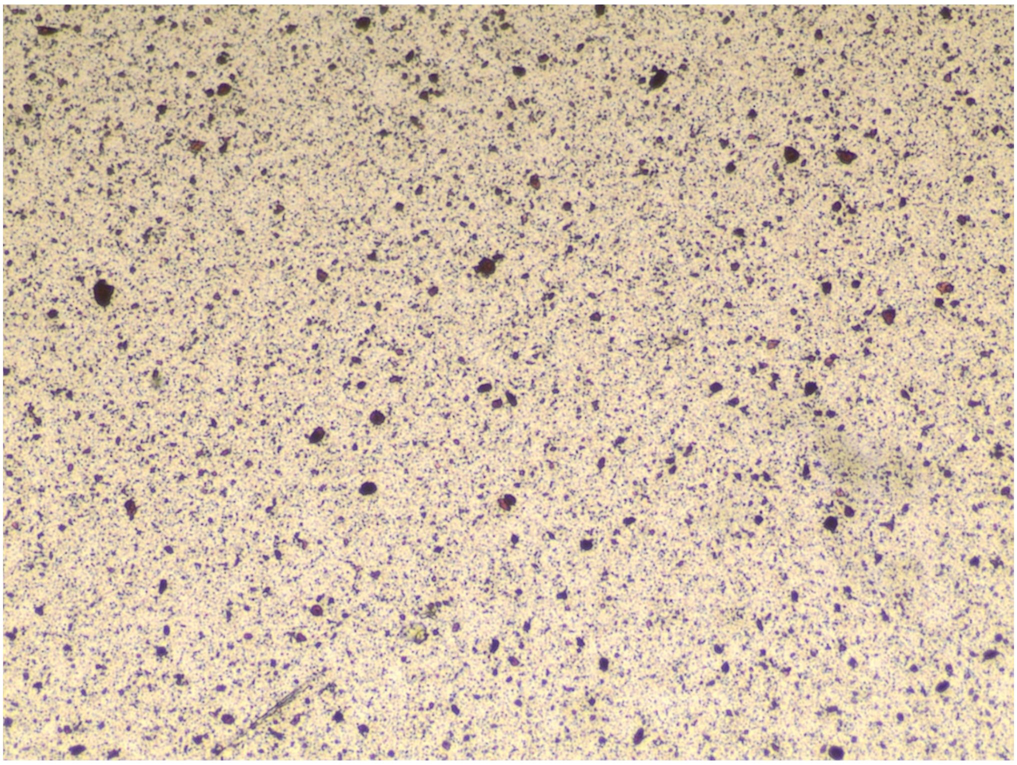

Figure S37a: OM image of MMM-3, micrograph no.1

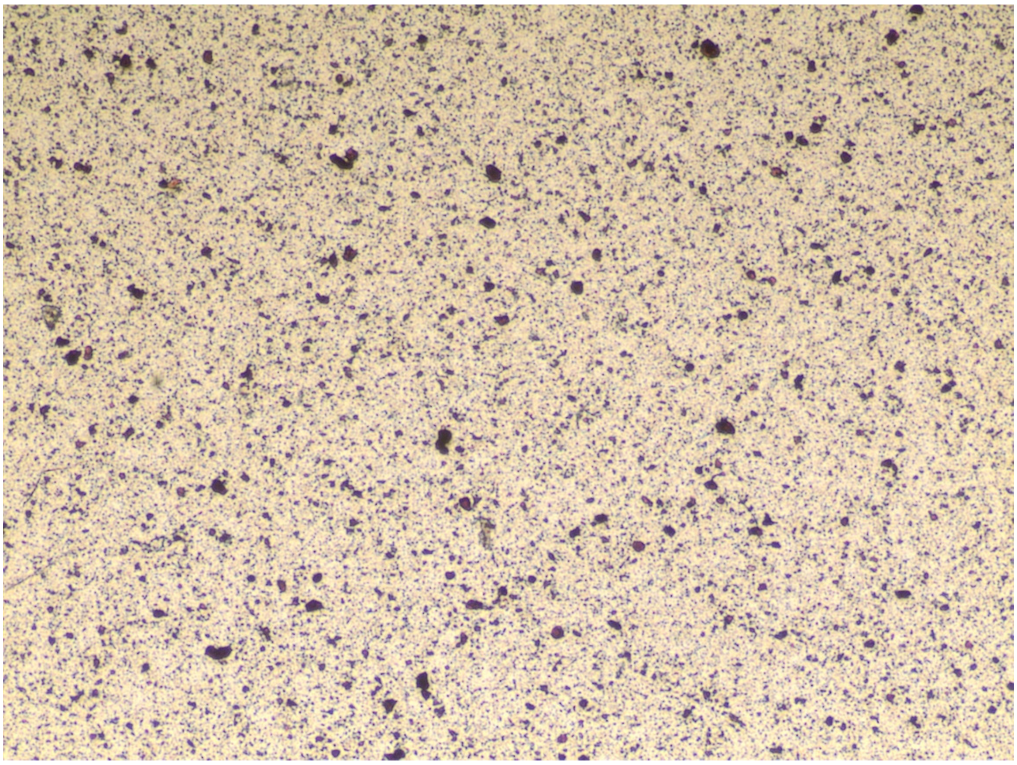

Figure S38a: OM image of MMM-3, micrograph no.2

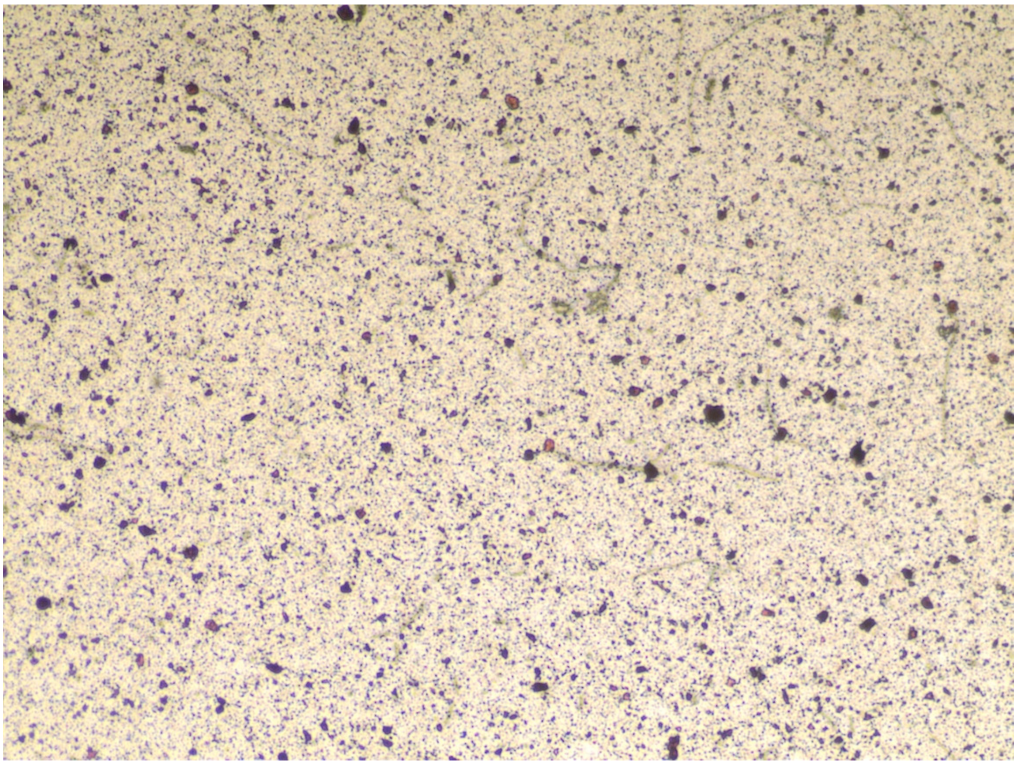

Figure S39a: OM image of MMM-3, micrograph no.3

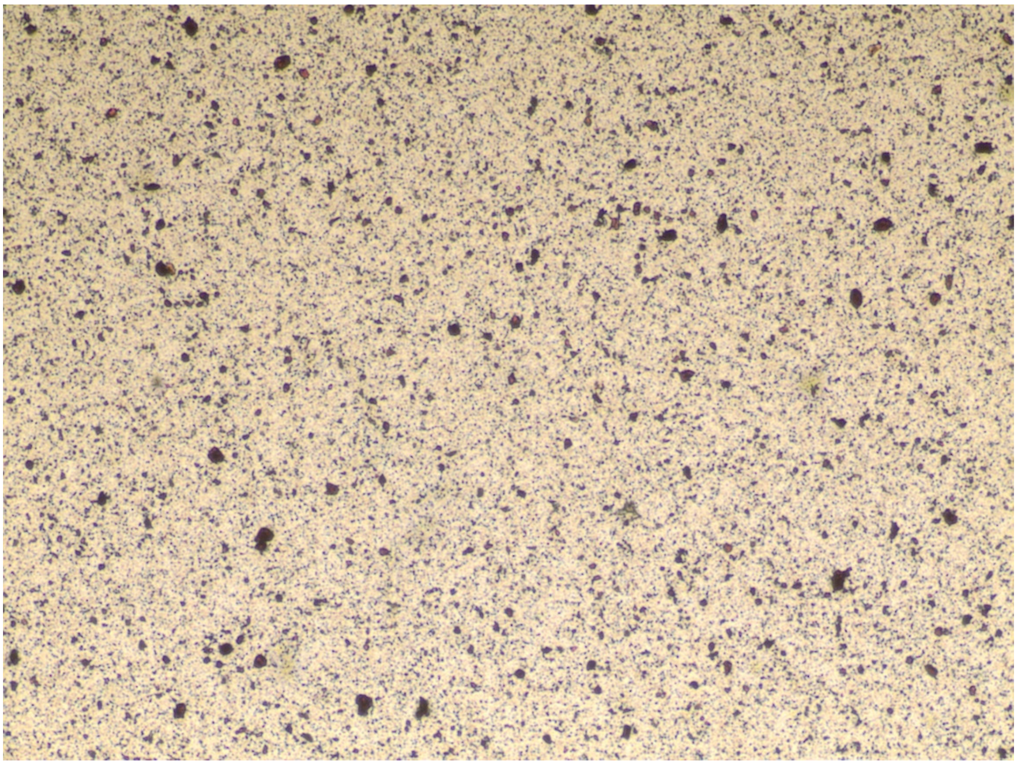

Figure S40a: OM image of MMM-3, micrograph no.4

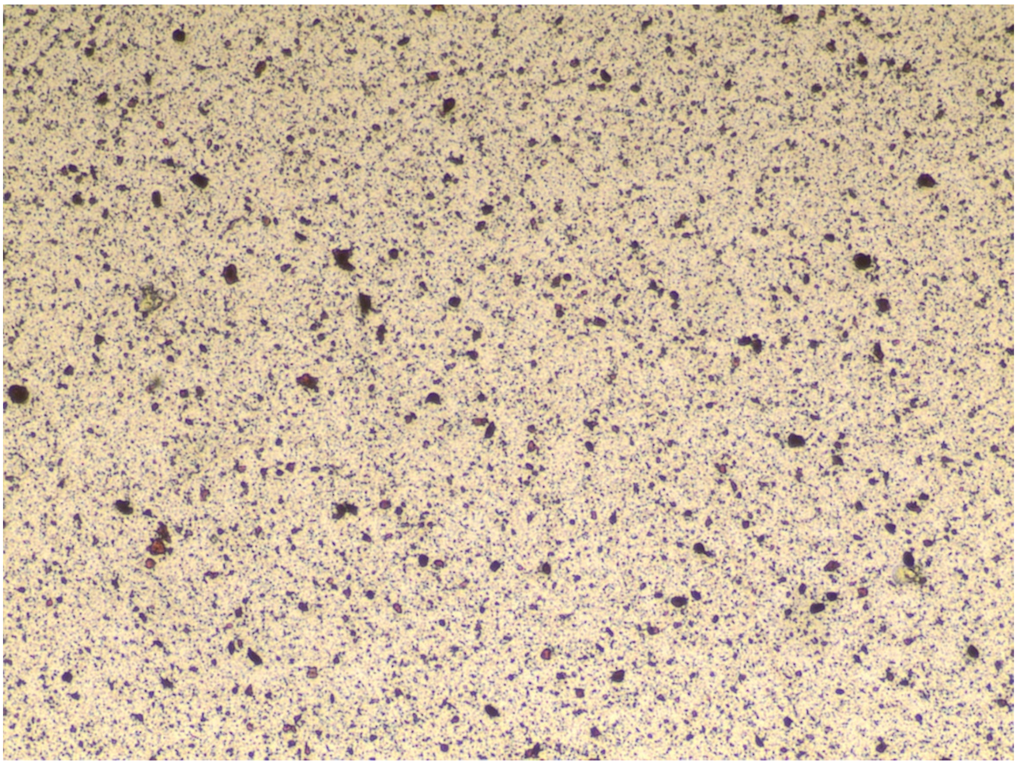

Figure S41a: OM image of MMM-3, micrograph no.5

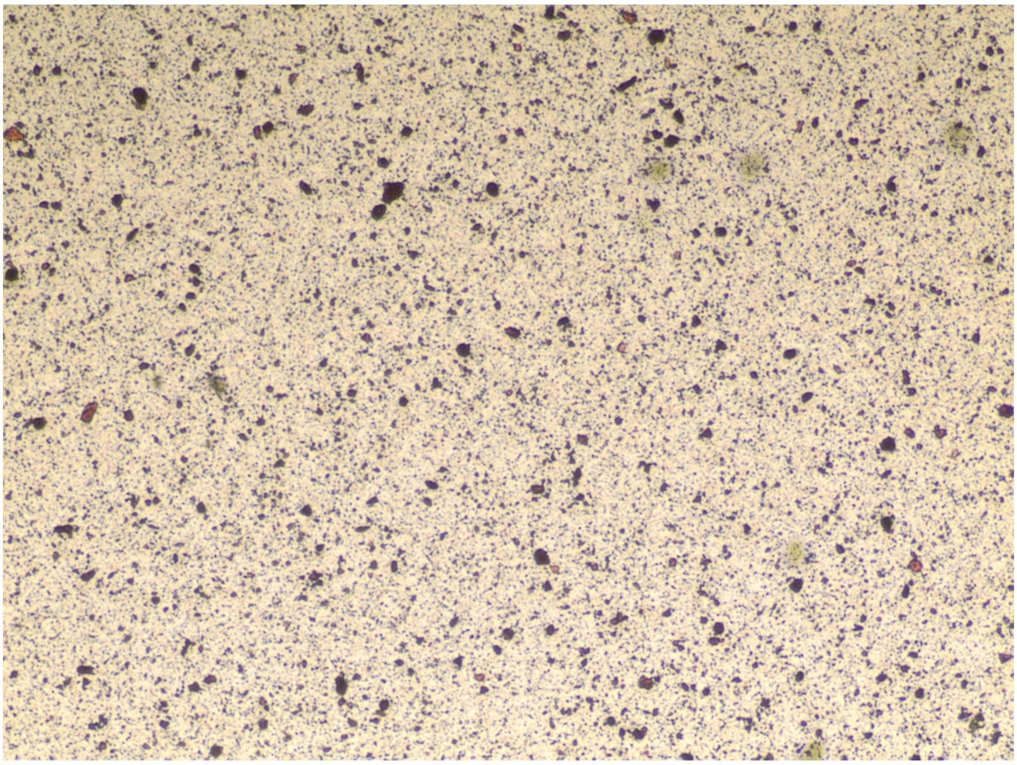

Figure S42a: OM image of MMM-3, micrograph no.6

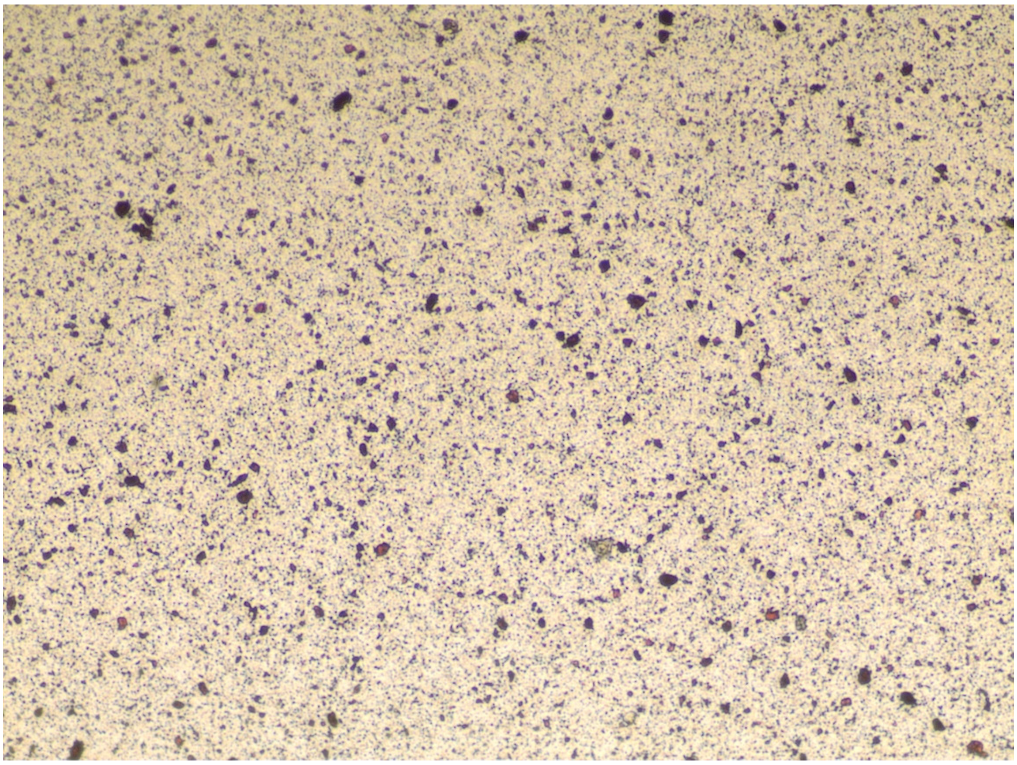

Figure S43a: OM image of MMM-3, micrograph no.7

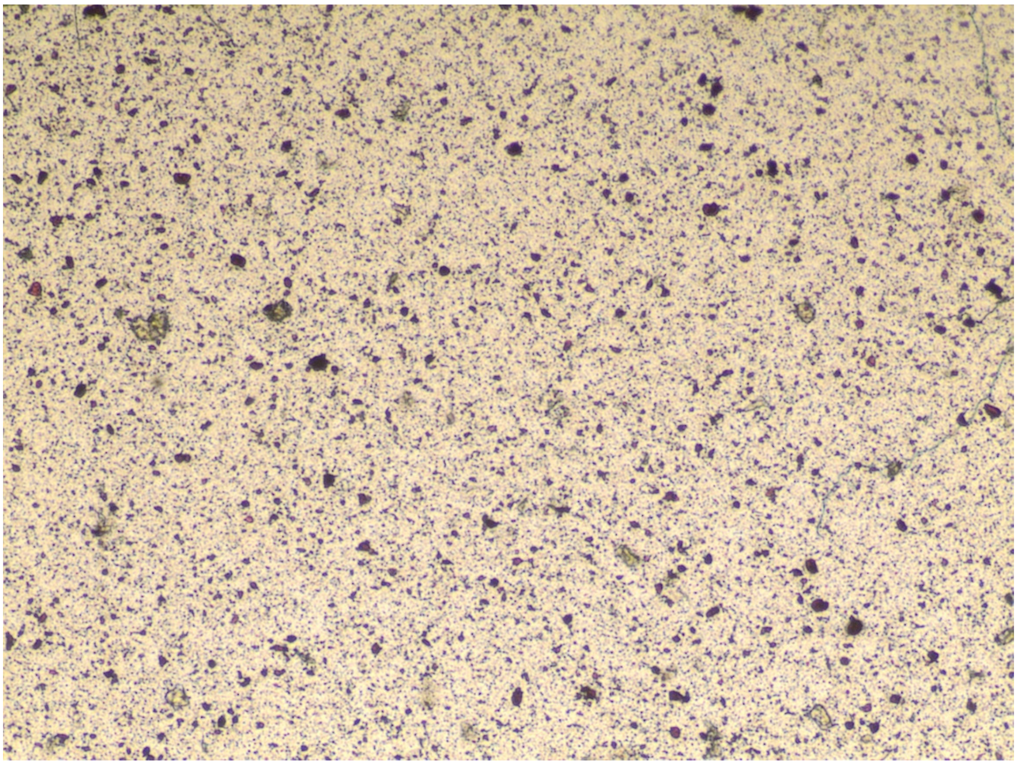

Figure S44a: OM image of MMM-3, micrograph no.8

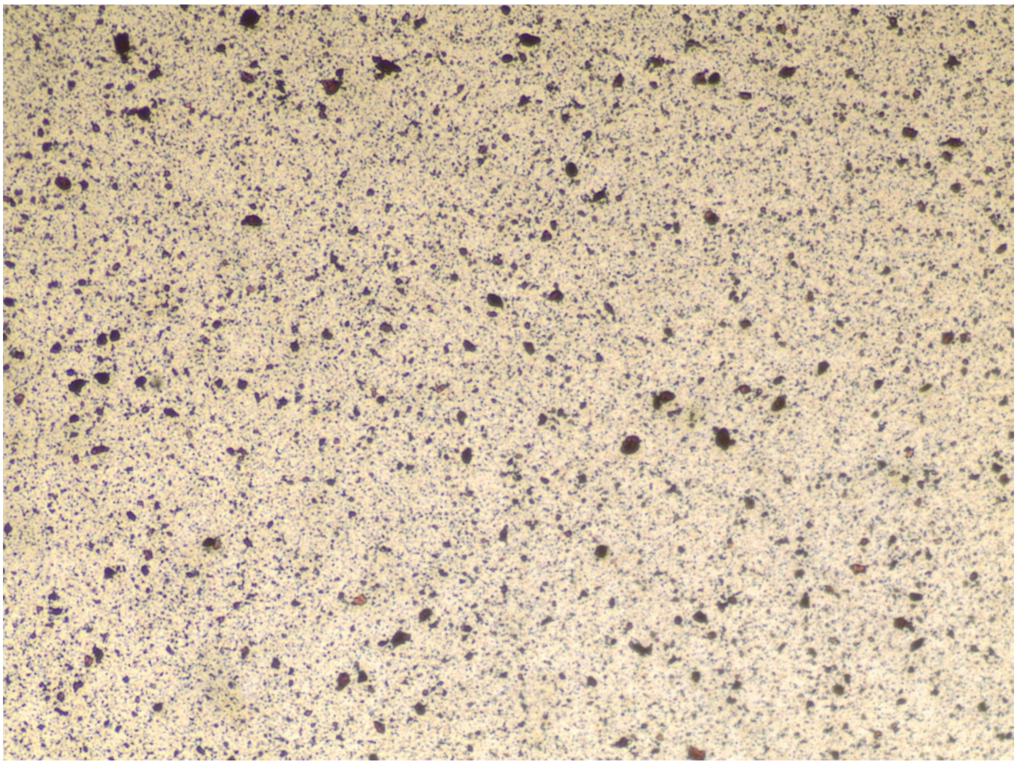

Figure S45a: OM image of MMM-3, micrograph no.9

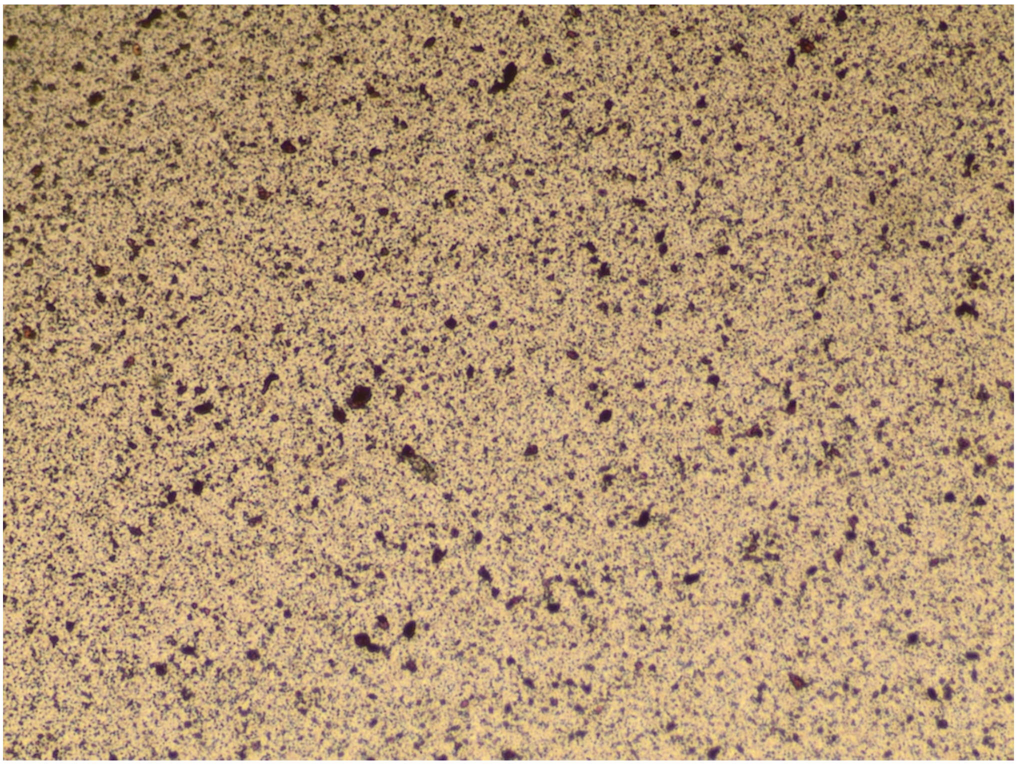

Figure S46a: OM image of MMM-5, micrograph no.1

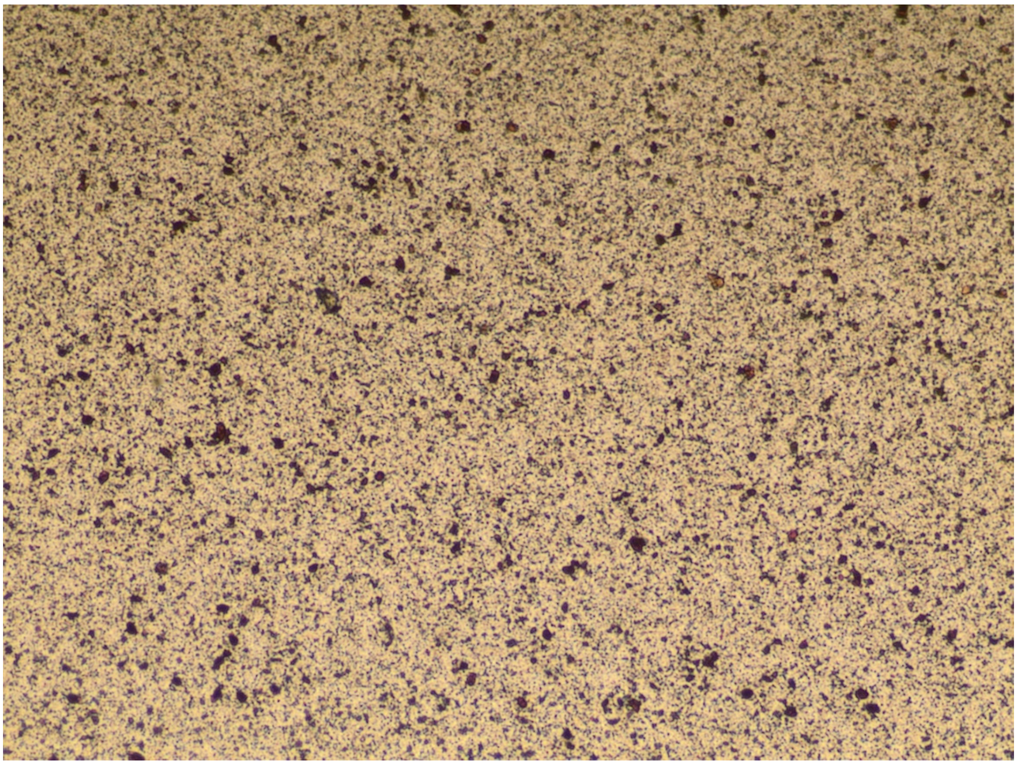

Figure S47a: OM image of MMM-5, micrograph no.2

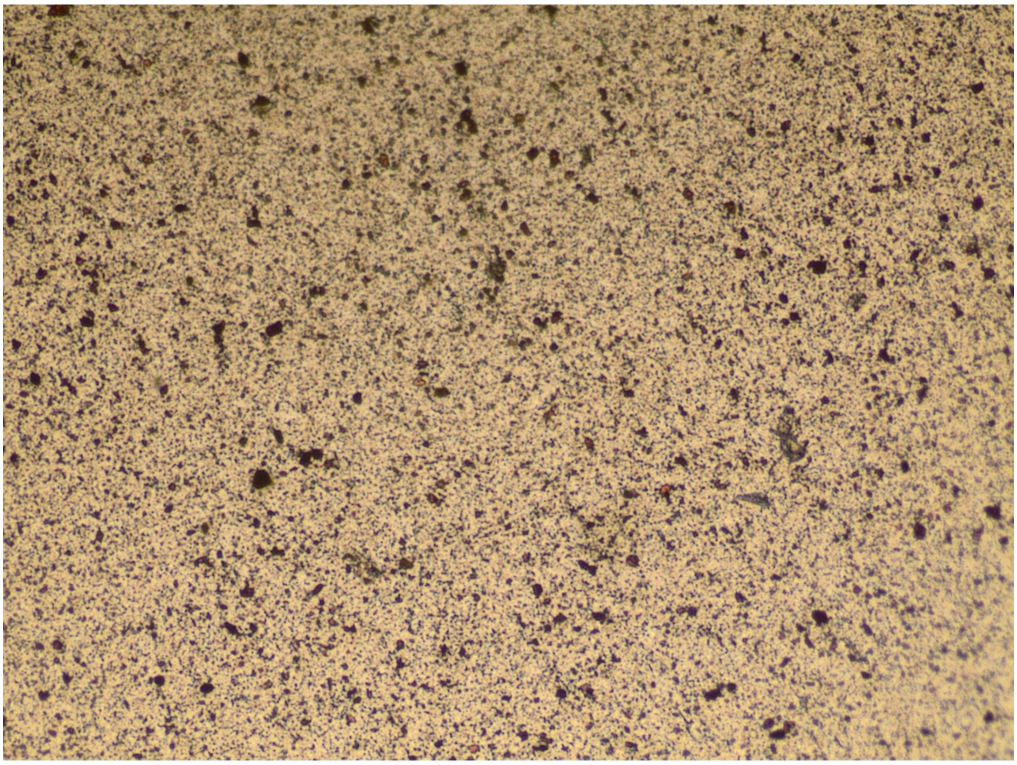

Figure S48a: OM image of MMM-5, micrograph no.3

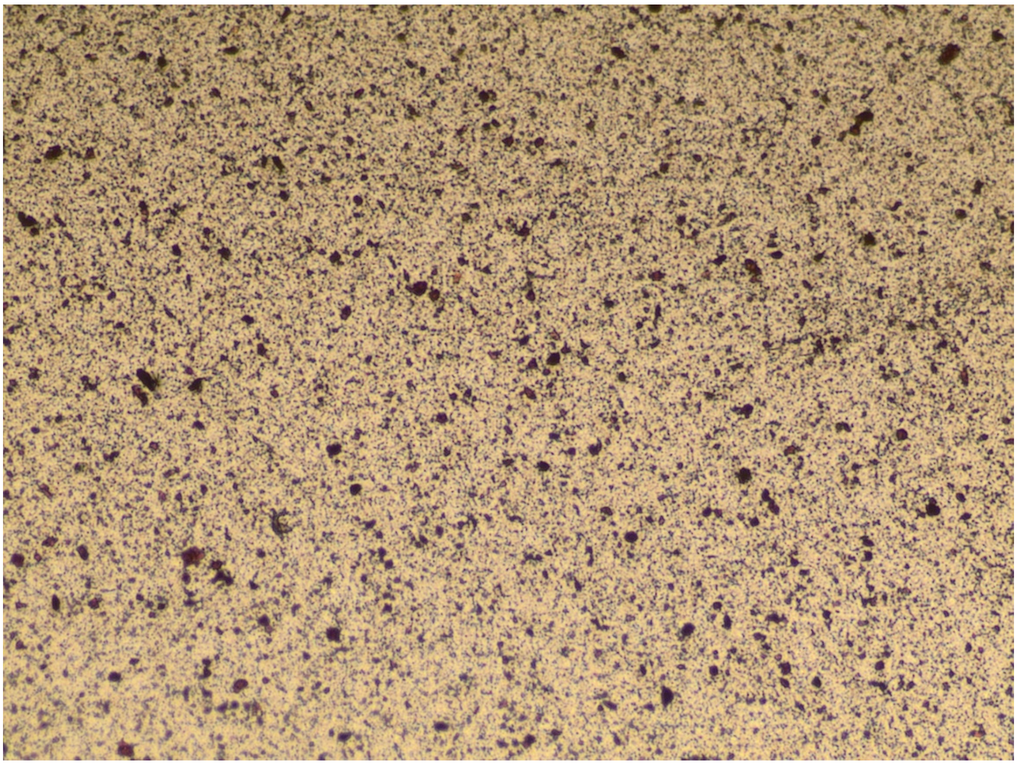

Figure S49a: OM image of MMM-5, micrograph no.4

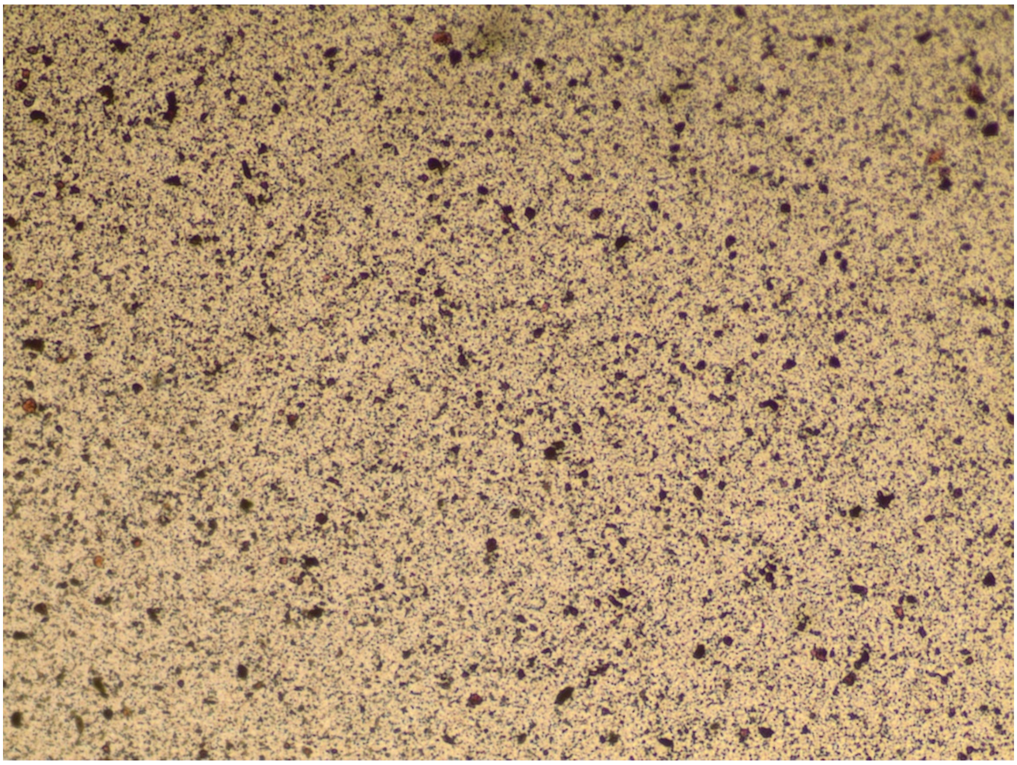

Figure S50a: OM image of MMM-5, micrograph no.5

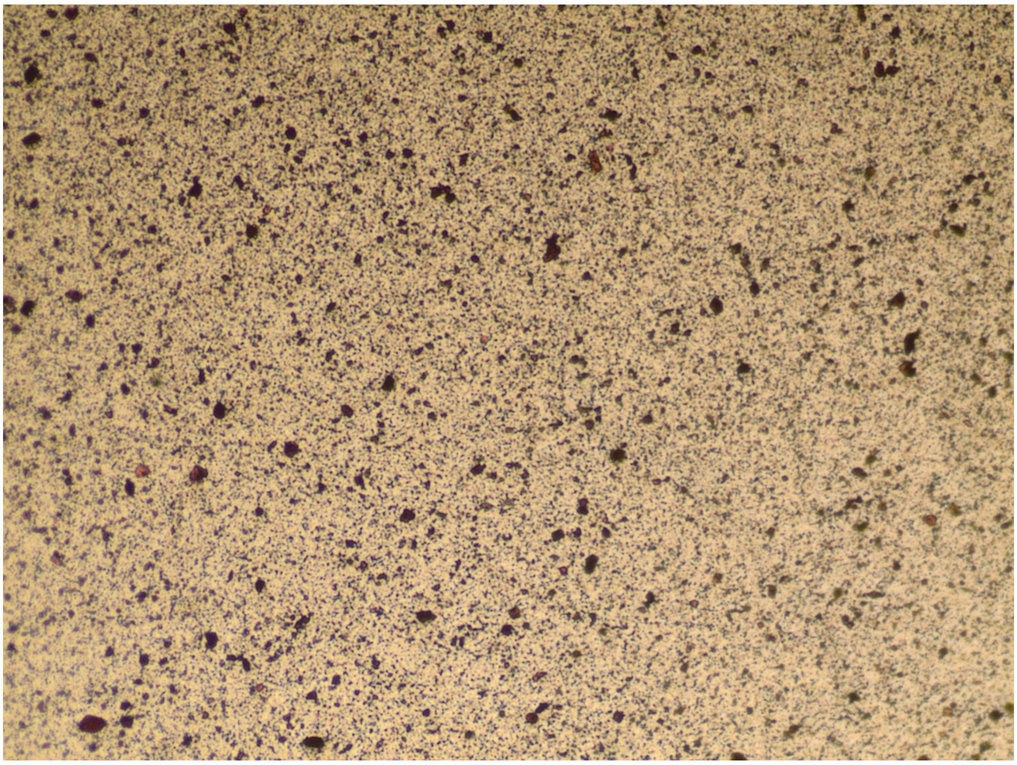

Figure S51a: OM image of MMM-5, micrograph no.6

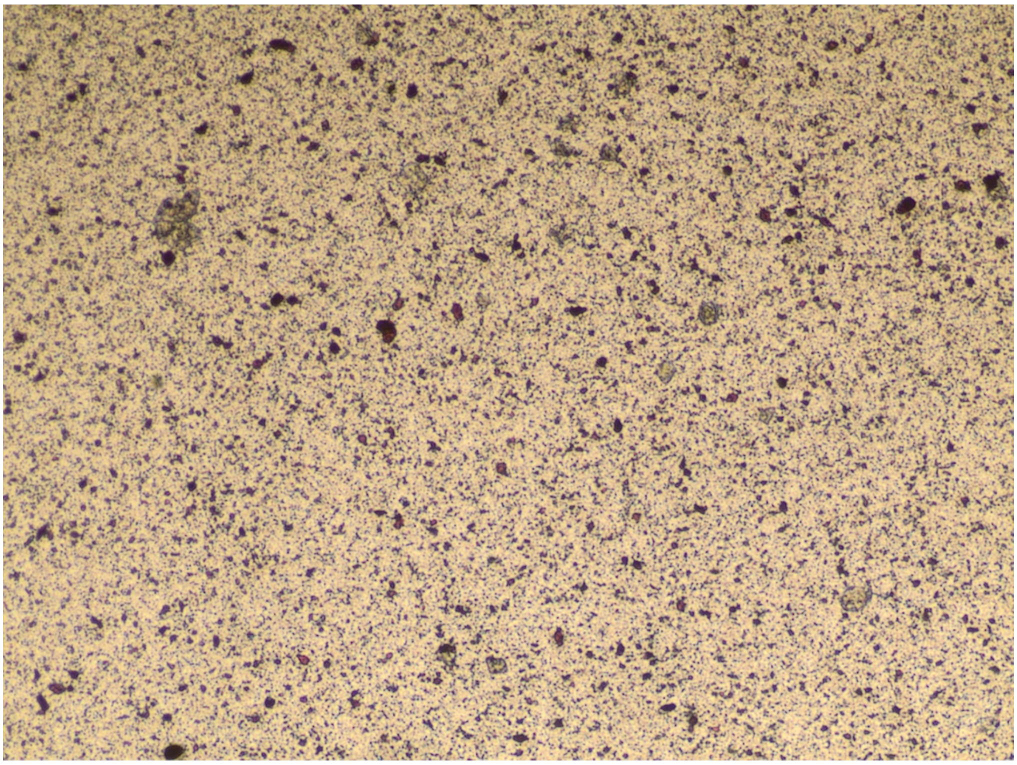

Figure S52a: OM image of MMM-5, micrograph no.7

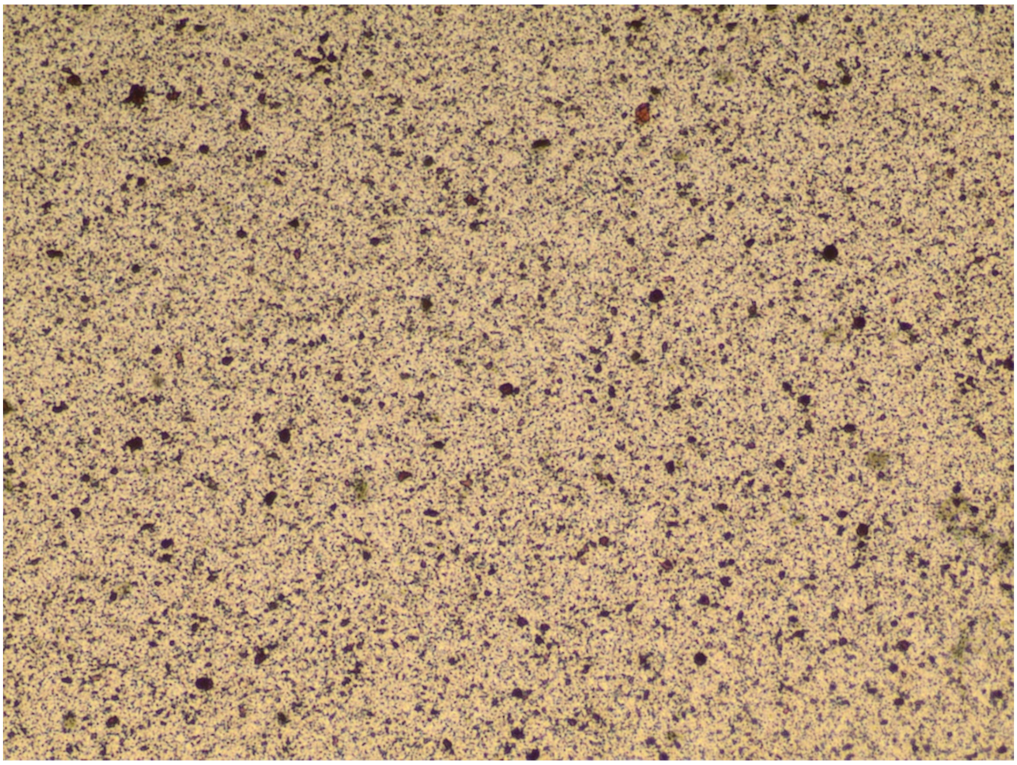

Figure S53a: OM image of MMM-5, micrograph no.8

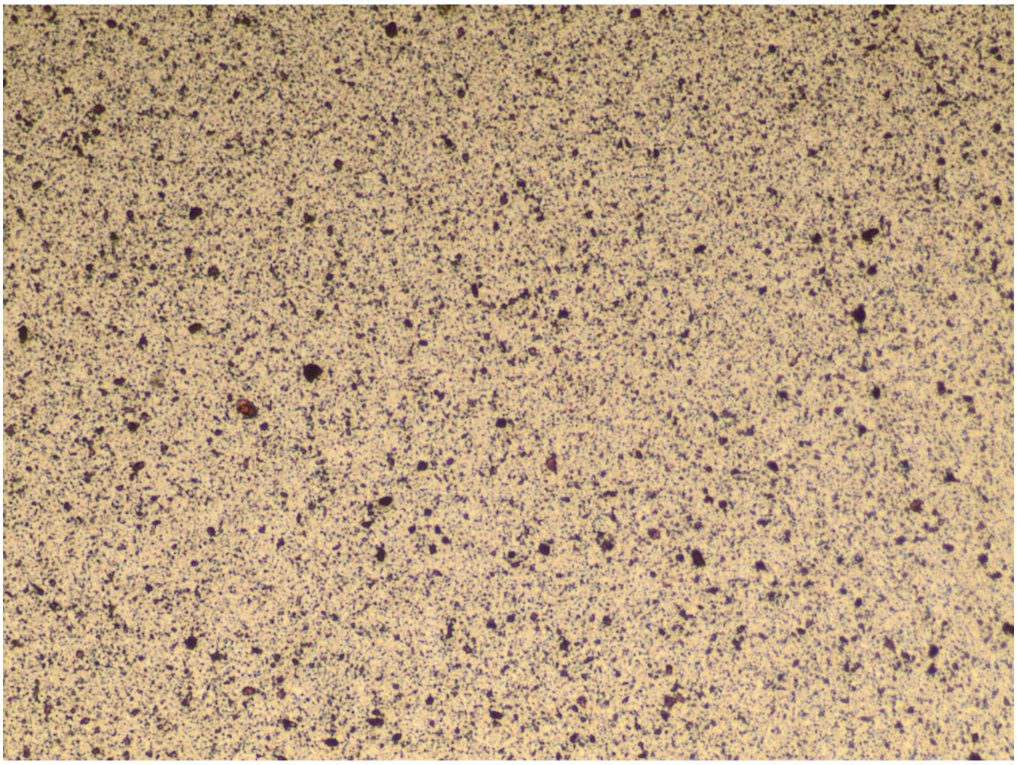

Figure S54a: OM image of MMM-5, micrograph no.9

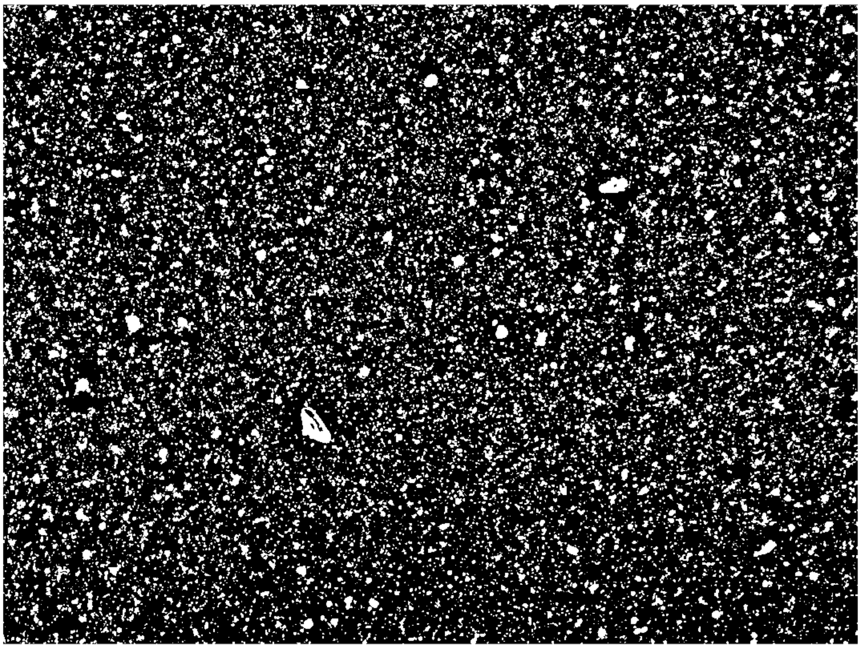

Figure S1b: OM image of MMM-1*, micrograph no.1

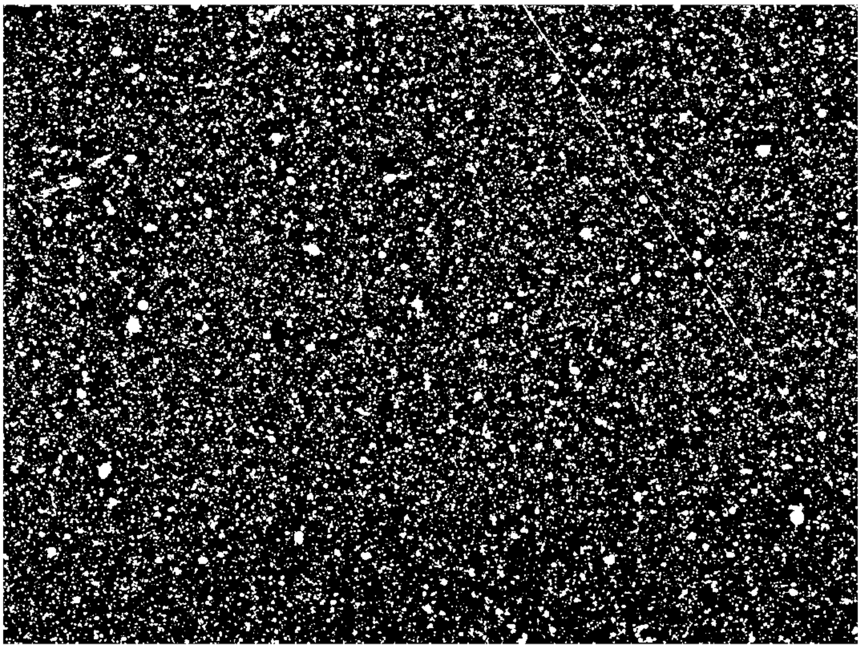

Figure S2b: OM image of MMM-1*, micrograph no.2

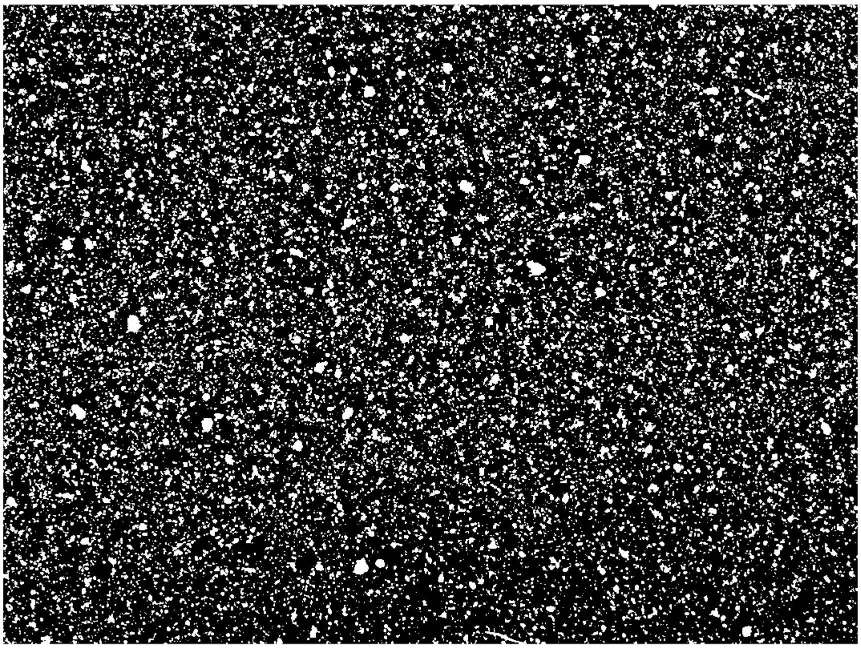

Figure S3b: OM image of MMM-1*, micrograph no.3

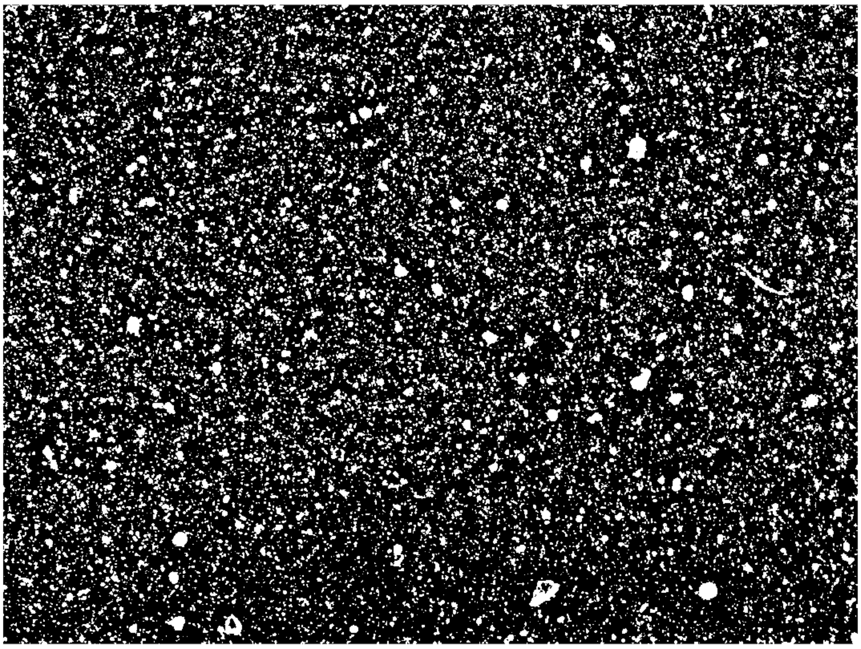

Figure S4b: OM image of MMM-1*, micrograph no.4

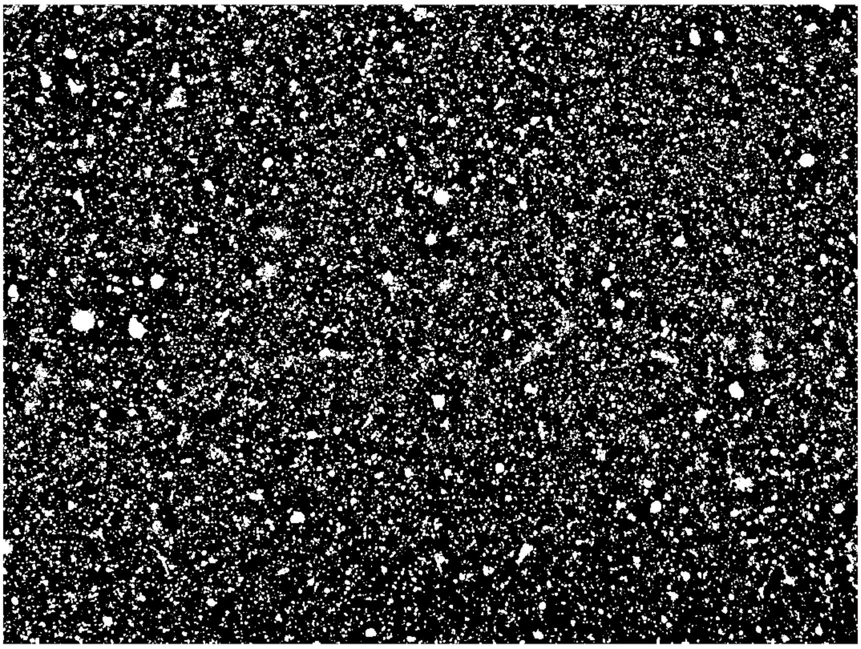

Figure S5b: OM image of MMM-1*, micrograph no.5

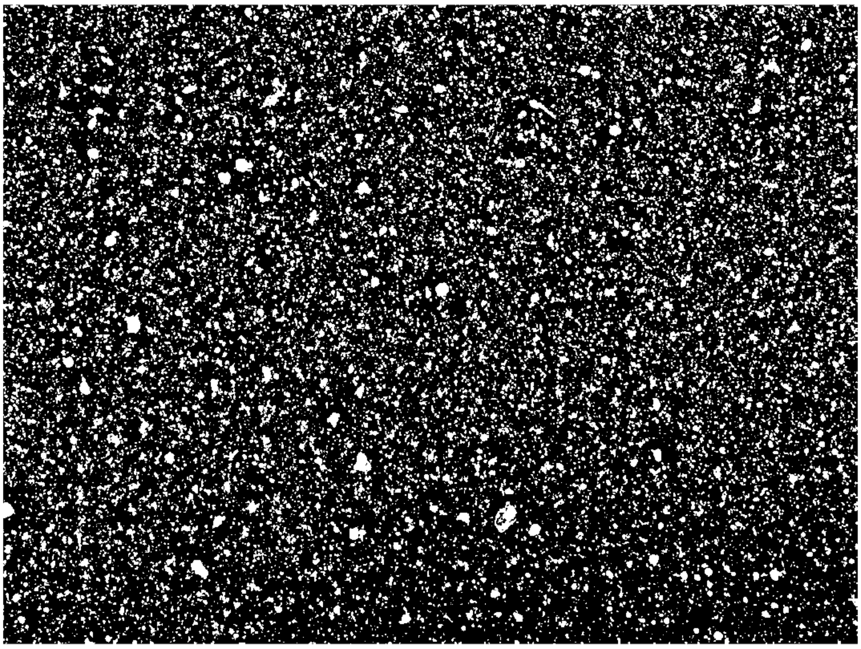

Figure S6b: OM image of MMM-1*, micrograph no.6

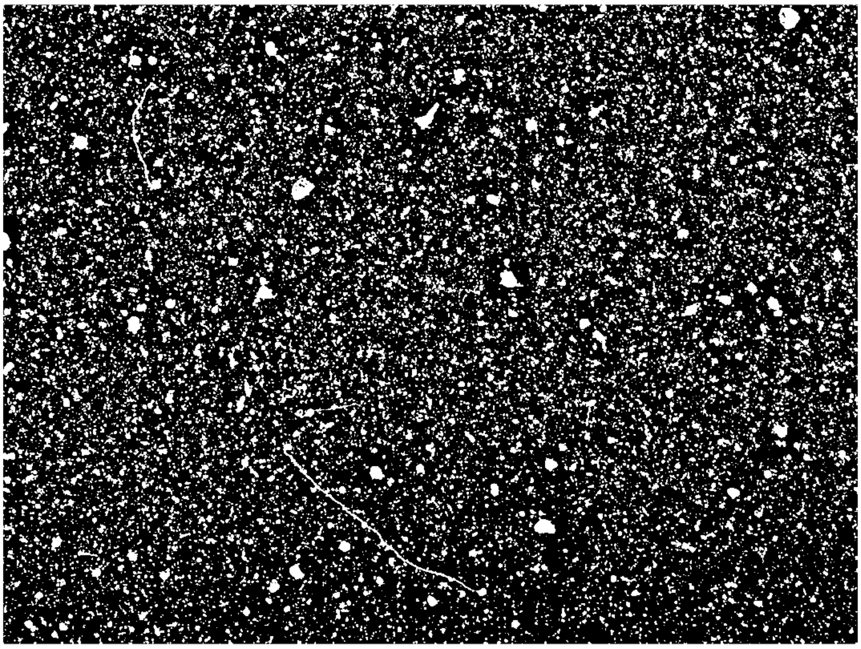

Figure S7b: OM image of MMM-1*, micrograph no.7

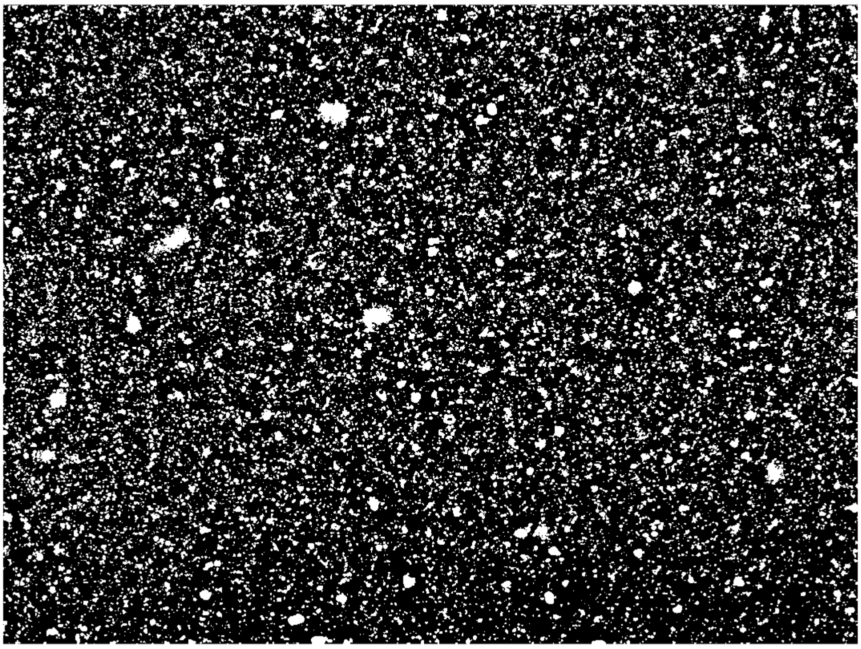

Figure S8b: OM image of MMM-1*, micrograph no.8

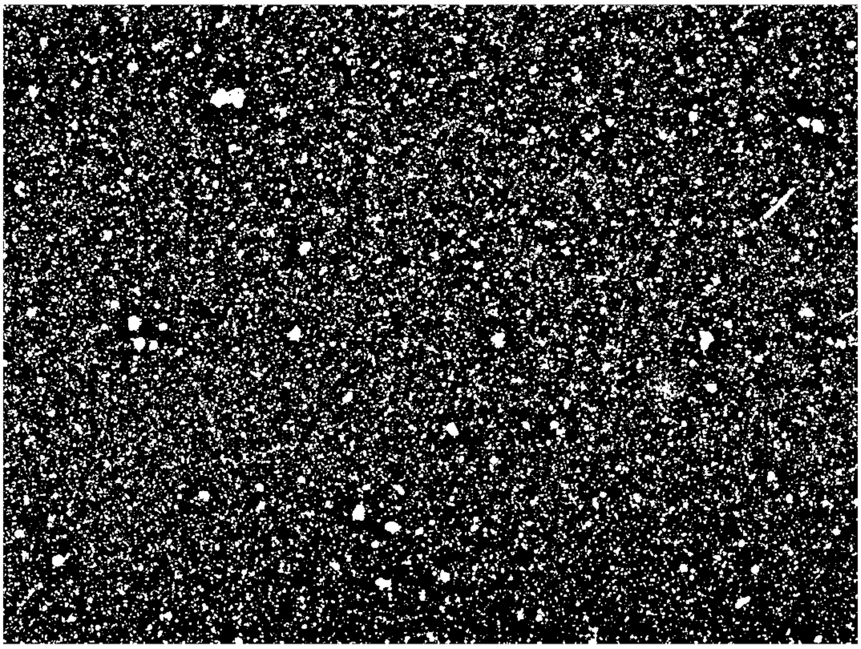

Figure S9b: OM image of MMM-1*, micrograph no.9

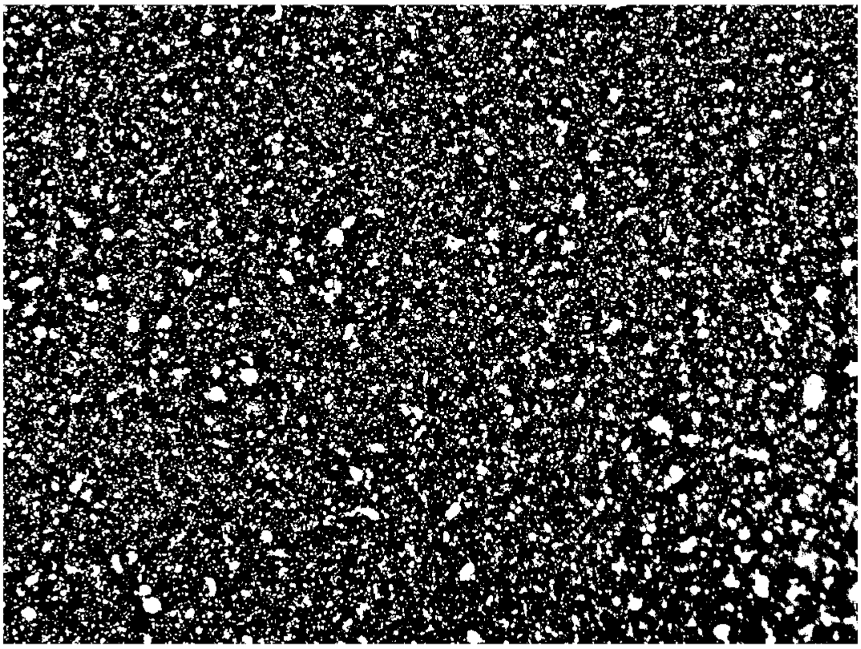

Figure S10b: OM image of MMM-3*, micrograph no.1

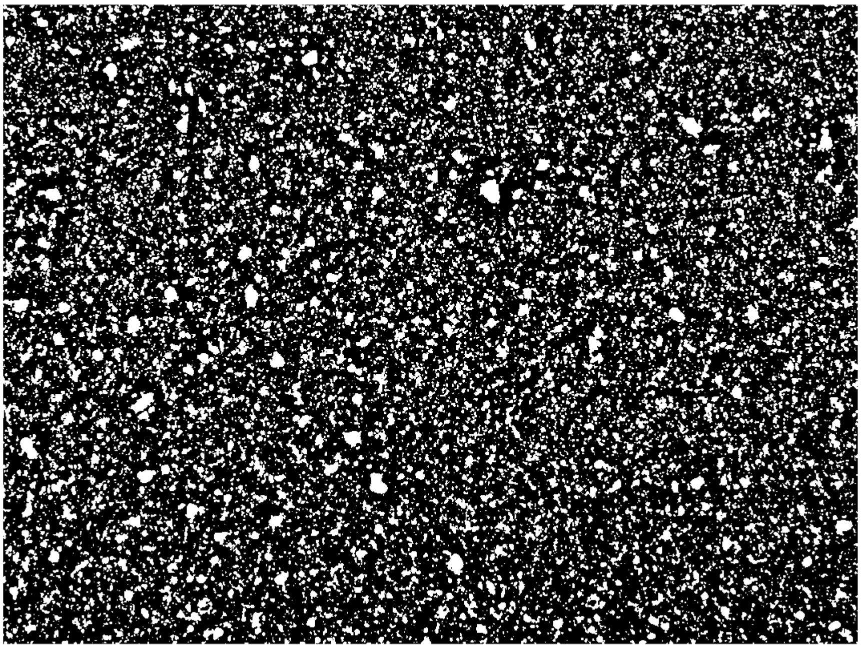

Figure S11b: OM image of MMM-3*, micrograph no.2

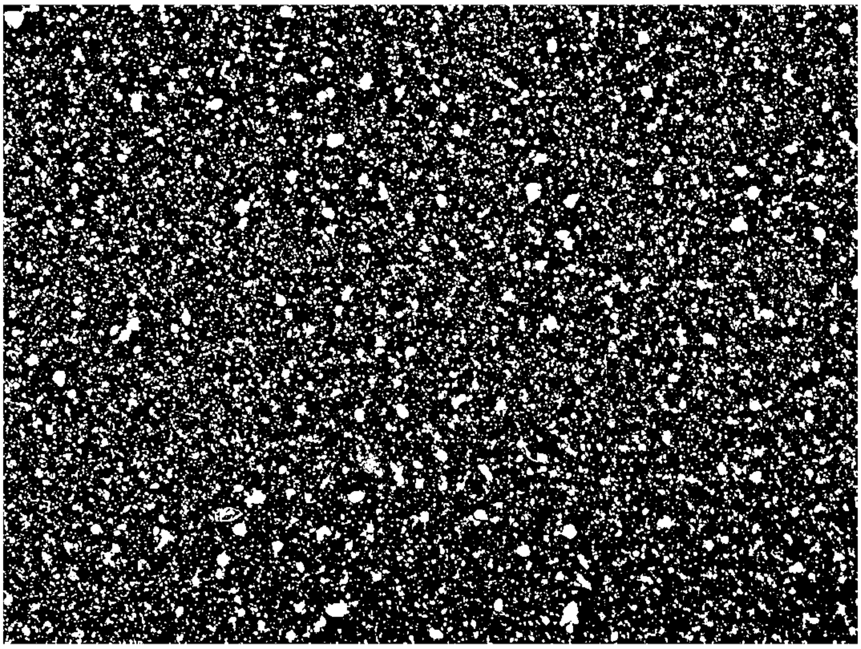

Figure S12b: OM image of MMM-3*, micrograph no.3

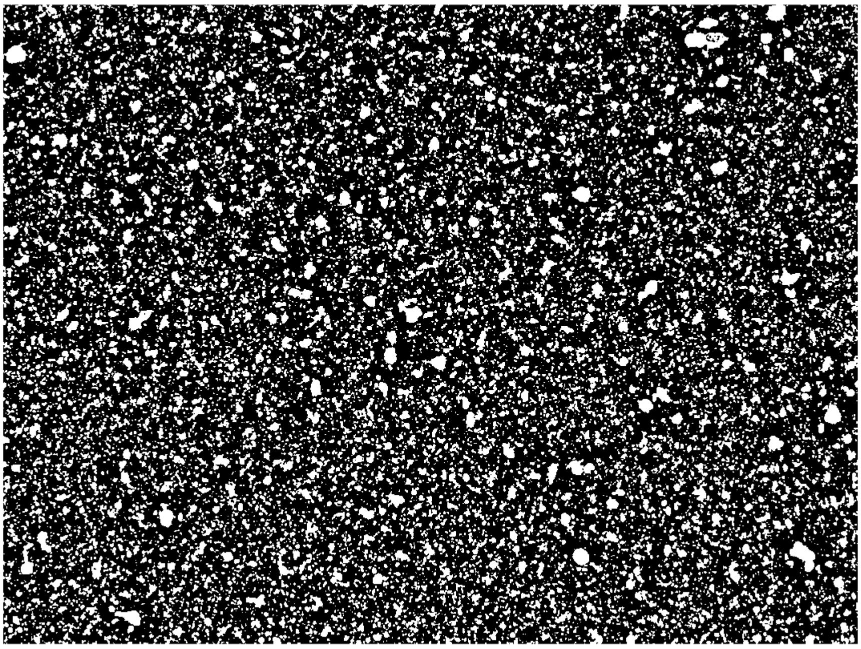

Figure S13b: OM image of MMM-3*, micrograph no.4

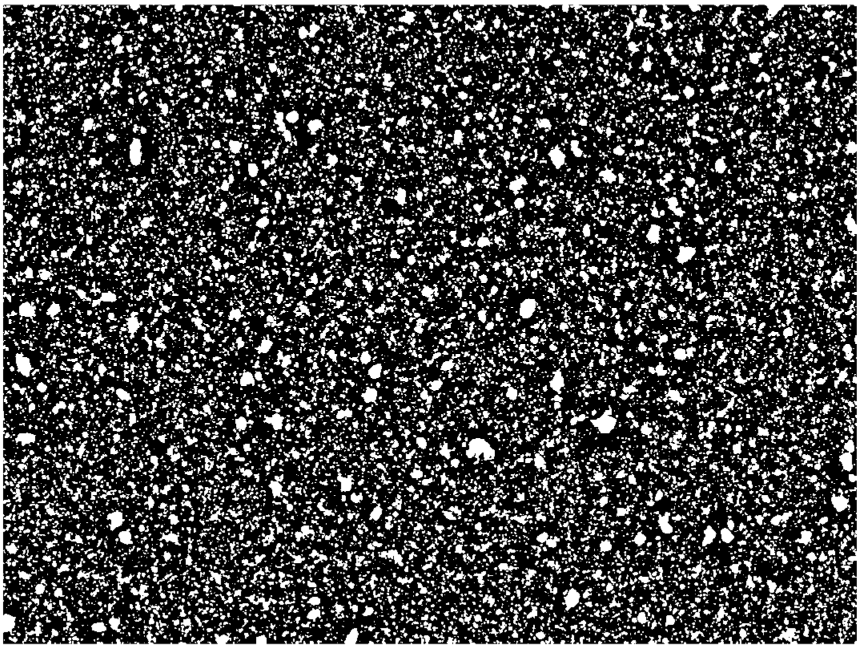

Figure S14b: OM image of MMM-3*, micrograph no.5

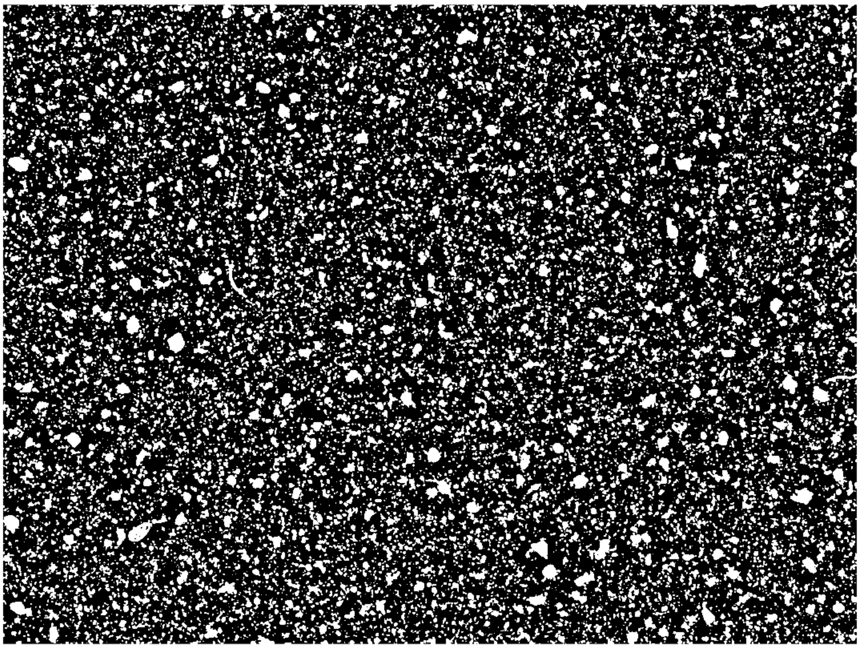

Figure S15b: OM image of MMM-3*, micrograph no.6

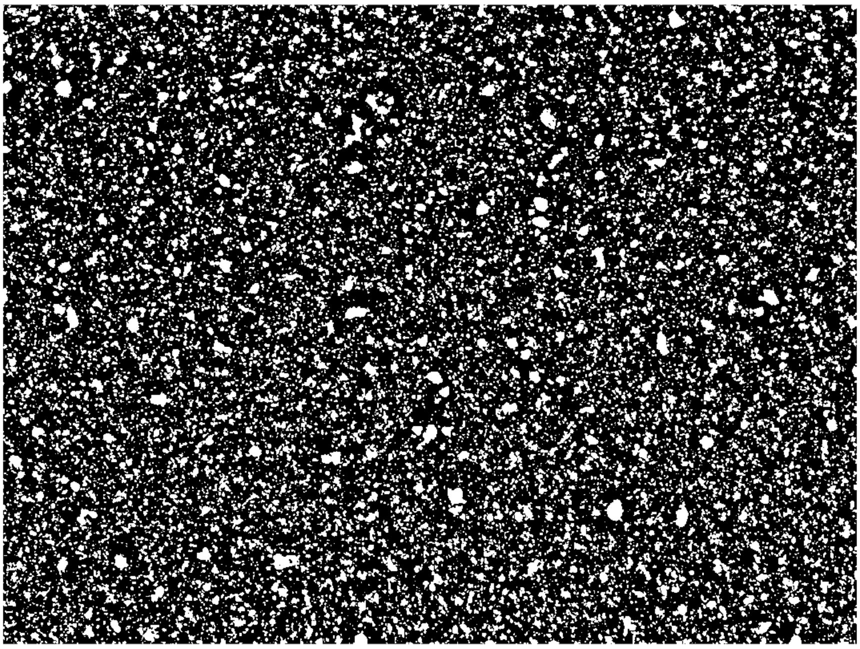

Figure S16b: OM image of MMM-3*, micrograph no.7

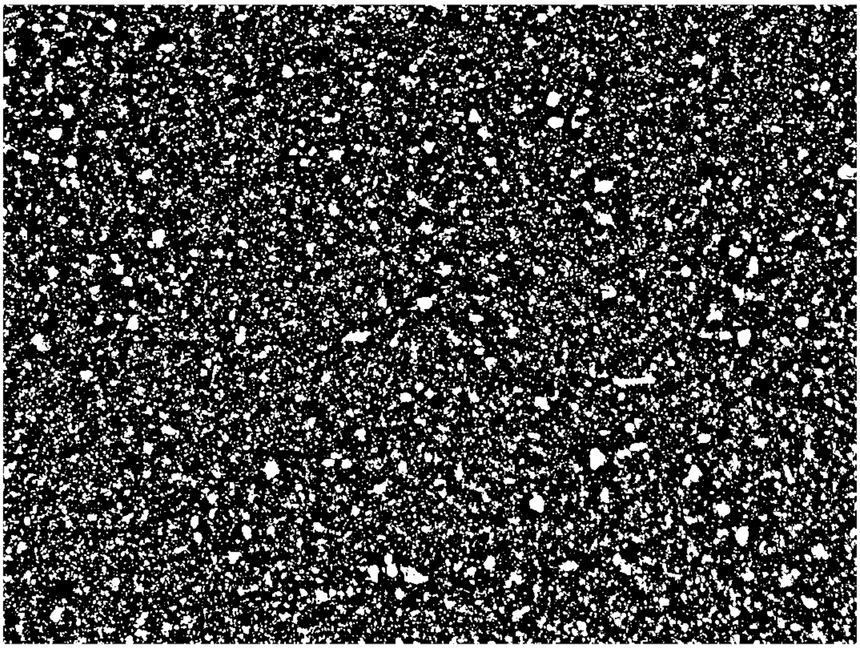

Figure S17b: OM image of MMM-3*, micrograph no.8

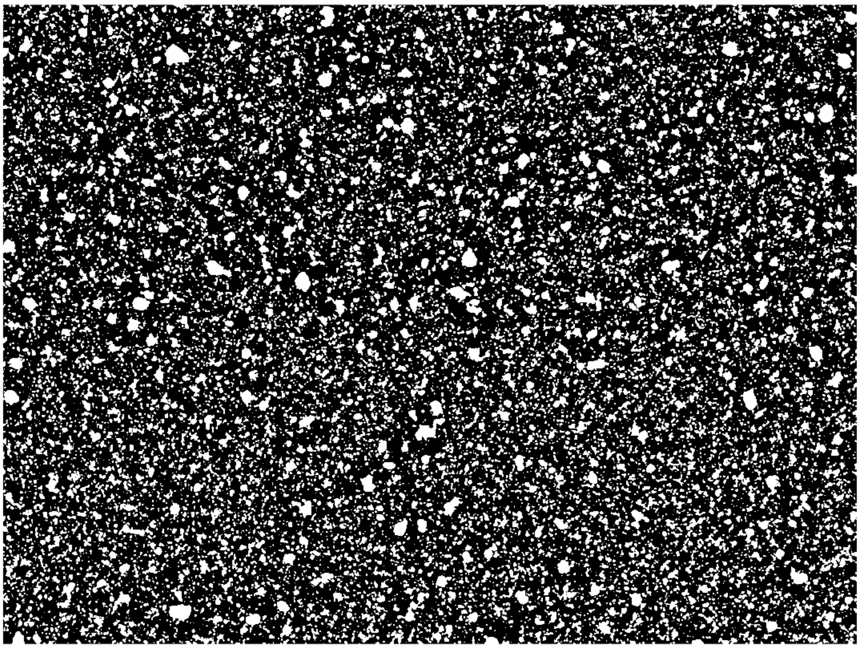

Figure S18b: OM image of MMM-3*, micrograph no.9

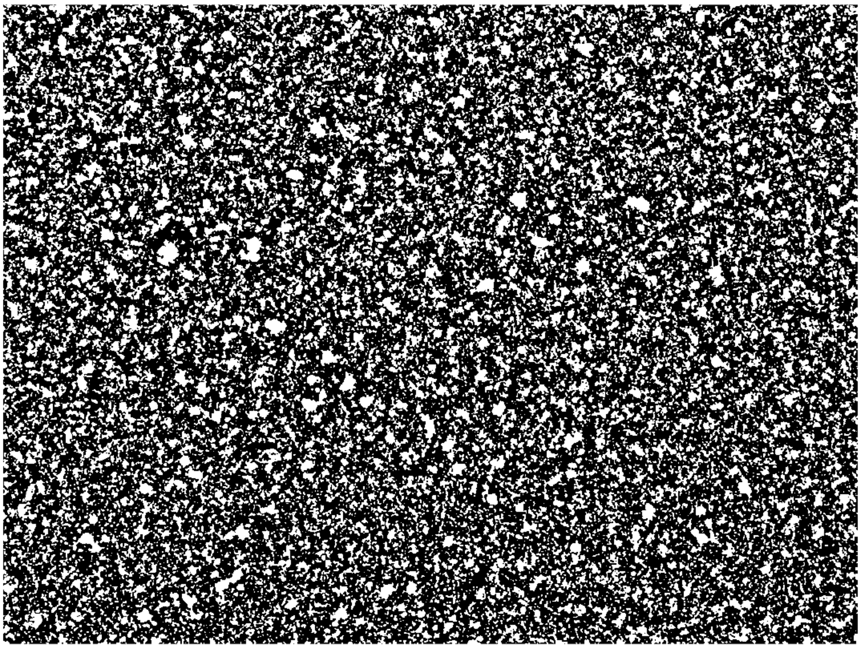

Figure S19b: OM image of MMM-5*, micrograph no.1

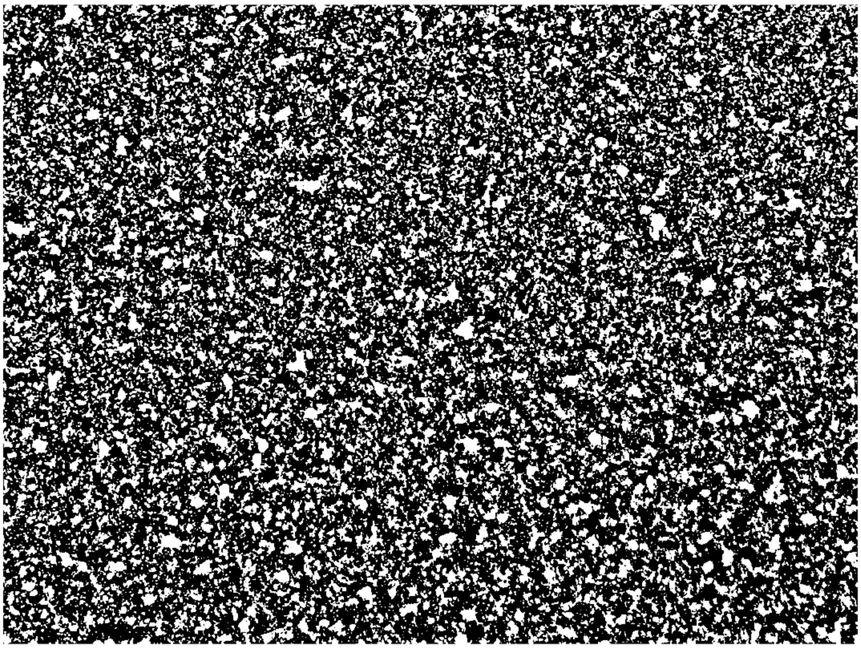

Figure S20b: OM image of MMM-5*, micrograph no.2

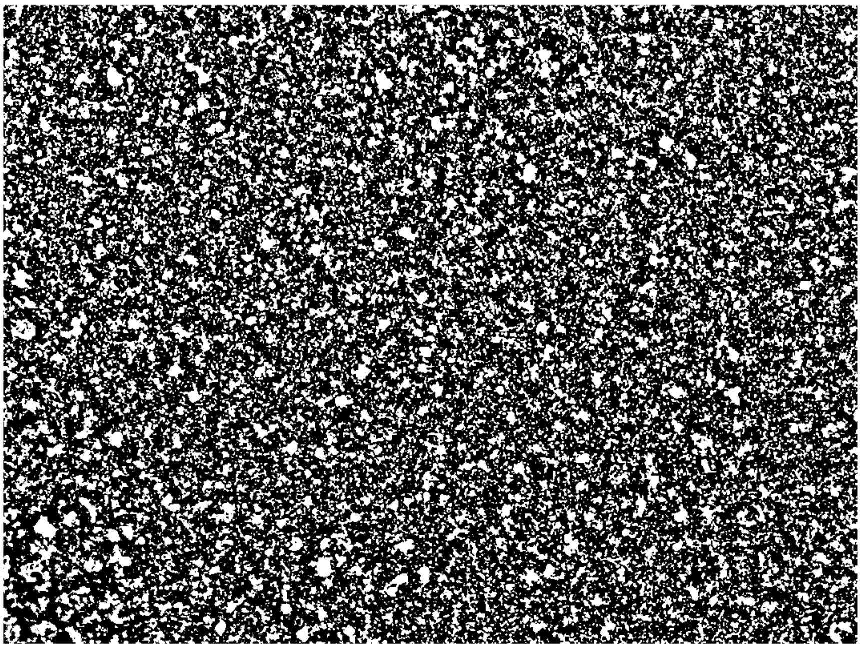

Figure S21b: OM image of MMM-5*, micrograph no.3

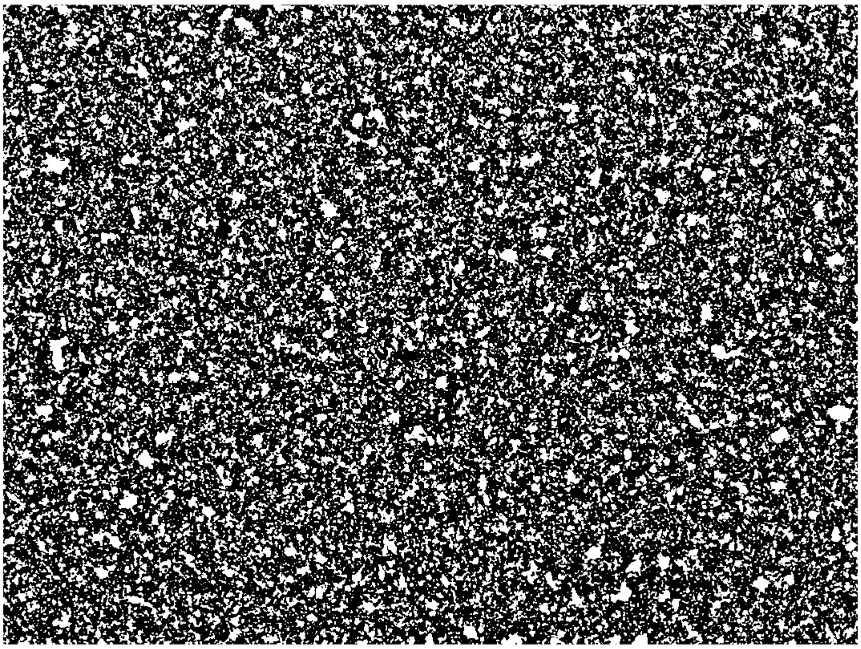

Figure S22b: OM image of MMM-5*, micrograph no.4

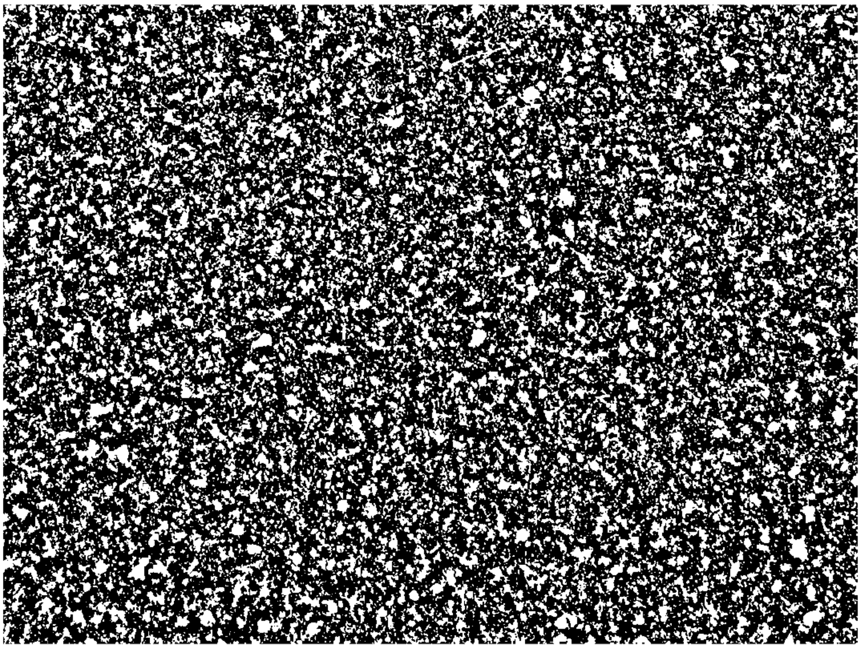

Figure S23b: OM image of MMM-5*, micrograph no.5

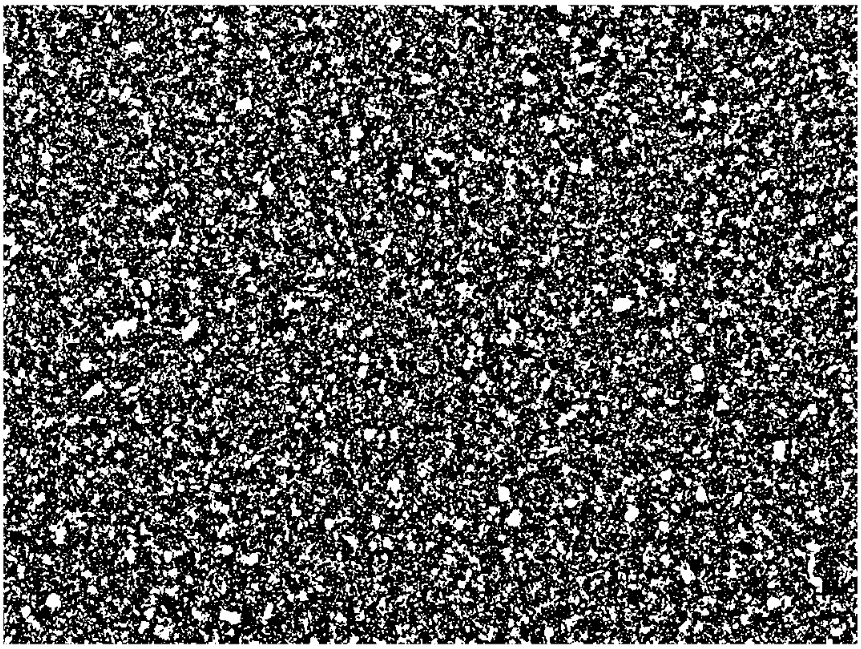

Figure S24b: OM image of MMM-5*, micrograph no.6

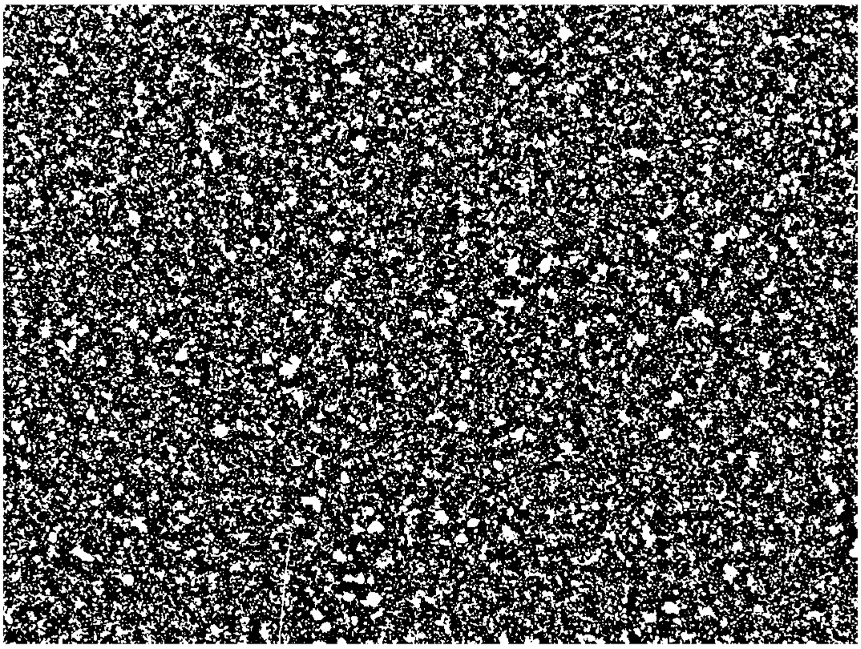

Figure S25b: OM image of MMM-5*, micrograph no.7

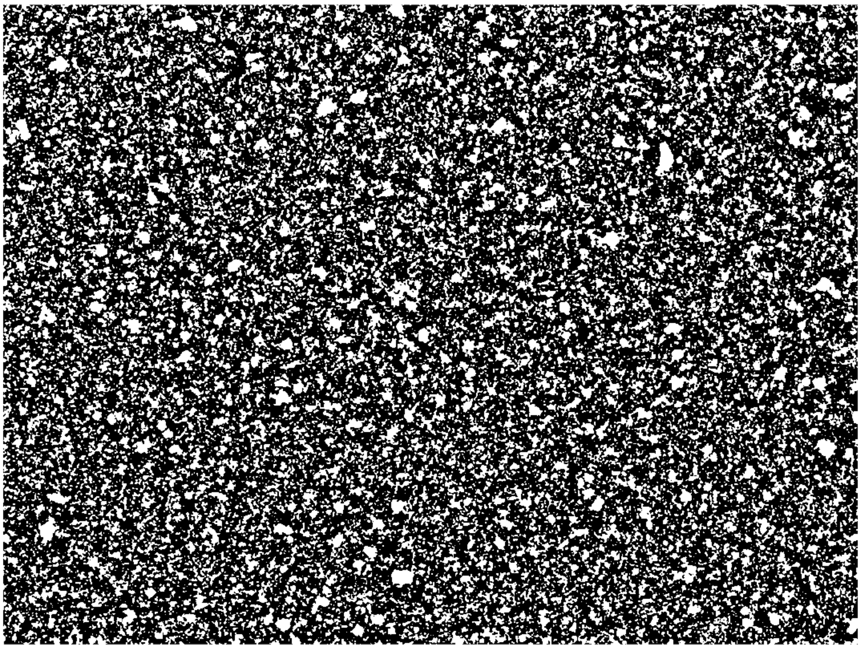

Figure S26b: OM image of MMM-5*, micrograph no.8

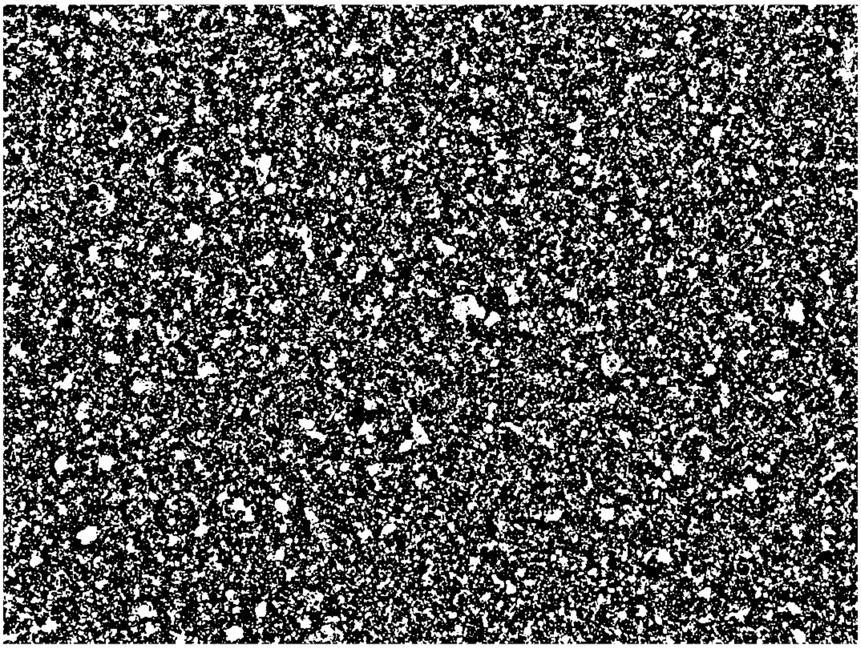

Figure S27b: OM image of MMM-5*, micrograph no.9

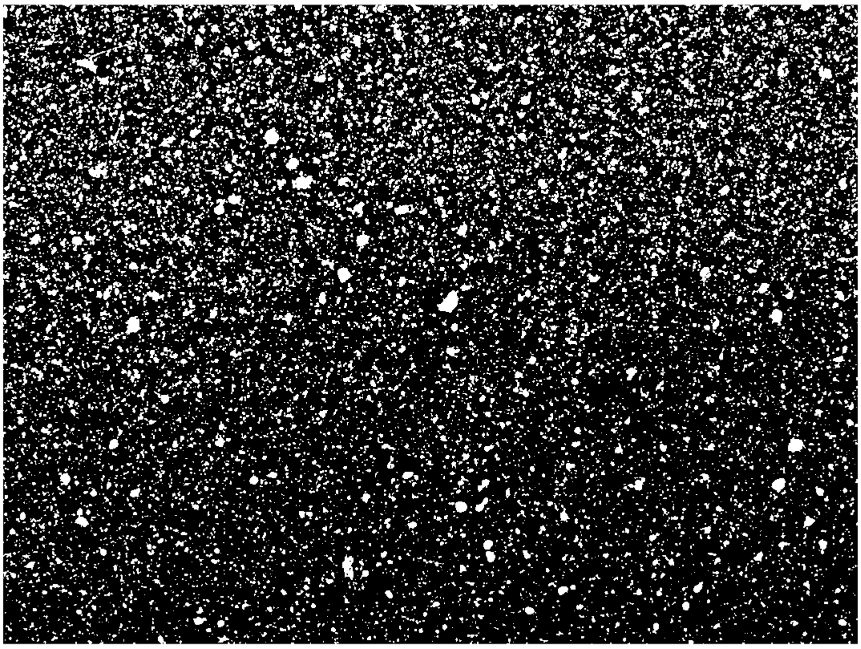

Figure S28b: OM image of MMM-1, micrograph no.1

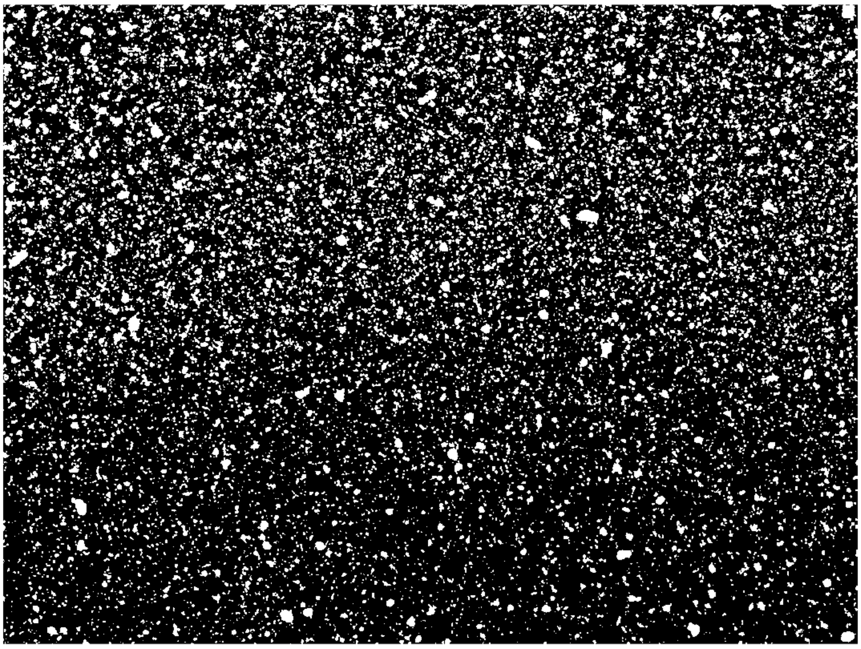

Figure S29b: OM image of MMM-1, micrograph no.2

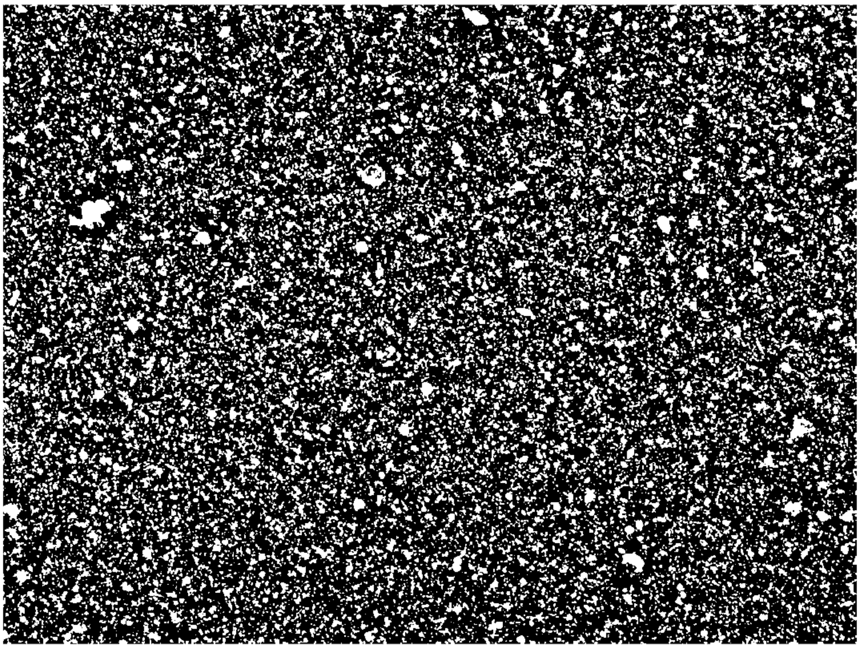

Figure S30b: OM image of MMM-1, micrograph no.3

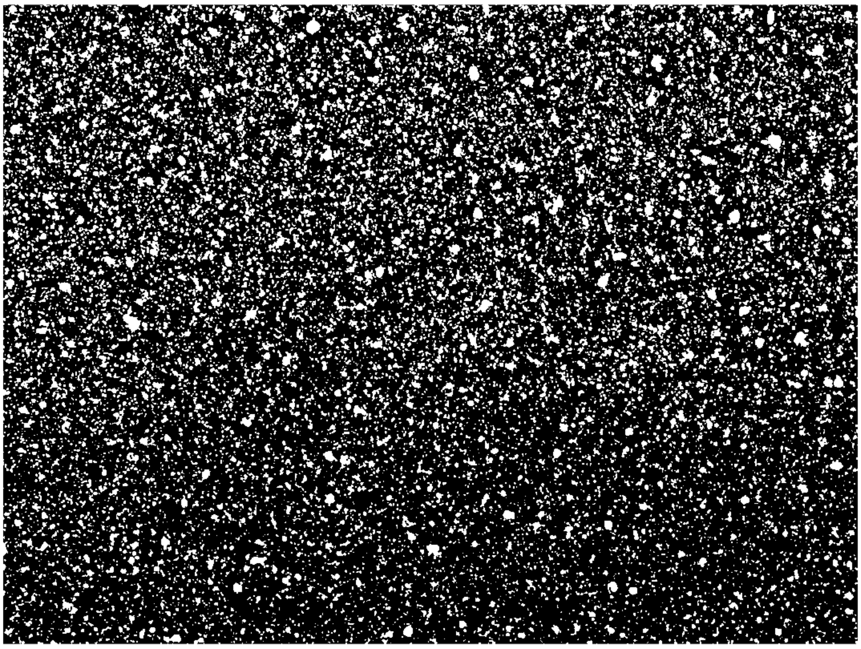

Figure S31b: OM image of MMM-1, micrograph no.4

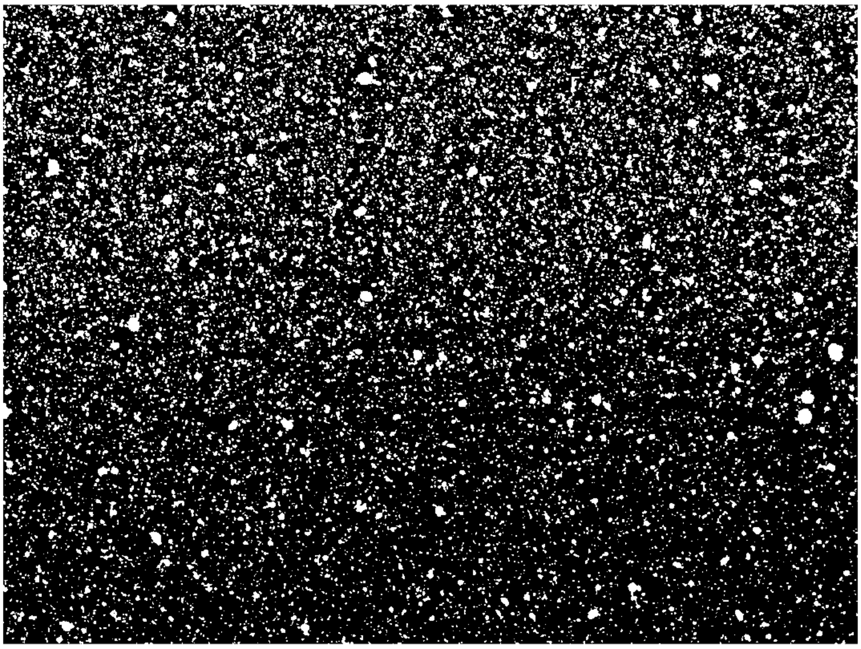

Figure S32b: OM image of MMM-1, micrograph no.5

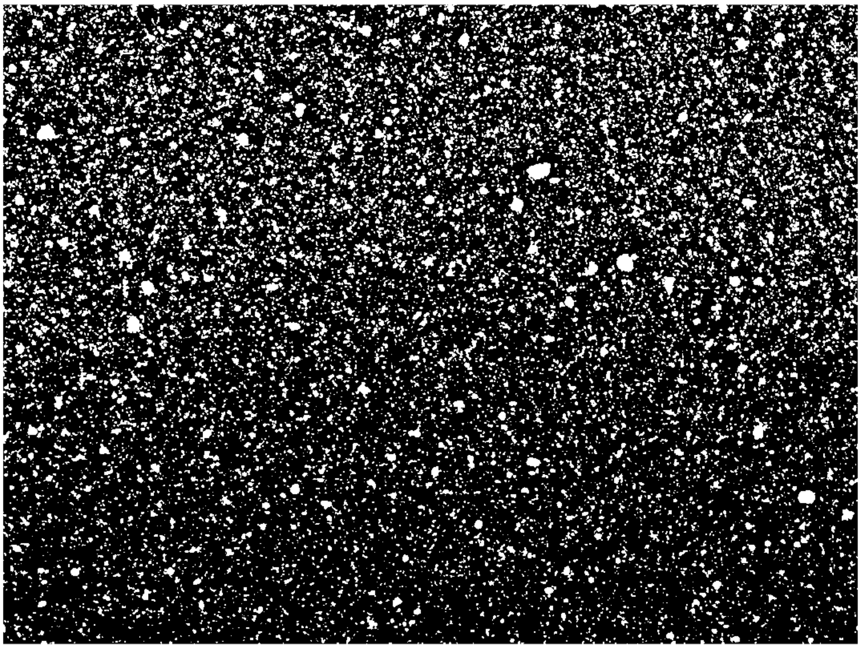

Figure S33b: OM image of MMM-1, micrograph no.6

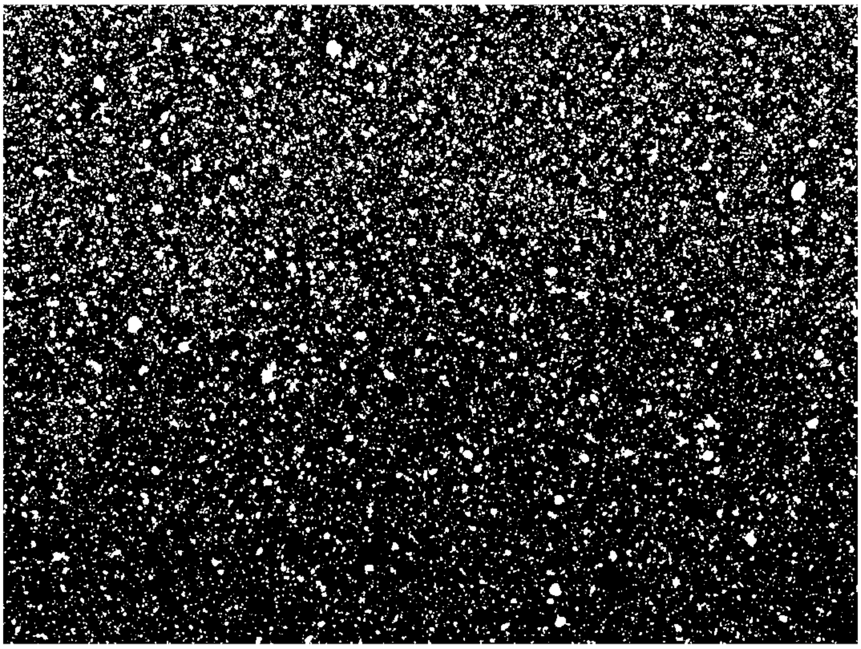

Figure S34b: OM image of MMM-1, micrograph no.7

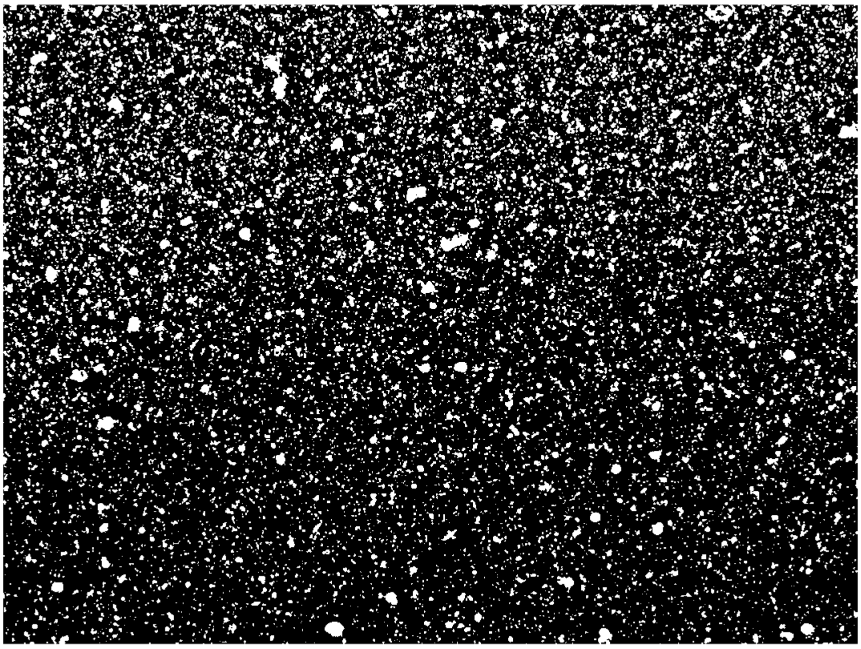

Figure S35b: OM image of MMM-1, micrograph no.8

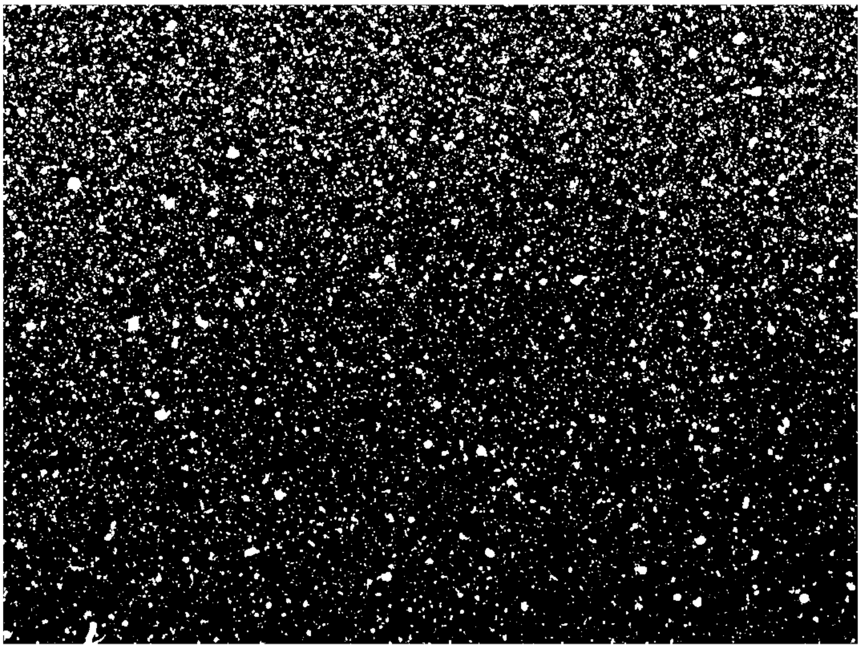

Figure S36b: OM image of MMM-1, micrograph no.9

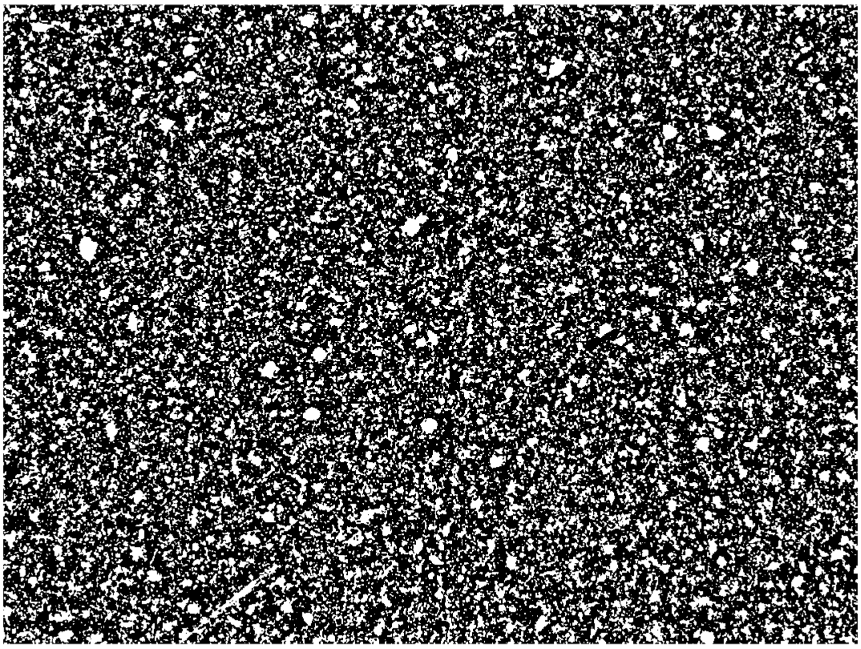

Figure S37b: OM image of MMM-3, micrograph no.1

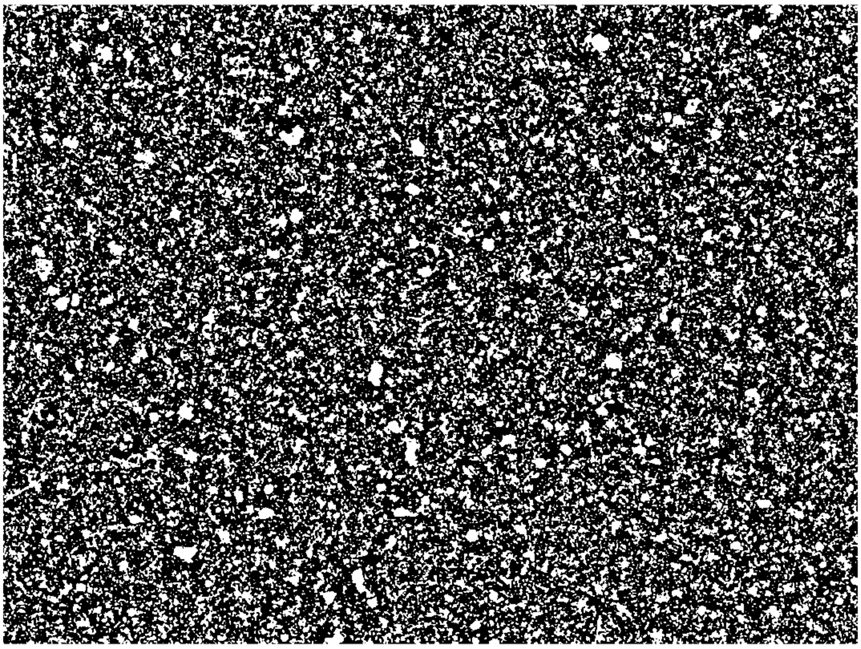

Figure S38b: OM image of MMM-3, micrograph no.2

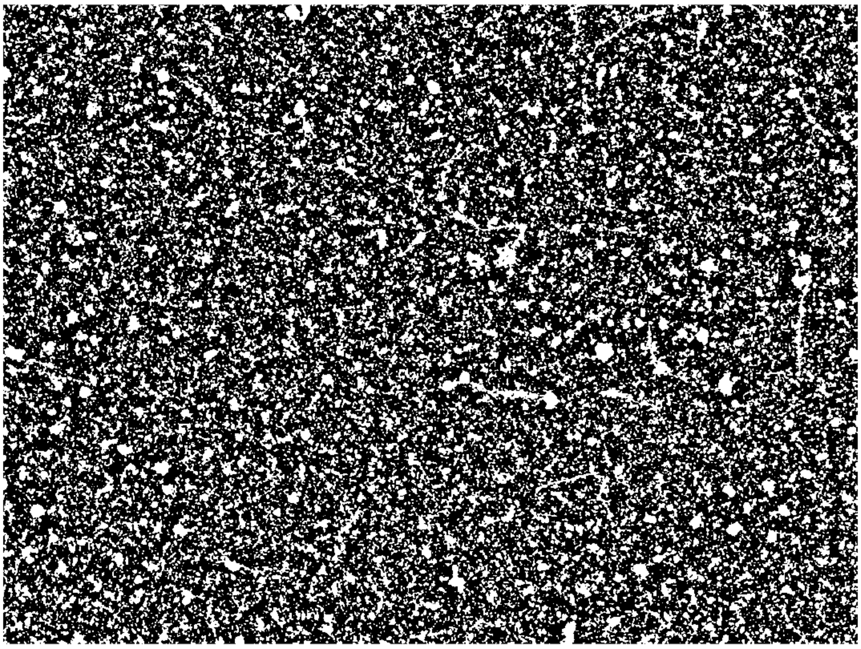

Figure S39b: OM image of MMM-3, micrograph no.3

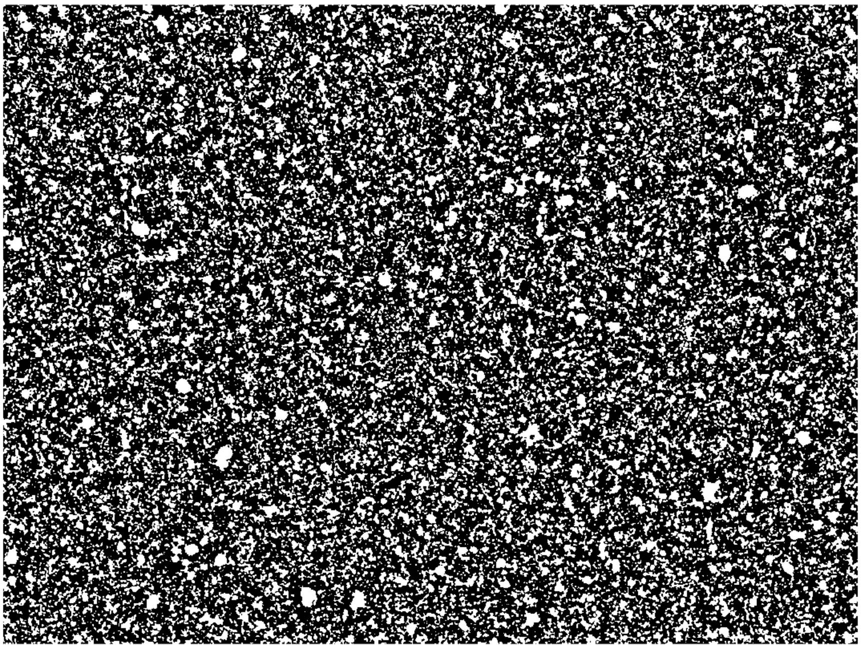

Figure S40b: OM image of MMM-3, micrograph no.4

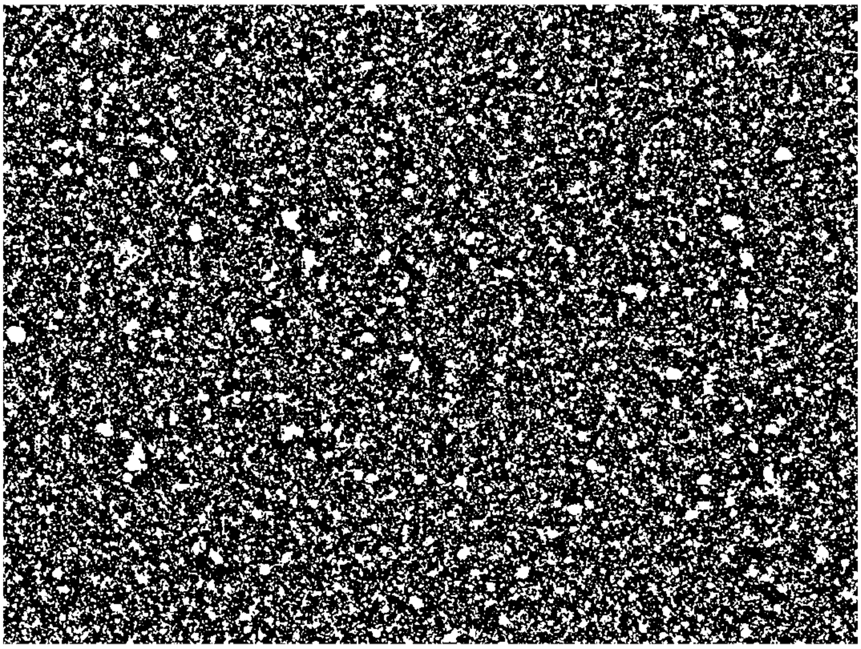

Figure S41b: OM image of MMM-3, micrograph no.5

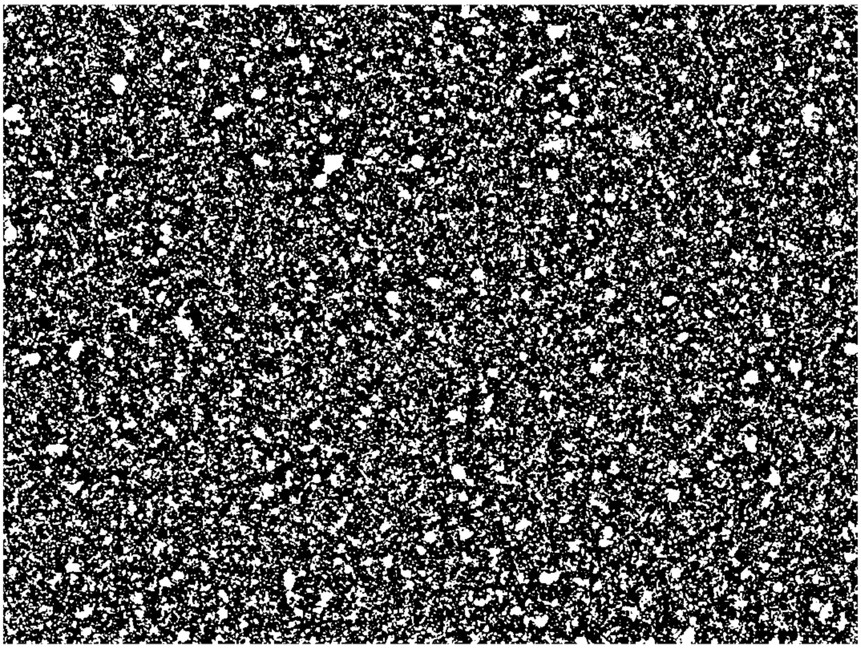

Figure S42b: OM image of MMM-3, micrograph no.6

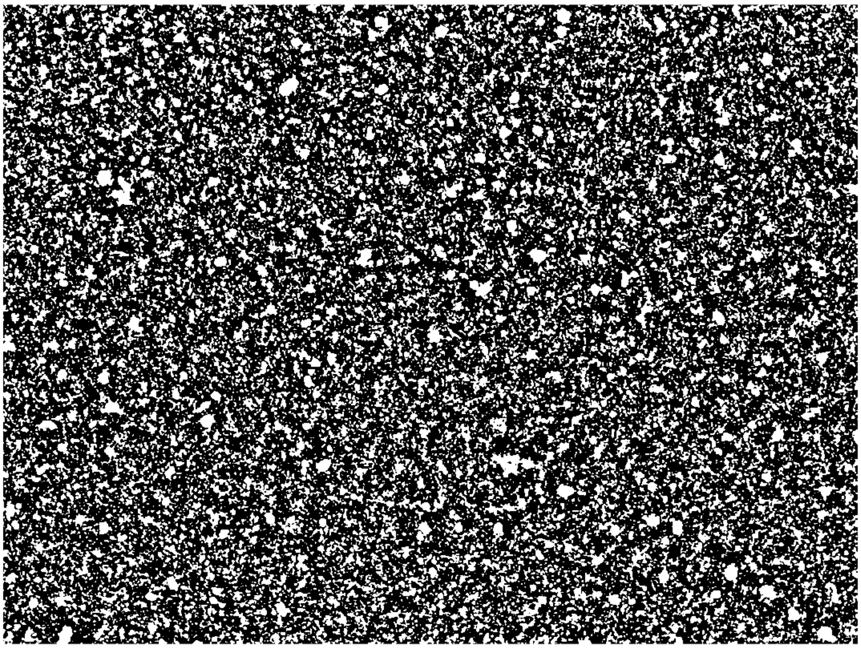

Figure S43b: OM image of MMM-3, micrograph no.7

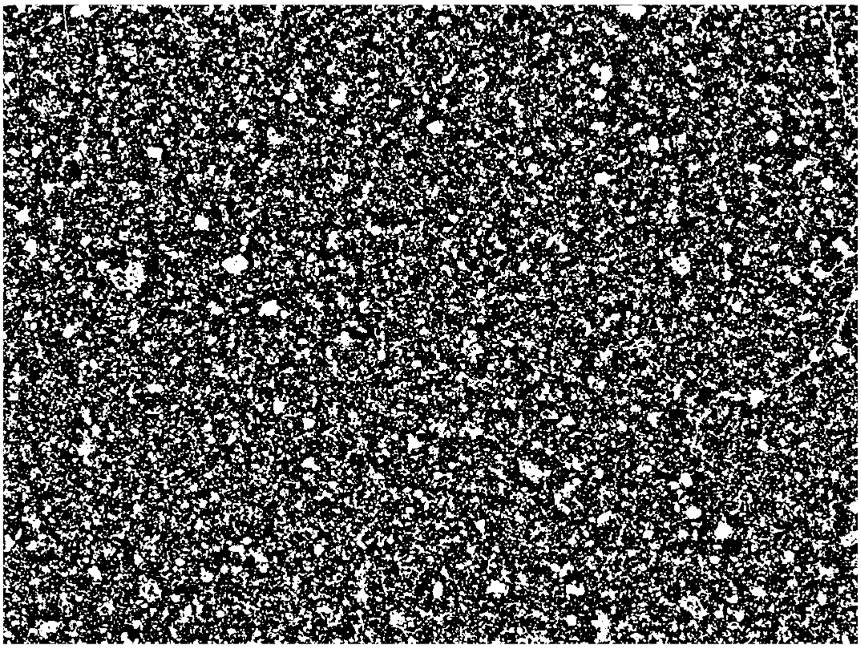

Figure S44b: OM image of MMM-3, micrograph no.8

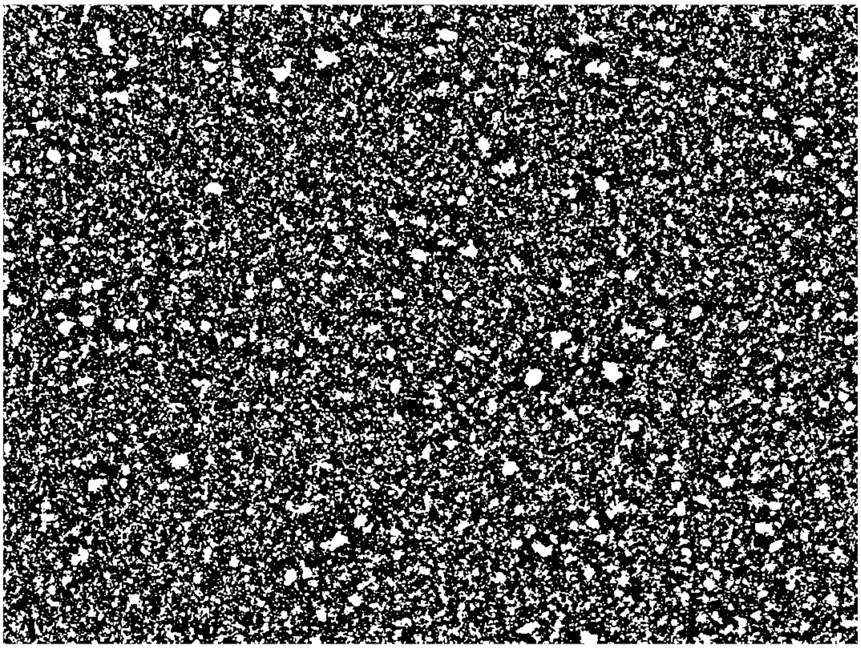

Figure S45b: OM image of MMM-3, micrograph no.9

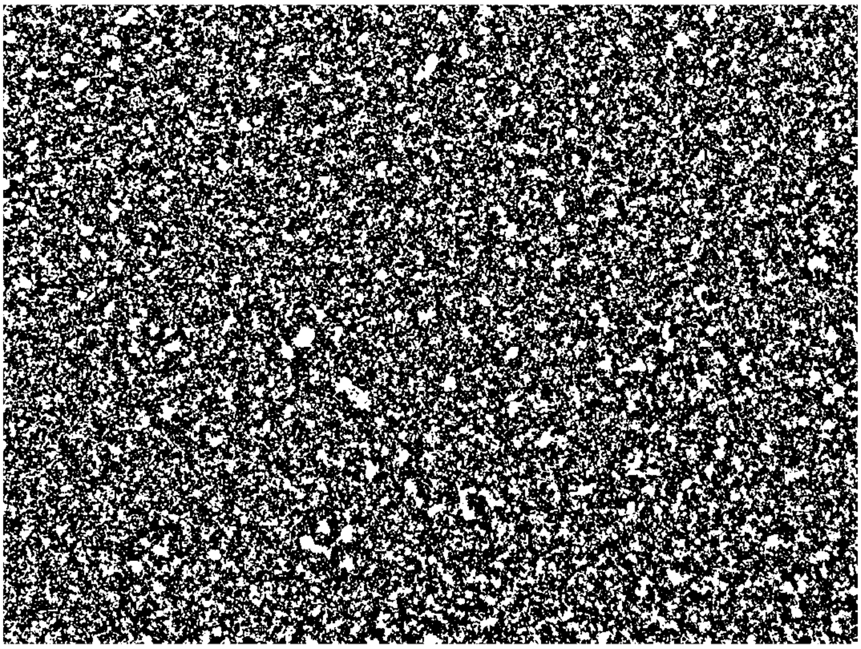

Figure S46b: OM image of MMM-5, micrograph no.1

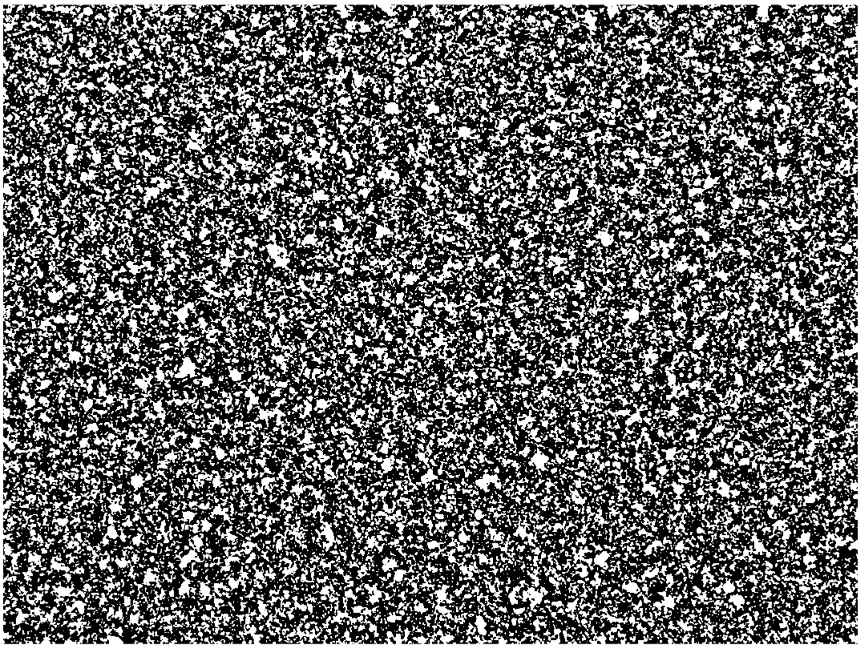

Figure S47b: OM image of MMM-5, micrograph no.2

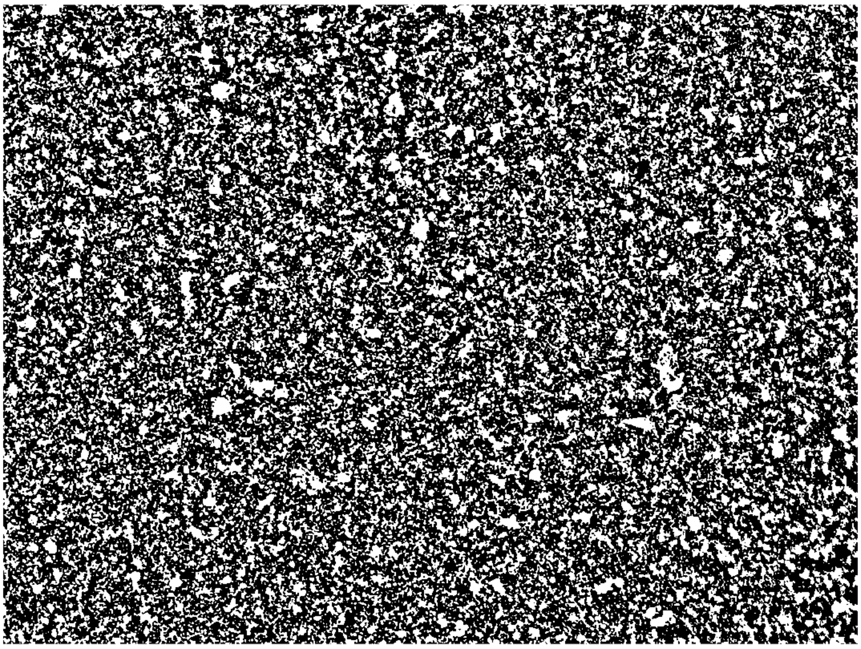

Figure S48b: OM image of MMM-5, micrograph no.3

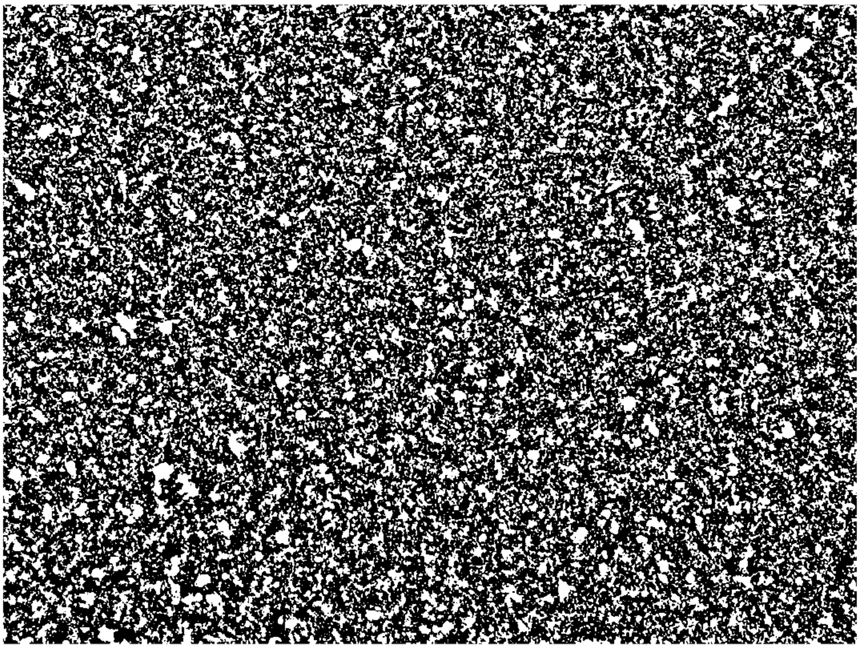

Figure S49b: OM image of MMM-5, micrograph no.4

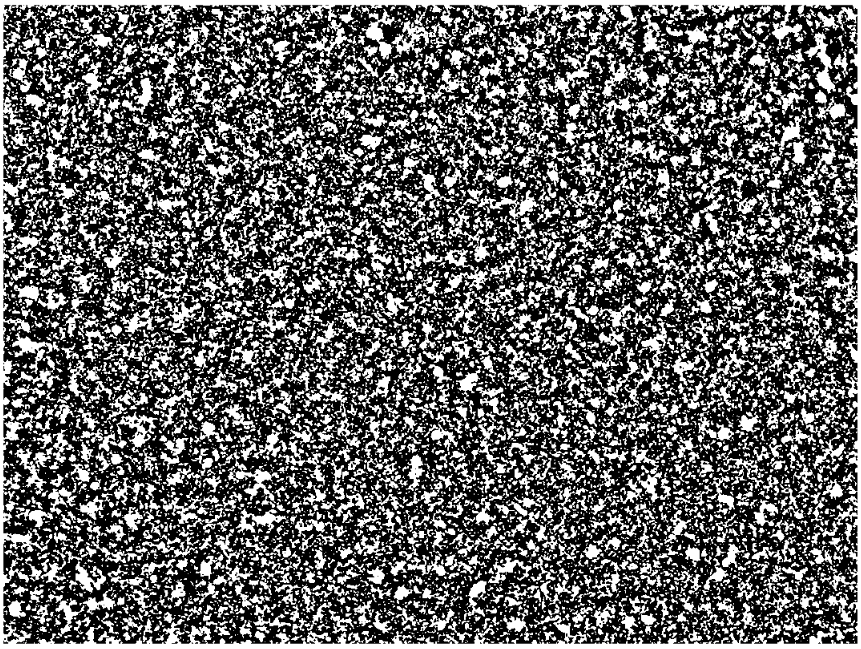

Figure S50 b: OM image of MMM-5, micrograph no.5

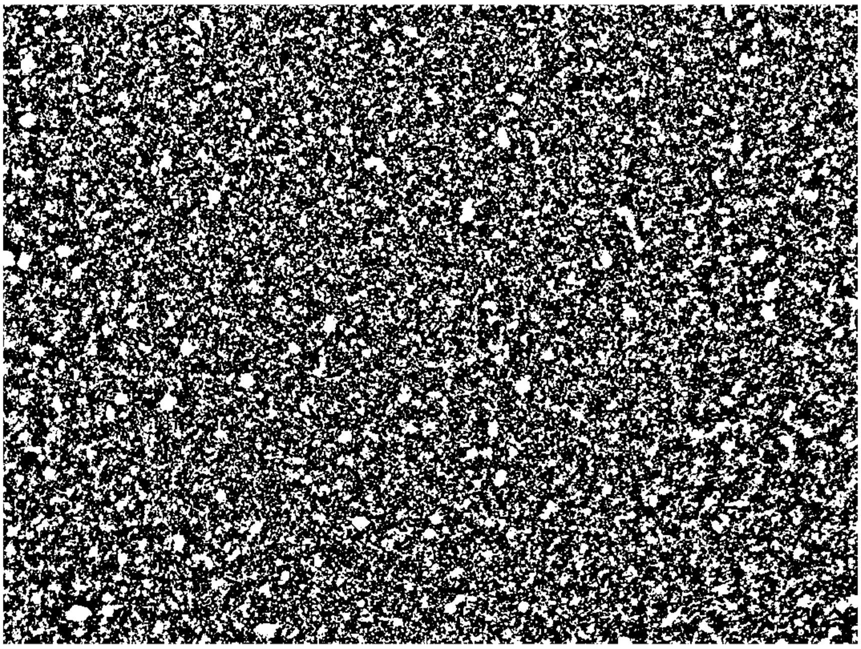

Figure S51b: OM image of MMM-5, micrograph no.6

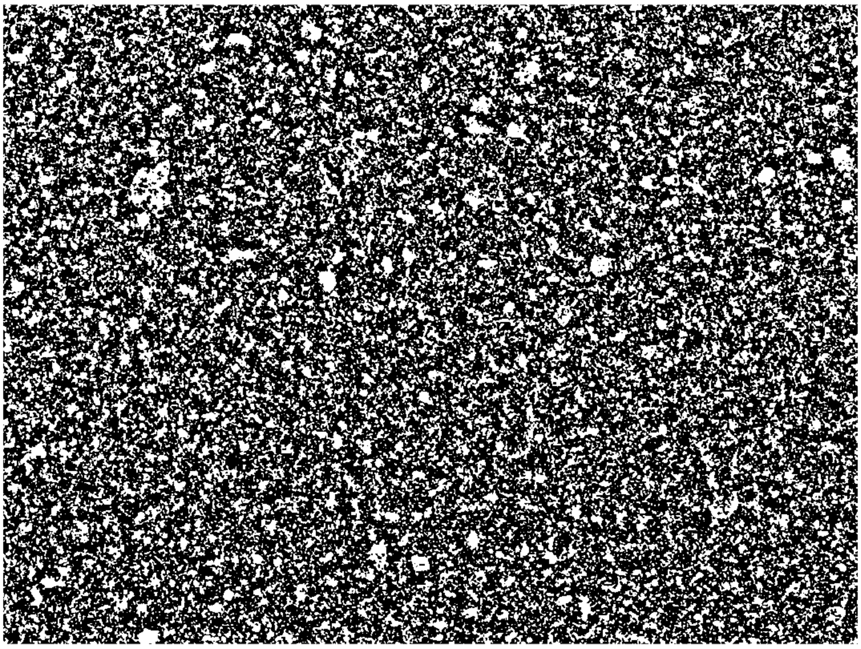

Figure S52b: OM image of MMM-5, micrograph no.7

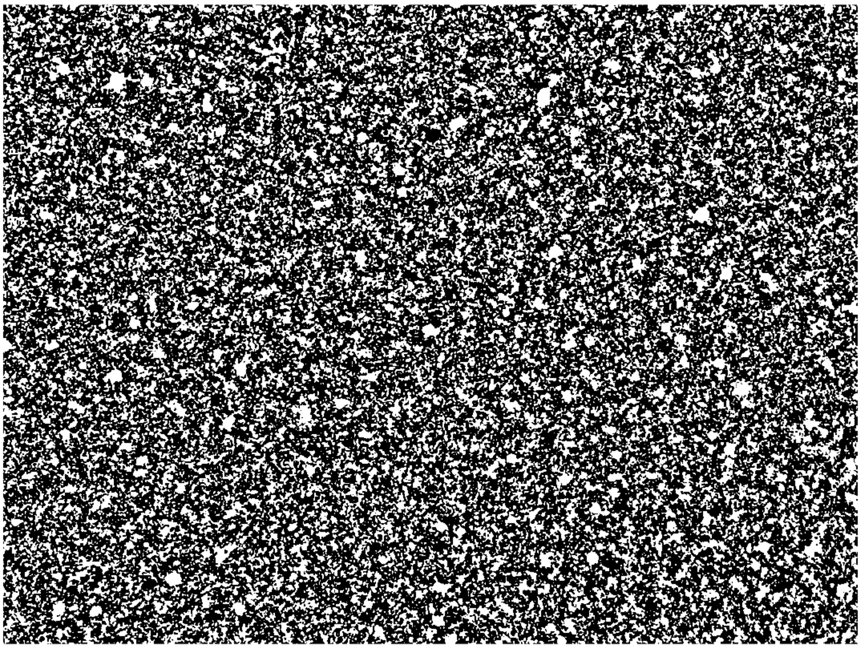

Figure S53b: OM image of MMM-5, micrograph no.8

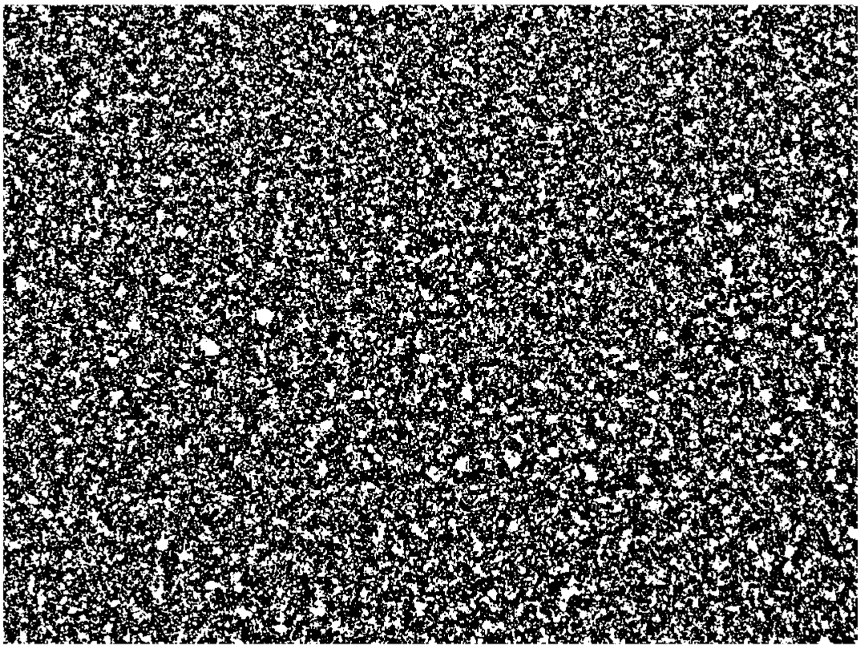

.

Figure S54b: OM image of MMM-5, micrograph no.9
